# Lipin 1 deficiency causes adult-onset myasthenia with motor neuron dysfunction in humans and neuromuscular junction defects in zebrafish

**DOI:** 10.7150/thno.53330

**Published:** 2021-01-01

**Authors:** Shuxian Lu, Zhaojie Lyu, Zhihao Wang, Yao Kou, Cong Liu, Shengyue Li, Mengyan Hu, Hongjie Zhu, Wenxing Wang, Ce Zhang, Yung-Shu Kuan, Yi-Wen Liu, Jianming Chen, Jing Tian

**Affiliations:** 1 Key laboratory of Resource Biology and Biotechnology in Western China, Ministry of Education, School of Medicine, Northwest University, Xi'an 710069, China.; 2 Institute of Oceanography, Minjiang University, Fuzhou 350108, China.; 3 State Key Laboratory of Photon-Technology in Western China Energy, Institute of Photonics and Photon-Technology, Northwest University, Xi'an 710069, China.; 4 Institute of Biochemical Sciences, College of Life Science, National Taiwan University, Taipei, Taiwan.; 5 Department of Life Science, Tunghai University, Taichung, Taiwan.; 6 Sino-French Hoffmann Institute, School of Basic Medical Sciences, Guangzhou Medical University, Guangzhou 511436, China.

**Keywords:** LPIN1, syndromic myasthenia, zebrafish morphants, neuromuscular development, Notch signaling

## Abstract

Lipin 1 is an intracellular protein acting as a phosphatidic acid phosphohydrolase enzyme controlling lipid metabolism. Human recessive mutations in *LPIN1* cause recurrent, early-onset myoglobinuria, a condition normally associated with muscle pain and weakness. Whether and how lipin 1 deficiency in humans leads to peripheral neuropathy is yet unclear. Herein, two novel compound heterozygous mutations in *LPIN1* with neurological disorders, but no myoglobinuria were identified in an adult-onset syndromic myasthenia family. The present study sought to explore the pathogenic mechanism of *LPIN1* in muscular and neural development.

**Methods:** The clinical diagnosis of the proband was compared to the known 48 cases of *LPIN1* recessive homozygous mutations. Whole-exome sequencing was carried out on the syndromic myasthenia family to identify the causative gene. The pathogenesis of lipin 1 deficiency during somitogenesis and neurogenesis was investigated using the zebrafish model. Whole-mount* in situ* hybridization, immunohistochemistry, birefringence analysis, touch-evoke escape response and locomotion assays were performed to observe *in vivo* the changes in muscles and neurons. The conservatism of the molecular pathways regulated by lipin 1 was evaluated in human primary glioblastoma and mouse myoblast cells by siRNA knockdown, drug treatment, qRT-PCR and Western blotting analysis.

**Results:** The patient exhibited adult-onset myasthenia accompanied by muscle fiber atrophy and nerve demyelination without myoglobinuria. Two novel heterozygous mutations, c.2047A>C (p.I683L) and c.2201G>A (p.R734Q) in *LPIN1*, were identified in the family and predicted to alter the tertiary structure of LPIN1 protein. Lipin 1 deficiency in zebrafish embryos generated by *lpin1* morpholino knockdown or human *LPIN1* mutant mRNA injections reproduced the myotomes defects, a reduction both in primary motor neurons and secondary motor neurons projections, morphological changes of post-synaptic clusters of acetylcholine receptors, and myelination defects, which led to reduced touch-evoked response and abnormalities of swimming behaviors. Loss of lipin 1 function in zebrafish and mammalian cells also exhibited altered expression levels of muscle and neuron markers, as well as abnormally enhanced Notch signaling, which was partially rescued by the specific Notch pathway inhibitor DAPT.

**Conclusions:** These findings pointed out that the compound heterozygous mutations in human *LPIN1* caused adult-onset syndromic myasthenia with peripheral neuropathy. Moreover, zebrafish could be used to model the neuromuscular phenotypes due to the lipin 1 deficiency, where a novel pathological role of over-activated Notch signaling was discovered and further confirmed in mammalian cell lines.

## Introduction

Muscle weakness, also known as myasthenia, is a symptom of muscular, neurological and metabolic disorders with a wide variety of mild to severe conditions that arise due to aging, infection, trauma, heart failure, diabetes, malignancy, autoimmune diseases and other abnormal processes. Muscle weakness can occur in any age group and various ethnic backgrounds 1-4. Muscular dystrophy, inflammatory myopathy, neuromuscular disorders, motor neuron diseases and myasthenia gravis are the common genetic causes of muscle weakness 1-3, 5, 6. The underlying molecular processes and genetic disorders that cause muscle weakness are still under intense study.

Lipin 1 (phosphatidic acid phosphatase 1, PAP1, EC 3.1.3.4) 7, encoded by *LPIN1* gene, is a Mg^2+^-dependent phosphatidic acid phosphatase (PAP1) enzyme that controls lipid synthesis and the expression of genes involved in energy metabolism pathways 8, 9. In the cytoplasm, lipin 1 protein acts as PAP1 enzyme converting phosphatidic acid (PA) to diacylglycerol (DAG) for the synthesis of triglycerides (TAG) and phospholipids; while translocated to the nucleus, lipin 1 functions as a transcriptional co-activator through direct interaction with transcription factors to regulate gene expressions 10, 11. Lipin 1 is highly expressed in adipose tissue, skeletal muscle and is also present in other tissues including heart, liver, kidney, brain and peripheral nerve 12, 13, which indicates a complicated role for lipin 1 in development and homeostasis.

Autosomal recessive *LPIN1* mutations in human have been identified as a major cause of recurrent, early-onset myoglobinuria and rhabdomyolysis (MIM#268200), a condition characterized by recurrent attacks of rhabdomyolysis due to damaged skeletal muscle, leading to the excretion of myocellular proteins (including creatine kinase and myoglobin) into the circulatory system. The clinical features are also accompanied by muscle weakness, myalgia, and occasional renal failure 4, 14. The heterozygous mutations of *LPIN1* might cause statin-induced myopathy 15. Association studies between the metabolic syndrome and genetic variants of the *LPIN1* gene revealed that *LPIN1* polymorphisms contribute to several metabolic traits and obesity-related phenotypes which may differ among populations 16, 17. Additional, functions of lipin 1 have also been revealed by a series of studies in mouse models. The whole-body deficiency of lipin 1 caused by spontaneous mutation in the fatty-liver dystrophic mouse (*Lpin1^fld/fld^*) was identified by progressive lipodystrophy 18, peripheral neuropathy and skeletal muscle myocyte damage 19, 20. Furthermore, by a conditional knockout of *Lpin1* in Schwann cells (SCs) in mouse (*MPZ^cre+^/Lp^fE2-3/fE2-3^*), Nadra *et. al.* 9 demonstrated that ablation of *Lpin1* is sufficient to cause peripheral neuropathy; as well as endoneurial accumulation of phosphatidate in adipose tissues and peripheral nerves, which in turn lead to activation of MEK-Erk pathway and demyelination in SCs. Schweitzer *et. al.* 21 generated two muscle-specific LPIN1-deficienct mice models (*MCK-Lpin1^△115^* and *MCK-Lpin1^-/-^*), and demonstrated that either selective loss of PAP activity or complete deletion of LPIN1 protein in skeletal muscles leads to chronic myopathy, myofibrillar degeneration, increased phosphatidic acid levels, mitochondrial dysfunction and impaired autophagy. These studies supported a critical role of lipin1, in addition to its biochemical functions in lipid synthesis and energy metabolism, in peripheral nerve and skeletal muscle development. However, whether and how lipin 1 mediates the formation, maintenance and function of neuromuscular junction (NMJ) remains unclear. Moreover, it remains elusive whether lipin 1 deficiency in human adults would cause peripheral neuropathy as in the mouse model.

In this study we identified two novel compound heterozygous variants, c.2047A>C (p.I683L) and c.2201G>A (p.R734Q) of *LPIN1* from a syndromic myasthenia family. The two mutations were predicted to be damaging and disease-causing by *in silico* analysis. Unlike the reported human *LPIN1* mutations which cause myoglobinuria and rhabdomyolysis mostly in early-onset pediatric form; the proband in this study presented adult-onset severe mixed peripheral neuropathy, neurogenic damage of skeletal muscle, and progressive muscle weakness, albeit without myoglobinuria or rhabdomyolysis. In order to explore the mechanisms underlying LPIN1 functions in muscle and neuron development and the potential signaling pathways involved, a zebrafish model of lipin 1 deficiency was established. By *lpin1* morpholino knockdown, or overexpression of human point mutation* LPIN1* mRNA, Lpin1*-*deficient zebrafish embryos exhibited myopathic phenotype with myotomes defects caused by myofibrillar disorganization, reduction of primary motor neurons (PMNs) and secondary motor neurons (SMNs) projections, morphological changes in the post-synapse, and myelination defects, leading to reduced touch-evoked response and mobility, which partially phenocopied human *LPIN1* mutations and *Lpin1* mouse models. Notably, we found that the Notch pathway was activated upon lipin 1 knockdown in zebrafish and mammalian cells, and the abnormally increased Notch signaling could be partially rescued by gamma-secretase inhibitor DAPT. These results indicated an essential and evolutionarily conserved role of lipin 1 in the neuromuscular system, and suggested a novel mechanism underlying the pathology of LPIN1-related neuromuscular disorders.

## Materials and Methods

### Clinical assessment

The study was carried out in accordance to the Declaration of Helsinki 22. Written informed consent was obtained from all participants before their participation in this study. Individual II-5 of the family was initially diagnosed at the Peking University First Hospital in China. All human studies were approved by the Review Board of Northwest University.

### Whole-exome sequencing

Genomic DNA was extracted from peripheral blood (II2, II3, II4, II5) and saliva samples (II1, II6, III1, III2, III3) by standard procedures. Whole-exome capture of subjects II2, II3, II4, II5 was performed using Agilent SureSelect XT Human All Exon v.4 kit, sequenced by 100nt paired-end reads on Illumina HiSeq2000 platform. The reads provided in FASTQ files were mapped to the human genome (hg19) using Burrows-Wheeler Aligner (BWA) 23. Variants were called using SAMtools program and duplicated reads were marked by Picard 24. All variants were screened with the SNP database version 142 (dbSNP142), 1000 Genomes Project (version 2014 October), and NHLBI Exome Sequencing Project (ESP) 6500. Functional prediction was carried out by Sorting Intolerant from Tolerant (SIFT), Polymorphism Phenotyping version 2 (PolyPhen-2), MutationTaster and MutationAssessor, and the remaining rare variants were annotated using ANNOVAR to separate non-synonymous substitutions, indel variants and splicing mutations. Further analysis was carried out by ModelR and Phenolyzer to screen the candidate variants in the transcribed regions.

### Sanger sequencing and* in silico* analyses

Segregation validation was performed by direct Sanger sequencing in all available family members using specific oligonucleotide primers flanking the exons with ABI3500 sequencer (Applied Biosystems, USA). New *LPIN1* variants were further screened in at least 100 unrelated healthy controls. The primer sequences were listed in Table S1. Sequence analysis was carried out using BigDye Terminator Cycle Sequencing Kit and PCR products were run on ABI PRISM 3730 Analyzer (Applied Biosystems, USA). *In silico* algorithms for predicting deleteriousness of nonsynonymous mutations were applied using dbNSFP. MultAlin was used for multiple sequence alignment. Crystal structural models of wild-type (wt) and mutant LPIN1 were constructed using the SWISS-MODEL online server. The predicted structures were displayed by PyMol software (version 1.5).

### Plasmids construction and mRNA synthesis

Human *LPIN1* cDNA (NM_145693.4) was amplified and cloned into vectors pCDNA3.1+ and pCS2+. Two missense mutations, p.I683L and p.R734Q, were introduced into *hLPIN1* plasmid by site-direct mutagenesis PCR. Full length of zebrafish *lpin1* cDNA (NM_001044353.1) was amplified and cloned into pCS2+ vector. All generated plasmids were validated by Sanger sequencing. Capped mRNA of human *LPIN1^wt^*, *LPIN1^ I683L^*, *LPIN1^ R734Q^*, *LPIN1^ I683L& R734Q^* and zebrafish *lpin1* was generated using a mMESSAGE mMACHINE SP6 Transcription kit (Thermo Fisher Scientific, USA).

### Zebrafish maintenance

All adult zebrafish, including AB strain and transgenic line *Tg(mbp:eGFP)* 25, were raised according to standard protocols 26. Embryos were obtained by natural crosses and staged as described previously 27. To inhibit pigmentation embryos were treated with 0.03% 1-phenyl-2-thiourea (Sigma). All experimental procedures for zebrafish were carried out in accordance with the approved guidelines by the Experimental Animal Management and Ethics Committees of Northwest University.

### Microinjection of morpholinos (MOs) and mRNA

Antisense MO oligomers of zebrafish *lpin1*, a splicing-donor MO (*lpin1* MO) and standard control MO (STD-MO) were obtained from Genetools, LLC (USA). The nucleotide sequences of the MOs were given in Table S1. MOs were resuspended in 1 X Danieau solution to a concentration of 2 mM stock solution before further dilutions into the required concentrations. 0.2 pmol *lpin1* MO, 1.2 pmol STD-MO, 200 pg human wt or mutant *LPIN1* mRNA was injected into each embryo at the 1-cell stage, respectively, using PLI-100A Plus Pico Injector (Harvard, USA).

### Analysis of skeletal muscle structure by birefringence

For scoring the skeletal muscle lesions, 4 day-post-fertilization (dpf) embryos were anaesthetized with tricaine (0.04%), embedded in 5% methyl cellulose, and viewed under SMZ25 stereomicroscope equipped with a DS-Ri2 digital camera (Nikon, Japan). Birefringence was imaged as previously described 28. For quantification analysis, 10 somites between the levels of somite 5 to 15 were imaged per embryo.

### Whole-mount *in situ* hybridization (ISH) and immunohistochemistry (IHC)

Digoxigenin (DIG)-labeled probes of *lpin1*, *deltaC*, *actn3a*, *neurog1* and *isl1* were synthesized using SP6/T7 RNA Labeling Kit (Roche Diagnostics, USA). Zebrafish embryos at different stages were collected and fixed in 4% paraformaldehyde, dehydrated in methanol and stored at -20 °C until use. Whole-mount ISH assays were performed as described previously 29, 30. Stained embryos from the ISH assay were cleared in 50% glycerol in PBS and photographed under Nikon SMZ25 microscope system.

*In vivo* IHC was performed on whole embryos according to the described protocol 29, 31. To detect Lpin1 protein, fixed embryos were permeabilized with PBST, incubated with rabbit anti-LPIN1 antibody at 1:200 and mouse anti-acetylated tubulin antibody at 1:400 (Sigma, USA), followed by incubation with Alexa568-conjugated goat anti-rabbit IgG (Invitrogen) and Alexa488-conjugated goat anti-mouse IgG (Invitrogen, USA) as the secondary antibody at 1:200 dilution. To label acetylcholine receptors (AChR) clusters, embryos were incubated in Alexa fluor 488-conjugated α-Bungarotoxin (α-BGT) at 1:100 (Invitrogen, USA). Primary motoneurons (PMN) were labeled with mouse anti-Znp1 antibody (DSHB, USA) and secondary motoneurons (SMNs) were labeled with mouse anti-Zn8 antibody (DSHB, USA), followed by incubation with Dylight 594-conjugated goat anti-mouse IgG (Abcam, UK) at 1:200 dilution. The images were captured under a Leica TCS SP5 confocal microscope with Leica LAS AF Lite 4.0 software.

### Touch-evoked escape response assays and tracking of swimming behavior

Touch-evoked escape response was tested in 4 dpf embryos and recorded under Nikon SMZ25 stereomicroscope equipped with a DS-Ri2 digital camera system, the images were processed using NIS Elements software 28.

Swimming behavior was monitored and locomotion was tracked using a DanioVision system and EthoVision XT 11.5 locomotion tracking software (Noldus Information, Netherlands) 32. Zebrafish larvae (injected with morpholino or mRNA) developed to 5 dpf were placed in individual wells of 24-well cell culture plate containing embryo medium and monitored at room temperature (22 °C). For startle response analysis, one cycle included a 20-second (s) spontaneous movement tracking followed by cycle stimulation with light on for 5 s, interval 1 s, trigger tap 1 s, and interval 48 s. The distance, movement, and velocity parameters for individual locomotion plots were analyzed using the EthoVision XT 11.5 software.

### RNA interference

Human glioblastoma U87 and mouse myoblast C2C12 cells were maintained in DMEM (Gibco, USA) supplemented with 10% FBS (Gibco, USA) and 50 units/mL streptomycin and penicillin (Gibco, USA) at 37 °C. Small interfering RNA (siRNA) duplexes targeting human and mouse* LPIN1/Lpin1* genes were synthesized by GenePharma (China). Cells were transfected with two LPIN1 siRNAs for each cell line or standard siRNA as a negative control by Lipofectamine 3000 (Invitrogen, USA) according to the manufacturer's instructions. siRNA sequences were listed in Table S1.

### Luciferase assays and Western blot analysis

HEK293T cells were seeded at 800,000 cells/well in 6-well plates. To evaluate the Notch signaling pathway activity, transfections were performed in triplicates with pGL4.10-HES1 reporter system and the indicated expression plasmids with the following amounts in each well: 2500 ng of wt or mutant *LPIN1*, 2500 ng of pGL4.10-HES1 vector, and 20 ng of pRL-TK (Renilla). At 2 days after transfection, cells were lysed and luciferase activity was measured with the use of the Dual-Luciferase Reporter Assay Kit (Promega, USA) and luminometer (Molecular Devices, USA). Each transfection was also measured in triplicates. For *in vivo* luciferase assay in zebrafish, 0.2 nM *lpin1* MO or 200 ng pCS2-*lpin1* with 60 ng pGL4.10-*her1* and 5 ng pRL-TK were co-injected into one-cell stage embryos. Each experiment was performed in triplicates.

Western blot analysis was carried out according to the standard protocol described previously 33. The following antibodies were used: rabbit anti-LPIN1 (1:1000, Abcam, UK), mouse anti-Actin (1:5000, TransGen Biotech, China).

### Drug treatment

DAPT (MedChem Express, USA), an effective inhibitor of the Notch pathway, was dissolved at a concentration of 10 mM in DMSO as stock. STD-MO or *lpin1* MO was injected into zebrafish embryos at the one-cell stage. 50 μM DAPT was added at the sphere stage and an equal amount of DMSO was used as the control.

### Quantitative Real-Time PCR (qRT-PCR) analysis

Total RNA was isolated from U87 cells, C2C12 cells and zebrafish embryos using TRIzol^TM^ reagent (Ambion, USA). 1 μg of RNA was reverse transcribed using SuperScript^TM^ system (Invitrogen). qRT-PCR was performed as described previously with SYBR Green PCR Master Mix (Kapa Biosystems, USA) 34. Primer sequences are listed in Table S1. Image J software was used for densitometric measurement.

### Statistical analysis

Each experiment was repeated at least three times and all data were expressed as means ± SEM. Student's t-test was applied for comparisons among different groups. Differences with the* p* value < 0.05 were considered significant.

## Results

### Two novel heterozygous mutations in *LPIN1* were identified from a familial adult-onset muscle weakness proband

The proband (II5) in the family was diagnosed in the hospital for muscle weakness and convulsions lasting 17 years since he was 27-year-old. Laboratory evaluation revealed high blood creatine phosphokinase (CPK) (22013 UI/L; normal<150 UI/L), as well as elevated levels of alanine aminotransferase (ALT) and aspartate aminotransferase (AST). Skeletal muscle biopsy revealed muscle fibers with an abnormal lipid overload, the predominance of type I muscle fibers, type II muscle fibers atrophy, and mitochondria of muscle fibers exhibited morphological abnormality. Furthermore, electromyography disclosed a slowed gastrocnemius sensory nerve conduction velocity, and histology analysis of the upper limb muscles exhibited neurogenic damage with severely nerve demyelination (Table 1). The proband's eldest brother (II2) had similar symptoms.

**Figure 1:**
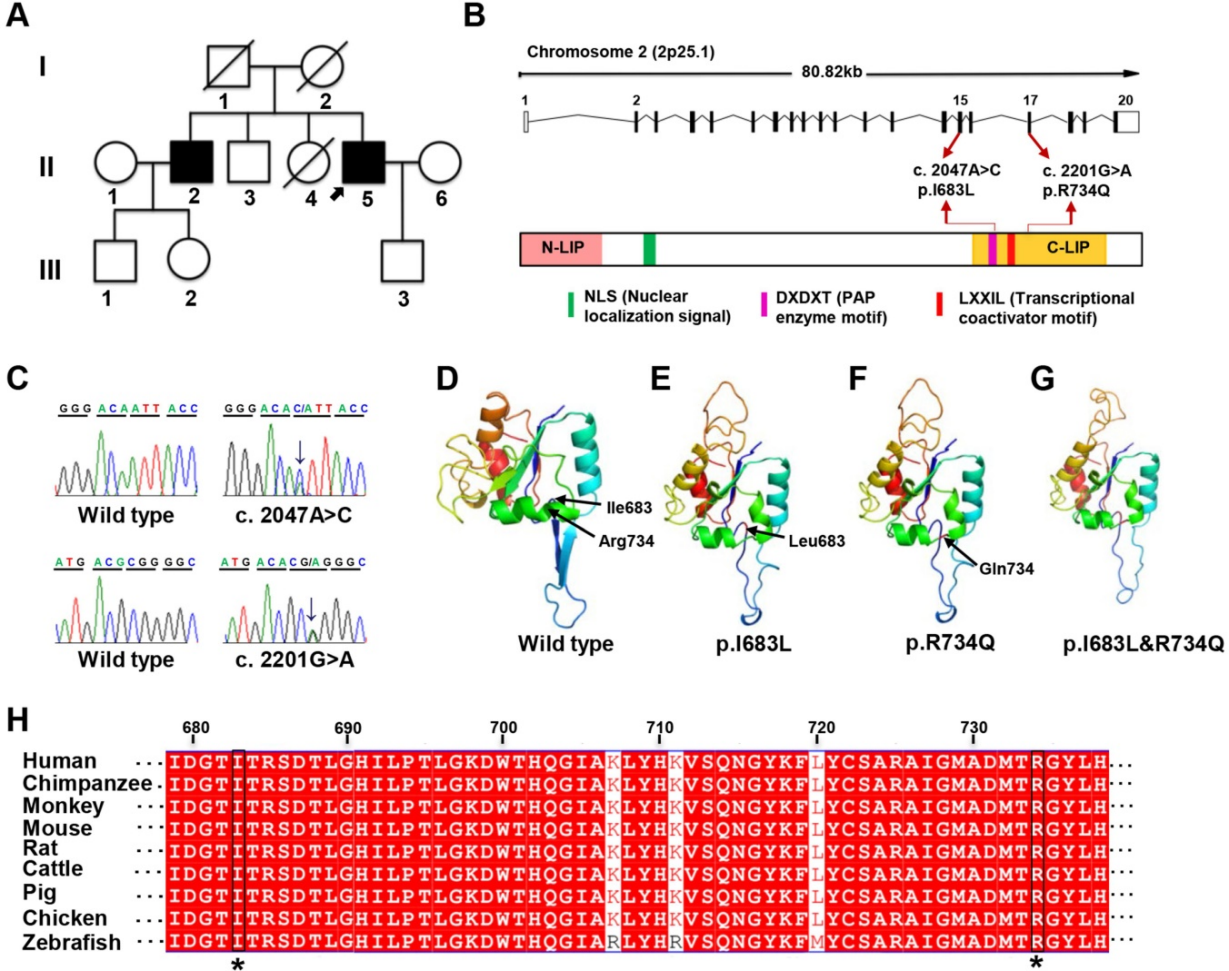
** Identification of LPIN1 mutations from a family presenting with adult-onset muscle weakness. (A)** Pedigree of the investigated family. The proband (II:5) was indicated by black arrow. **(B)** Schematic representation of the localization of identified LPIN1 mutations c.2047A>C (p.I683L) and c.2201G>A (p.R734Q) in the genome structure (top) and protein structure (bottom). **(C)** Sanger sequencing chromatographs showing two compound heterozygous mutations of c.2047A>C and c.2201G>A presenting in patients. **(D-G)** Crystal structures of human LPIN1 wild type and mutants carrying p.I683L or p.R734Q, or p.I683L/p.R734Q. **(H)** LPIN1was highly conserved across different vertebrate species; the conserved I683L and R734Q are highlighted with a star (*).

Whole-exome sequencing was subsequently performed on II2, II3, II4, and II5 to find disease-causative mutation in the family. A total of 817 small nucleotide variants (SNPs) and 414 indels were identified (Table S2-S4). Since the deceased parents of the proband did not present related symptoms, autosomal recessive inheritance was considered. Only 7 protein-changing variants including 6 genes were remained after further bioinformatics filtering (Table S5). Using a combination of segregation validation by direct Sanger sequencing and *in silico* analysis, two heterozygous mutations in *LPIN1* were identified in II2 and II5: (GRCh37 [hg19] chr2: g.11944690 A>C, GeneBank: NM_145693:c.2047A>C:p.I683L in exon 15 and GRCh37 [hg19] chr2: g. 11955273 G>A, GeneBank: NM_145693:c.2201G>A:p.R734Q in exon 17 (Figure 1A-C). Both of the two heterozygous mutations were predicted to be damaging and related to disease-causing by *in silico* prediction algorithms queried in dbNSFP (Table 2). The frequencies of two variants were extraordinarily low, only 2/121402 for c.2047A>C and 0/121220 for c.2201G>A in Exome Aggregation Consortium database, and were not found in the Chinese Millionome DataBase (Table 2). It was further confirmed by screening at least 100 unrelated healthy individuals.

The two missense mutations p.I683L and R734Q are located in C-terminal (C-LIP) domain of lipin 1 protein, which is crucial for PAP enzyme activity and transcriptional regulation (Figure 1B), and highly conserved across various species (Figure 1H). To predict the possible pathogenic effect of two missense mutations, LPIN1 wt, p.I683L, p.R734Q and p.I683L & p.R734Q were subjected to crystal structure modeling using SWISS-MODEL and PyMOL. In LPIN1 proteins with the mutations of p.I683L or p.R734Q, the β-sheets between Thr684 and Thr688, and between Gly697 and Thr701, of the wt protein were abolished; and the α-helices between Ile727 and Tyr736 were broken into two parts, rendering less compact protein structures (Figure 1D-G). Thus, it was likely that p.I683L and p.R734Q mutations affected the appropriate folding and biological functions of lipin 1 protein.

Mutations of* LPIN1* in humans have been identified as a major cause of recurrent, early-onset myoglobinuria and rhabdomyolysis (MIM#268200). Hitherto, 46 homozygous or compound heterozygous mutations in *LPIN1* gene from 48 cases have been identified in humans 14, 15, 35-46; among which 83.3% cause myoglobinuria, and 100% lead to rhabdomyolysis symptoms, mostly in childhood. About 41.7% and 33.3% of the *LPIN1* mutations result in myasthenia and myalgia respectively (Table 1). In addition to muscle phenotypes, the proband in this study manifested severe mixed peripheral neuropathy and nerve demyelination, which had not been reported in other *LPIN1* mutations where only neurogenic damage of skeletal muscle was found in one case 44 (Table 1). It suggested a critical role for LPIN1, not only in skeletal muscle, but also in the peripheral nervous system.

### Expression of zebrafish *lpin1* was specifically enriched in somites and neural tissues

**Figure 2:**
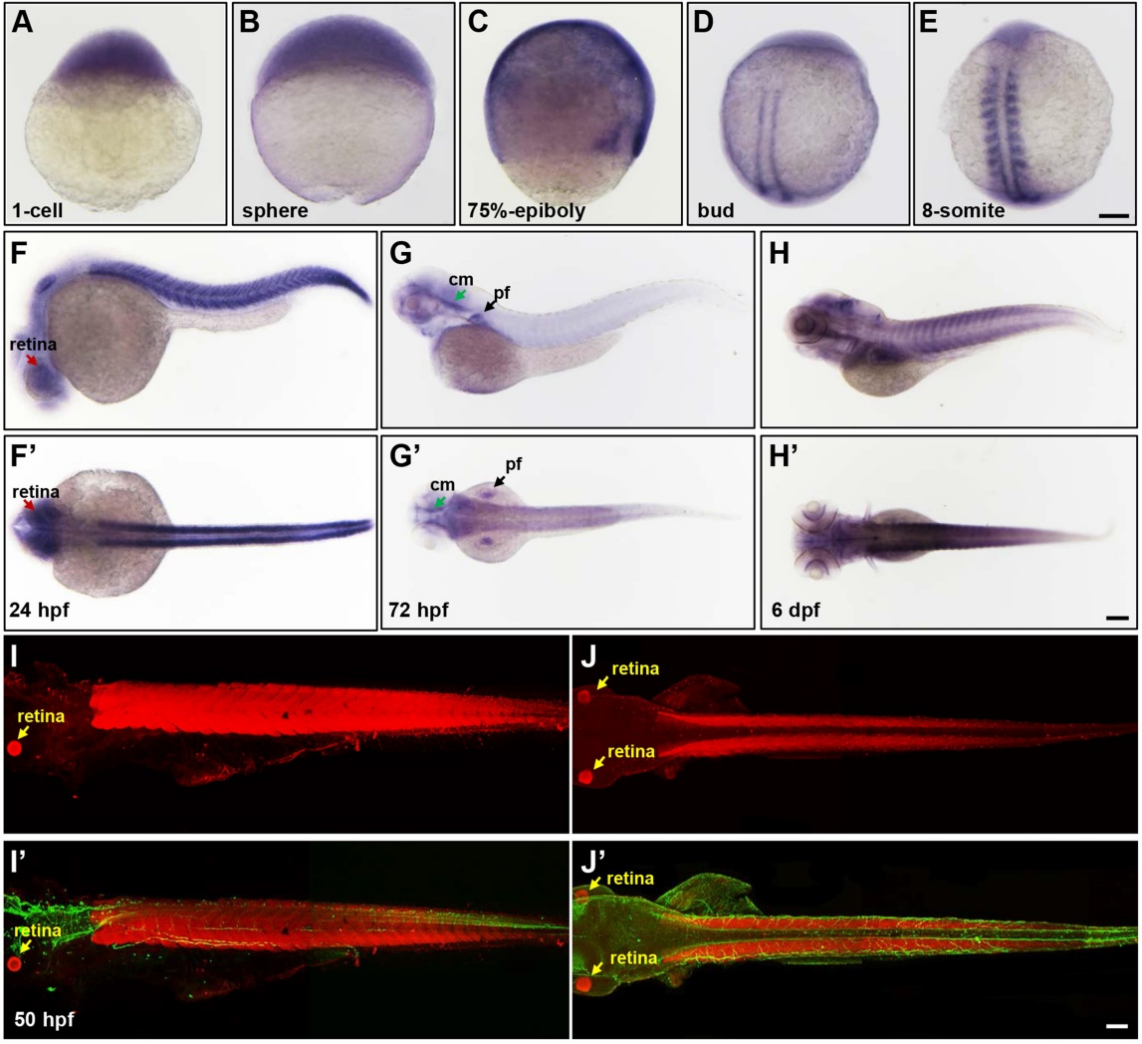
** The spatial and temporal expression patterns of *lpin1* during zebrafish early development.** The expression of *lpin1* mRNA was ubiquitous during cleavage and gastrula stages **(A-C)**, restricted to paraxial mesoderm at the bud stage **(D)**, and abundant at somites from the 8-somite stage onwards **(E-H')**. The dynamic *lpin1* mRNA expression was detected in the retina (red arrows in **F** and **F'**), pectoral fins (pf, black arrows in **G** and **G'**) and the cephalic musculature (cm, green arrows in **G** and **G'**). (I-J') IHC staining showed the Lpin1 protein expression in myotomes and the retina (yellow arrows), acetylated α-tubulin was stained in green color. (**F**-**J**) Lateral views of embryos, (**F'**- **J'**) Dorsal views of embryos. Scale bar: 100 µm.

To gain insight into the pathogenesis of *LPIN1* mutations in humans and LPIN1 functions *in vivo*, the zebrafish model was used in this study. The spatio-temporal expression patterns analyzed by whole-mount ISH analysis displayed a ubiquitous expression of *lpin1* mRNA from 1-cell to the blastula and gastrula stages (Figure 2A-C). At the end of gastrulation, *lpin1* mRNA was restricted to the bilateral stripes of presomitic mesoderm (Figure 2D). During somitogenesis, *lpin1* was expressed in the elongated adaxial cells and the striped paraxial mesoderm, and continued to be highly expressed in somites till a total of about 30 somite pairs were formed at 24 hour-post-fertilization (hpf) (Figure 2E-F'). The *lpin1* mRNA was also broadly expressed at the head region at 24 hpf, with a notable distribution in the retina. At 72 hpf, the *lpin1* mRNA was only detected at the neural crest, cephalic musculature and pectoral fins (Figure 2G, G'), but the myotome-specific expression was restored at 6 dpf (Figure 2H, H'). The levels of *lpin1* transcripts were confirmed by qRT-PCR (Figure S1). Consistent with the mRNA expression pattern of *lpin1*, IHC staining showed that the Lpin1 protein was specifically localized in myotomes as well as in the retina at 50 hpf (Figure 2I-J'). The enriched and specific expression of *lpin1* in developing somites and neural tissues of zebrafish implicated an essential role of *lpin1* in the formation and function of muscular and neural tissues.

### Lpin1 deficiency caused defects of skeletal muscle development in zebrafish

**Figure 3:**
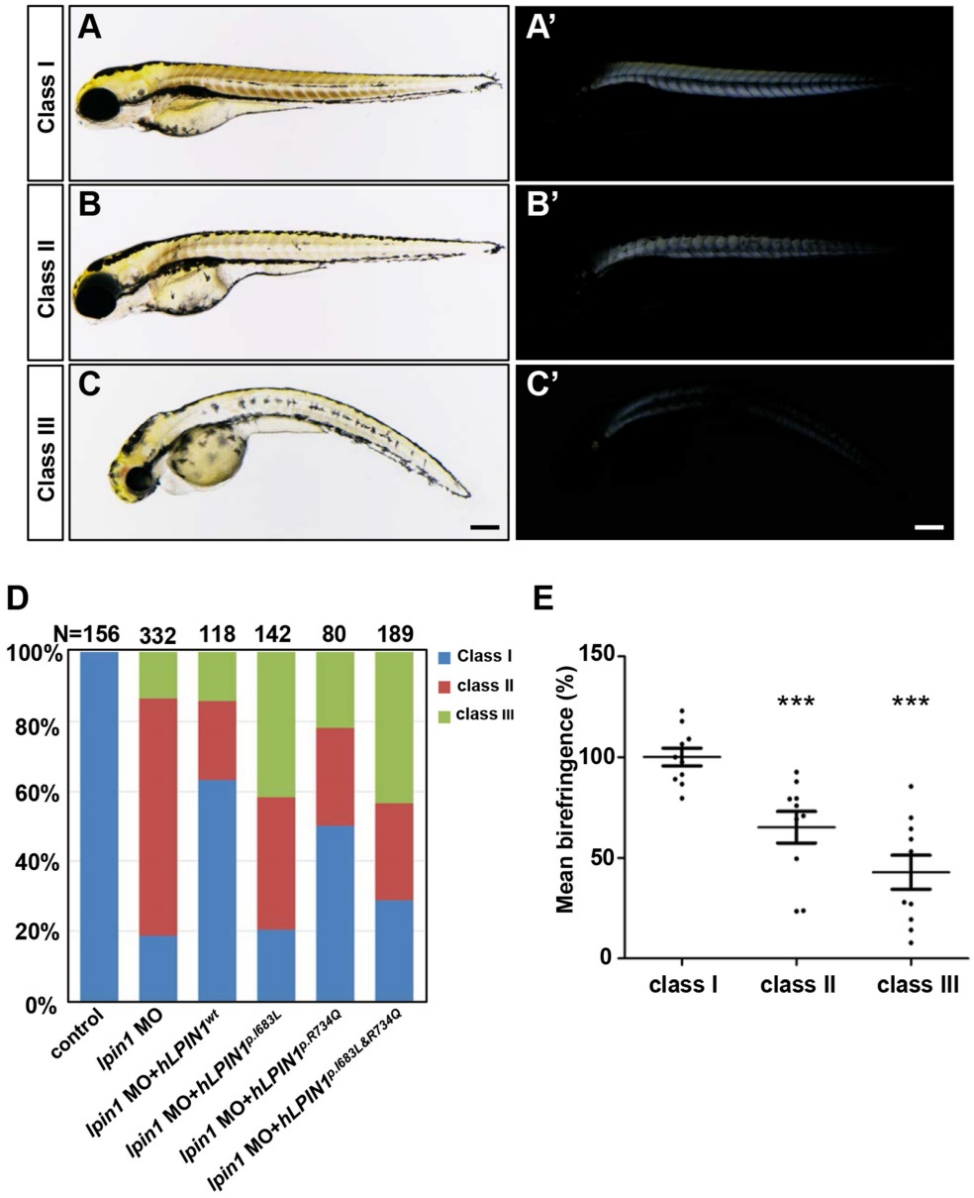
** Zebrafish* lpin1* morphant embryos showed defects in the skeletal muscle at 4 dpf. (A-C')** Classification of the *lpin1* morphants was based on the trunk phenotype **(A-C)** and the corresponding reduction in birefringence observed in skeletal muscles **(A'-C')**. Class I (A, A') represents the wild-type-like phenotype with neither defect in embryonic trunk nor reduced birefringence in the skeletal muscle. Scale bar: 200 μm. **(D)** The phenotype of *lpin1* morphants was rescued by the co-injected mRNA of human *LPIN1^wt^* but not *LPIN1^p.I683L^*, *LPIN1^p.R734Q^* or *LPIN1^p.I683L& R734Q^*. **(E)** Quantification data are calculated by the mean intensity of 10 somites (from somite 5 to 15) for each embryo using Image J, which is normalized to that of class I (control) to obtain percentage values. n = 10 embryos for each class. *** indicates* p* < 0.001 by Student's t-test.

Previous studies reported that LPIN1 deficiency resulted in peripheral neuropathy and skeletal muscle myocyte damage in mice 21, 47, 48. To further determine the biological function of lipin 1, we generated Lpin1-deficient zebrafish by *lpin1* MO injections or by overexpression of mutated human *LPIN1* mRNA. IHC analysis revealed that the Lpin1 protein expression in somites was dramatically decreased in *lpin1* MO injected embryos at 24 hpf, as compared with that in control embryos, indicating a successful knockdown effect of *lpin1* MO (Figure S2). *lpin1* morphant embryos were classified into three categories according to the severity of skeletal muscle phenotypes as revealed by birefringence imaging (Figure 3). In control embryos, the skeletal muscle was highly birefringent under polarized light due to the organization of myofibrils (Figure 3A, A'); but *lpin1* MO injected embryos displayed overall reduction in birefringence due to muscular lesions caused by myofibrillar disorganization in the major axial skeletal muscles (Figure 3B-C'). The quantification of the birefringence further revealed a significant reduction in the brightness in class II (35% reduction) and class III (58% reduction) of *lpin1* MO injected embryos (Figure 3E). Overexpression of mutant human *LPIN1^p.I683L^*, *LPIN1^p.R734Q^* or *LPIN1^p.I683L& R734Q^*, but not *LPIN1^wt^* mRNA caused a similar overall reduction in the birefringence of the skeletal muscle (Figure S3). Moreover, the *lpin1* MO induced skeletal muscle defects could be alleviated by the co-injected mRNA of human *LPIN1^wt^* but not *LPIN1^p.I683L^*, *LPIN1^p.R734Q^* or *LPIN1^p.I683L& R734Q^* (Figure 3D).

### Lpin1 deficiency led to defects of neuromuscular synapse formation in zebrafish

**Figure 4:**
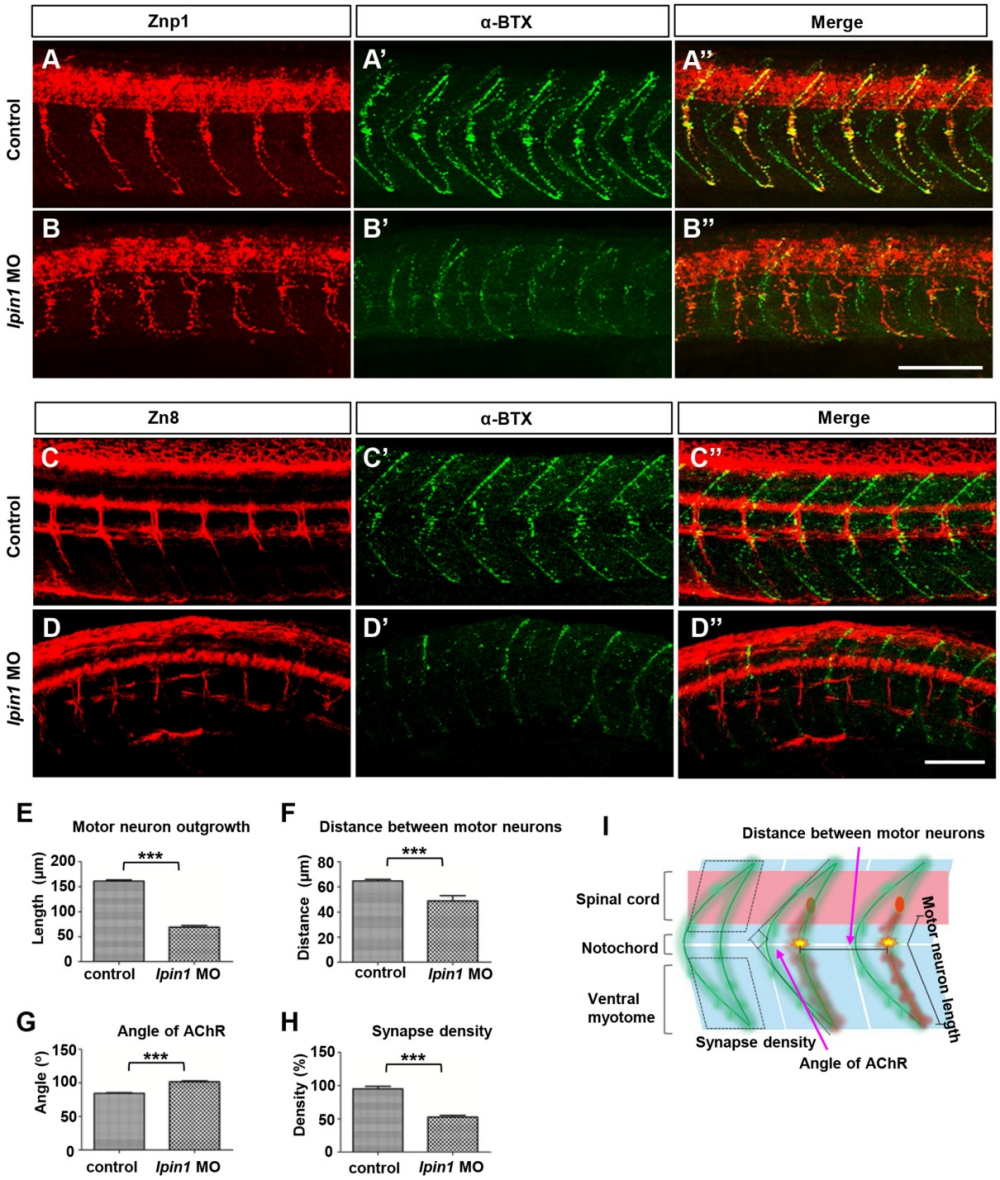
** Knockdown of *lpin1* in zebrafish embryos affected neuron development and AChR clustering.** Znp1 antibody staining **(A-B)** and α-BTX labeling **(A'-B')** of control embryos **(A-A'')** and *lpin1* morphants **(B-B'')** at 26 hpf. Zn8 antibody staining **(C-D)** and α-BTX labeling **(C'-D')** of control embryos **(C-C'')** and *lpin1* morphants **(D-D'')** at 60 hpf. Scale bar: 100 µm. Measurements of the length of motor neurons (**E,** n = 6 motor neurons/larvae), the distance between two adjacent motor neurons (**F,** n = 6 segments/larvae), the angle of AChR clusters (**G,** n = 6 AChR clusters/larvae), and the density of post-synapses (**H,** n = 6 areas of myotome/larvae), were carried out in 9 larva under each treatment. **(I)** Schematic representations of the parameters in E, F, G and H. *** indicates* p* < 0.001 by Student's t-test.

To address whether the loss of *lpin1* function in zebrafish affects the connection between nerve and muscle, we analyzed the projections of motor axons and the formation of neuromuscular synapses in *lpin1* MO injected embryos. Zebrafish have two populations of spinal motoneurons, PMNs and SMNs projections. In the control embryos at 26 hpf, PMNs were labeled by Znp1 antibody and exhibited a common axonal path, where motor neuron growth cones traversed the muscle territory into ventral and dorsal somitic muscle blocks 49 (Figure 4A). Meanwhile, AChR clusters (post-synapses) visualized by α-bungrotoxin (α-BTX) 49, 50 were formed properly and correlated well with motor axonal projections (Figure 4A', A''). However, *lpin1* MO injected embryos displayed a significant reduction in both motor neuron axonal projections and AChR clusters (Figure 4B-B''). The length of the PMNs was significantly shorter in the *lpin1* morphant (69.2 ± 19.3 μm, n = 9) than in the control embryo (161.0 ± 16.6 μm, n = 9) with a ~69% reduction (Figure 4E, I). The distance between motor axons in control embryos (64.6 ± 7.6 μm) was also reduced in the *lpin1* morphant (49.1 ± 25 μm) (Figure 4F, I). The morphological changes of AChR clusters were measured by calculating the angle of AChR stripes with a ~20% increase in the *lpin1* morphant (102 ± 8.8^ o^) compared to the control embryo (84.4 ± 6.6^ o^) (Figure 4G, I). Quantification of the post-synaptic densities showed that *lpin1* morphants had lower density with a ~44% decrease compared to controls (Figure 4H, I). In addition, the co-localization of PMNs and post-synapses was disorganized in *lpin1* morphants, indicating that the motor axons cannot innervate the muscle fiber properly.

After the localization of PMNs was completed, at ~25 hpf, SMNs extend their axons along the paths pioneered by primary axons. Axons of both PMNs and SMNs eventually form the dorsal and ventral motor nerves 51. Once the development of PMNs is ablated, the extension of SMNs to their targets will be delayed 52. In the control embryos at 60 hpf, it was observed that SMNs labeled by Zn8 completed their axonal migration along the common paths and the axons of the ventral nerve extended to the ventral myotome (Figure 4C), and AChR clusters also exhibited a universally and regular distribution (Figure 4C', C''). In *lpin1* morphants, axons of SMNs had very weak signal intensity, showed an additional decrease in the length of motor neurons and an additional increase in premature branching, failing to reach the ventral myotome (Figure 4D). The density of AChR clusters was greatly reduced in *lpin1* morphants (Figure 4D', D''). At 5 dpf, the α-BTX signals in *lpin1* morphants were reduced significantly in both fast muscles and slow muscles compared to that in control embryos (Figure S6A, B) 53. qRT-PCR results also confirmed the expression levels of almost all nicotinic acetylcholine receptors were decreased in *lpin1* morphants (Figure S6G). The malformation of PMNs and SMNs axonal projections, and AChR clusters were also observed in those embryos injected with the mRNA of human *LPIN1^p.I683L^*, *LPIN1^p.R734Q^* or *LPIN1^p.I683L& R734Q^*; but not in *LPIN1^wt^* injected embryos (Figure S4, S5, S6). Therefore, the common axonal paths as well as the neuromuscular junction were both affected in Lpin1-deficent zebrafish embryos.

### Lpin1 deficiency resulted in impaired myelination in zebrafish

**Figure 5:**
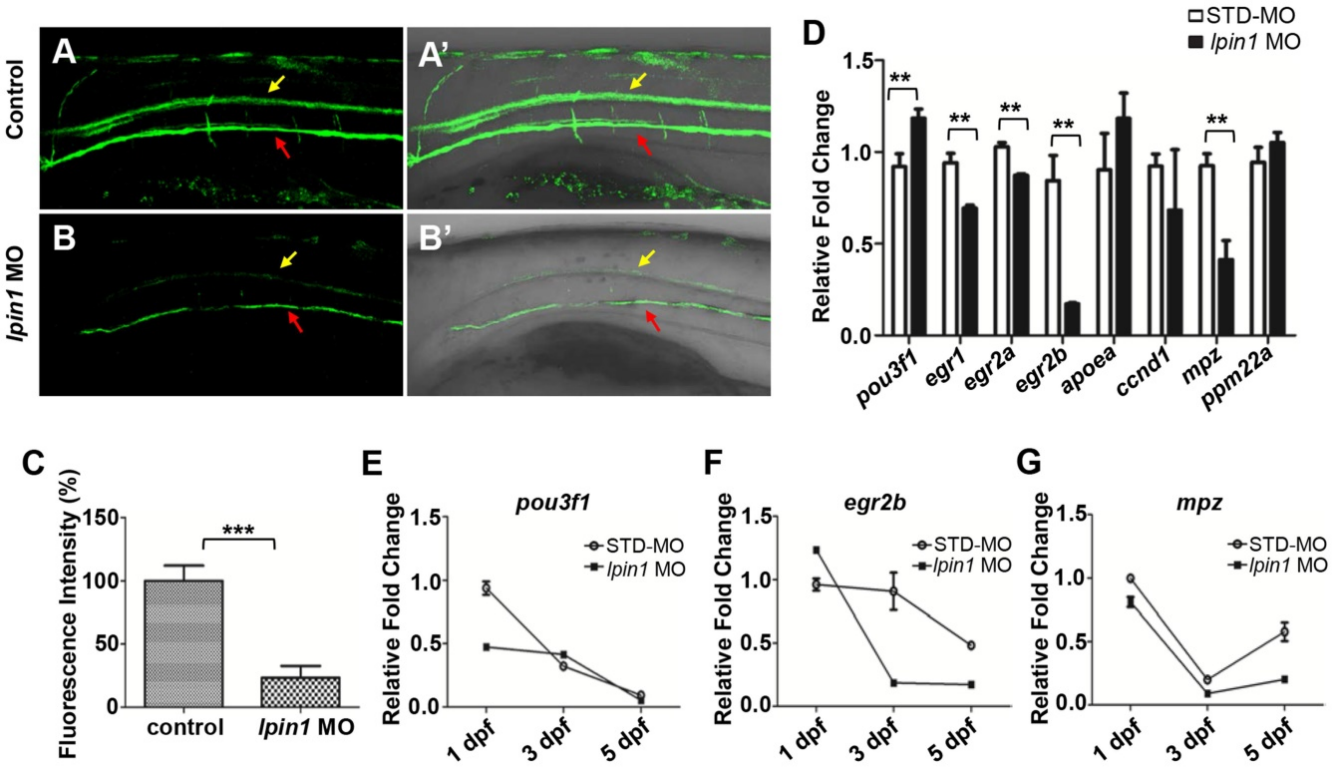
***Lpin 1* deficiency impaired the maturation of schwann cells and myelin synthesis. (A-B')** GFP signals of 5 dpf *Tg(mbp:eGFP)* larvae was visualized by fluorescent microscope, yellow arrows indicated GFP signal in the spinal cord, and red arrows indicated GFP signal motor neurons, lateral views, scale bar: 100 μm.** (C)** Mbp fluorescence signals of control embryos and *lpin1* morphants were analyzed by Image J software. Ten independent samples were evaluated. **(D)** qRT-PCR measurements of immature SC markers, mature SC markers, and myelin gene markers in *lpin1* morphants and STD-MO injected embryos at 3dpf. **(E-G)** qRT-PCR measurements of *pou3f1*, *egr2b*, and *mpz* dynamic expression at 1 dpf, 3 dpf, and 5 dpf respectively. Values in **(D-G)** represented means ± SE of data from three independent experiments, **p< 0.01, and ***p < 0.001 (Student's t-test).

Myelin sheath surrounds nerve axons, allowing saltatory nerve conduction and maintenance of the axon at a long distance from the cell body 54. Myelin basic protein (MBP) is produced by differentiated oligodendrocyte lineage cells, including oligodendrocytes in the central nervous system and Schwann cells in the peripheral nervous system 55. In zebrafish, *Tg(mbp:eGFP)* transgenic strain facilitates the visualization of myelination 25. At 5 dpf, the GFP signals expressed in oligodendrocytes and SCs were dramatically decreased in *lpin1* morphants compared to control larvae (Figure 5A-B').The quantification of mbp fluorescence signals was also reduced by 70% in *lpin1* morphants compared with control larvae (Figure 5C). It suggested that the loss of *lpin1* function in zebrafish larvae caused impaired myelination, consistent with our patient phenotype, which neurohistological examination of left sural showed a significant decrease in myelinated nerve fibers and severe nerve demyelination. *Tg(mbp:eGFP)* embryos injected with the mRNA of human *LPIN1^ p.I683L^*, *LPIN1^ p.R734Q^* or *LPIN^1 p.I683L& R734Q^*, but not *LPIN^ wt^* were also observed impaired myelination (Figure S7).

In order to quantitate the myelination defects in *lpin1* morphants at the molecular level, we examined the expression of genes involved in myelination. qRT-PCR showed that the expression of immature SCs marker *pou3f1* 57 was abnormally increased, while the expressions of mature SCs markers, *egr1* 58, *egr2a* 59, *egr2b* 60 and myelin marker, *mpz* 61 were abornarmally decreased in *lpin1* morphants at 3 dpf (Figure 5D). The dynamic expressions of these genes further revealed that the expression of *pou3f1* was higher in *lpin1* morphants at 3 dpf and was followed by decreased expression of *egr2b* and *mpz* at 3dpf and 5dpf. This indicated that SCs were kept in an immature state, resulting in a decrease in myelin synthesis in Lpin1-deficient zebrafish embryos (Figure 5E-G). Altogether, these findings demonstrated that *lpin1* might mediate oligodendrocytes and Schwann cells differentiation, which was crucial for the normal progression of myelination in zebrafish.

### Lpin1-deficient zebrafish embryos exhibited abnormalities of swimming behaviors

**Figure 6:**
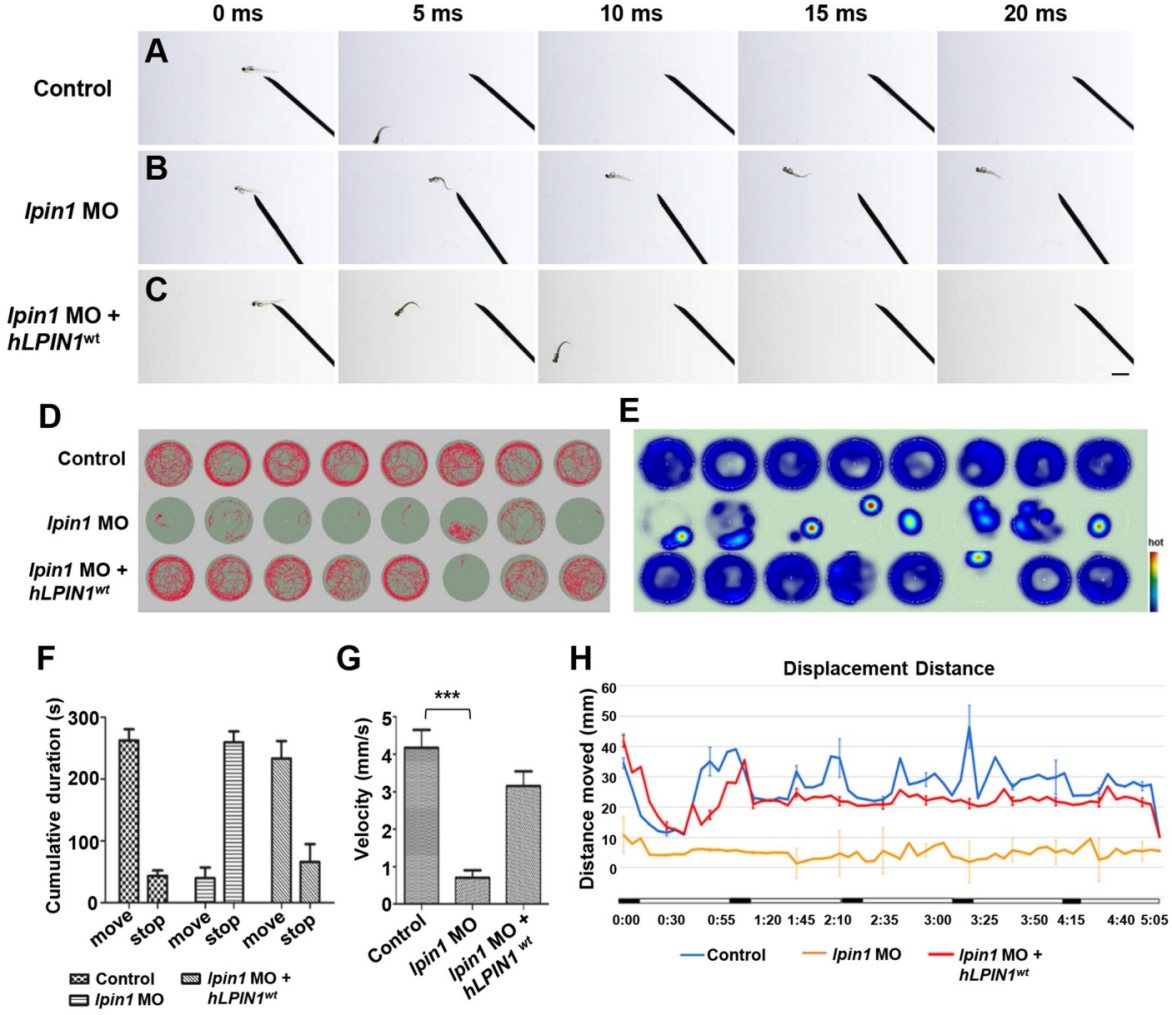
** Locomotor deficits in *lpin1* morphants. (A-C)** *lpin1* morphant embryos exhibited reduced touch-evoked response at 4 dpf **(B)** while control embryos swam away rapidly after mechanosensory stimulation **(A)**. The reduced touch-evoked response of *lpin1* morphant could be rescued by co-injected *hLPIN1^wt^* mRNA** (C)**. Scale bar: 200 µm. **(D)** Cumulative plots of the position and velocity of control larva, *lpin1* morphants, and *lpin1* morphants co-injected with* hLPIN1^wt^* mRNA; during 5 min of behavioral recording. 8 representative individuals were recorded for each treatment group. **(E)** Representative heat map plots. Red dots indicate the long rest time without any movement. **(F)** Cumulative duration records showed that the *lpin1* morphants were less active. **(G)** Velocity was dramatically decreased in *lpin1* morphants, but can be rescued by the *hLPIN1^wt^* mRNA. **(H)** Total movement distance during a 5 min startle response analysis as follows: 20 s spontaneous movement tracking followed by cycle stimulation with light on 5 s, interval 1 s, trigger tap 1 s, and interval 48 s; repeated 5 cycles. Data shown are means of distance moved in 5 s ± SE (n = 24 for each group at 5 dpf).

The severe defects in the skeletal muscle, neuromuscular synapses and myelination of Lpin1-deficient embryos directly affected the swimming behaviors of zebrafish larva. In contrast to the 4 dpf control larva that swam away rapidly after 5 milliseconds (ms) in response to touch (Figure 6A), *lpin1* morphants showed an impaired response to touch and failed to swim away from the field of view even after 20 ms (Figure 6B). This touch-evoke failure could be partially rescued by human *LPIN1^wt^* mRNA (Figure 6C).

To further examine the effect of Lpin1 deficiency on swimming activity, we quantified the velocity and the movement distance of individual zebrafish larvae at 5 dpf by locomotion assay. The movement trajectory and the distance of zebrafish larva were generated over a 5 minutes (min) period. Individual control larvae showed high activity and mobility by measuring the travel trajectory and movement heat map at an interval of 0.5 s (Figure 6D, E). On the other hand, *lpin1* MO morphants displayed severely reduced activity and mobility over the test period. The co-injection of *lpin1* MO and human *LPIN1^wt^* mRNA rescued the defective locomotion phenotype (Figure 6D, E). The cumulative duration records during a period of 300 s indicated that *lpin1* morphants had a dramatically reduced movement time (39.9 ± 49.2 s) and increased rest time (259.9 ± 49.1 s) compared to those of control larvae (movement time: 262.8 ± 50.3 s; rest time: 43.5 ± 24.8 s) and *hLPIN1^wt^* mRNA co-injected larvae (movement time: 233.5 ± 24.8 s; rest time: 66.3 ± 30.5 s) (Figure 6F, n = 24 for each group). Correspondingly, the velocity of *lpin1* morphants was also significantly reduced (0.71 ± 0.56 mm/s) compared to the control larvae (4.17 ± 1.34 mm/s) and *hLPIN1^wt^* mRNA co-injected larvae (3.15 ± 1.13 mm/s) (Figure 6G, n = 24 for each group). The total distance moved was quantified over a 5-min period for individual zebrafish larvae. *lpin1* morphants displayed a significant decrease in the mean distance swum (309.9 ± 4.9 mm) compared to the control larvae (1686.5 ± 26.8 mm) and rescued larvae (1402.9 ± 22.3 mm) (Figure 6H, n = 24 for each group). The locomotion assay was further applied to the 5 dpf zebrafish larvae overexpressing human *LPIN1^wt^*, *LPIN1^p.I683L^*, *LPIN1^p.R734Q^* or *LPIN1^p.I683L& R734Q^* respectively. Similar to the swimming behavior of *lpin1* morphants, the zebrafish larvae injected with the mRNA of *LPIN1^p.I683L^*, *LPIN1^p.R734Q^* or *LPIN1^p.I683L& R734Q^* exhibited dramatically decreased movement velocities and distances compared to either control or *LPIN1^wt^* overexpressing larva (Figure S8). The reduced mobility caused by the *lpin1* MO or the overexpressed mutant *LPIN1* mRNA in larval zebrafish was consistent with the defective structures of myotome and NMJs; and partially phenocopied the abnormalities of human patients with mutations in *LPIN1*. Together, these results suggested a conserved and important role of LPIN1 in the function of neuromuscular tissues.

### Lpin1 knockdown in zebrafish and mammalian cells caused abnormal expression of muscle and neuron markers

**Figure 7:**
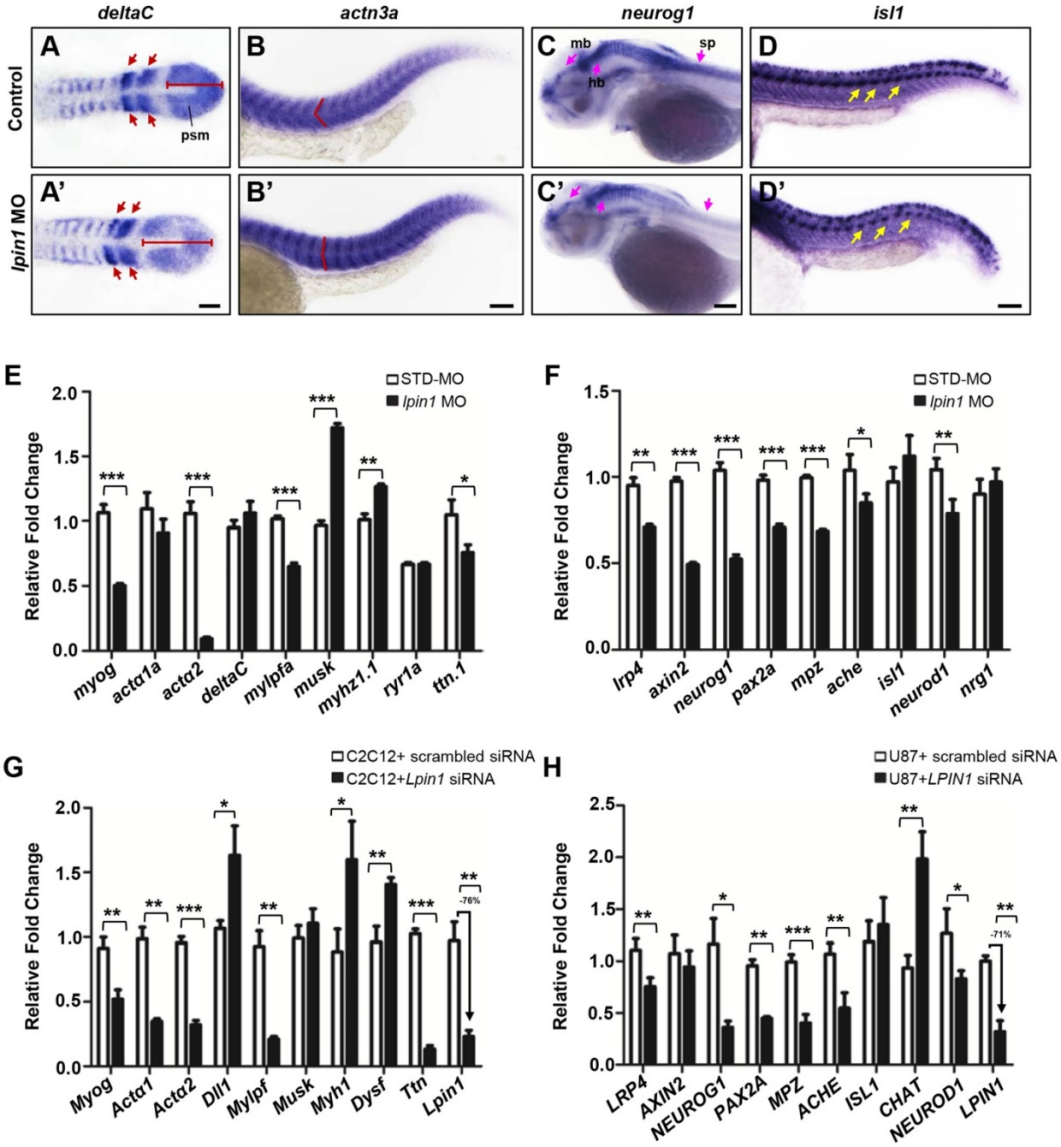
***lpin1* knockdown affects gene expressions in the muscle and neuron development of zebrafish larva.** The expression of somite marker *deltaC* in control embryos **(A)** and *lpin1* morphant **(A')** embryos at 8-somite stage. Red arrows indicate newly formed somites and red bars indicate the length of presomitic mesoderm. Control embryos **(B)** and *lpin1* morphant **(B')** embryos at 25 hpf were stained for *actn3a*. Note the shape of myotome, indicated by red angle, was changed in *lpin1* morphants. The expression of neuron markers *neurog1* **(C, C')** at 50 hpf and *isl1* **(D, D')** at 25 hpf in control embryos **(C, D)** and *lpin1* morphants **(C', D')**. In *lpin1* morphants, the expression of *neurog1* was reduced in the midbrain (mb), hindbrain (hb), and spinal cord (sp) (pink arrows); and that of *islet1* was reduced in PMNs (yellow arrows). Scale bar: 100 μm. The expressions of muscle **(E)** and neuron **(F)** markers, as determined by qRT-PCR, were significantly changed in *lpin1* morphants at 4 dpf and 1 dpf respectively. *Lpin1* siRNA knockdown in C2C12 **(G)** and U87 cells **(H)** resulted in abnormal expressions of muscle and neuron markers. Values in (E, F, G and H) represent means ± SE of data from three independent experiments, *p< 0.05, **p< 0.01, and ***p< 0.001 (Student's t-test).

According to the dramatically decreased locomotion ability observed in* lpin1* morphants, we further assessed several markers of muscle and neuron development including* deltaC* 61, *deltaD* 61, segmental plate and somite markers; *actn3a* 62, *musk* 63, expressed in muscle fibers; *neurog1* 64, *lrp4* 29, expressed in the central nervous system; and *isl1* 65, a spinal cord motor neuron marker. At the 8-somite stage, the expression of *deltaC* and *deltaD* in *lpin1* morphants was expanded in the presomitic mesoderm and abnormally distributed in newly formed somites, as compared to control embryos (Figure 7A, A', Figure S9A, A'). The expression of *actn3a*, a muscle-specific actin highly and specifically expressed in myotome at 25 hpf, indicated a morphological change in the myotome shape and arrangement in *lpin1* morphants (Figure 7B, B'), similar change was observed using *musk* marker (Figure S9B, B'). The expression of *neurog1* in midbrain, hindbrain, diencephalon and spinal cord of the *lpin1* morphants at 50 hpf was obviously decreased compared to control embryo (Figure 7C, C'), suggesting a defective neurogenesis. The high expression in rhombic lip and pectoral fin of *lrp4* was also reduced in *lpin1* morphants at 50 hpf (Figure S9C, C'). The shape and location of PMNs in the spinal cord, as labeled by *isl1* expression, were altered in 25 hpf *lpin1* morphants compared to control embryos (Figure 7D, D'). In addition, the expression levels of muscle and neuron markers were significantly altered in *lpin1* morphants as detected by qRT-PCR analysis (Figure 7E, F). Thus, both qualitative and quantitative analysis of gene expressions in zebrafish support that *lpin1* was required for muscular and neuronal development.

In order to address the muscular and neuronal anomalies due to lipin 1 deficiency, we further modeled the loss of *LPIN1* by siRNA knockdown in mammalian cells. In mouse myoblast C2C12 and human primary glioblastoma U87 cells, *LPIN1* siRNA achieved > 70% knockdown efficiency, resulting in abnormal expression levels of muscular and neuronal markers (Figure 7G, H). Therefore, the results of quantitative analysis of gene expressions in LPIN1*-*deficient mammalian cells were consistent with the Lpin1-deficient phenotypes in zebrafish, suggesting a conserved and essential role for LPIN1 in the regulation of muscular and neuronal gene expressions.

### Loss of lipin 1 function led to increased Notch signaling in zebrafish and mammalian cells

**Figure 8:**
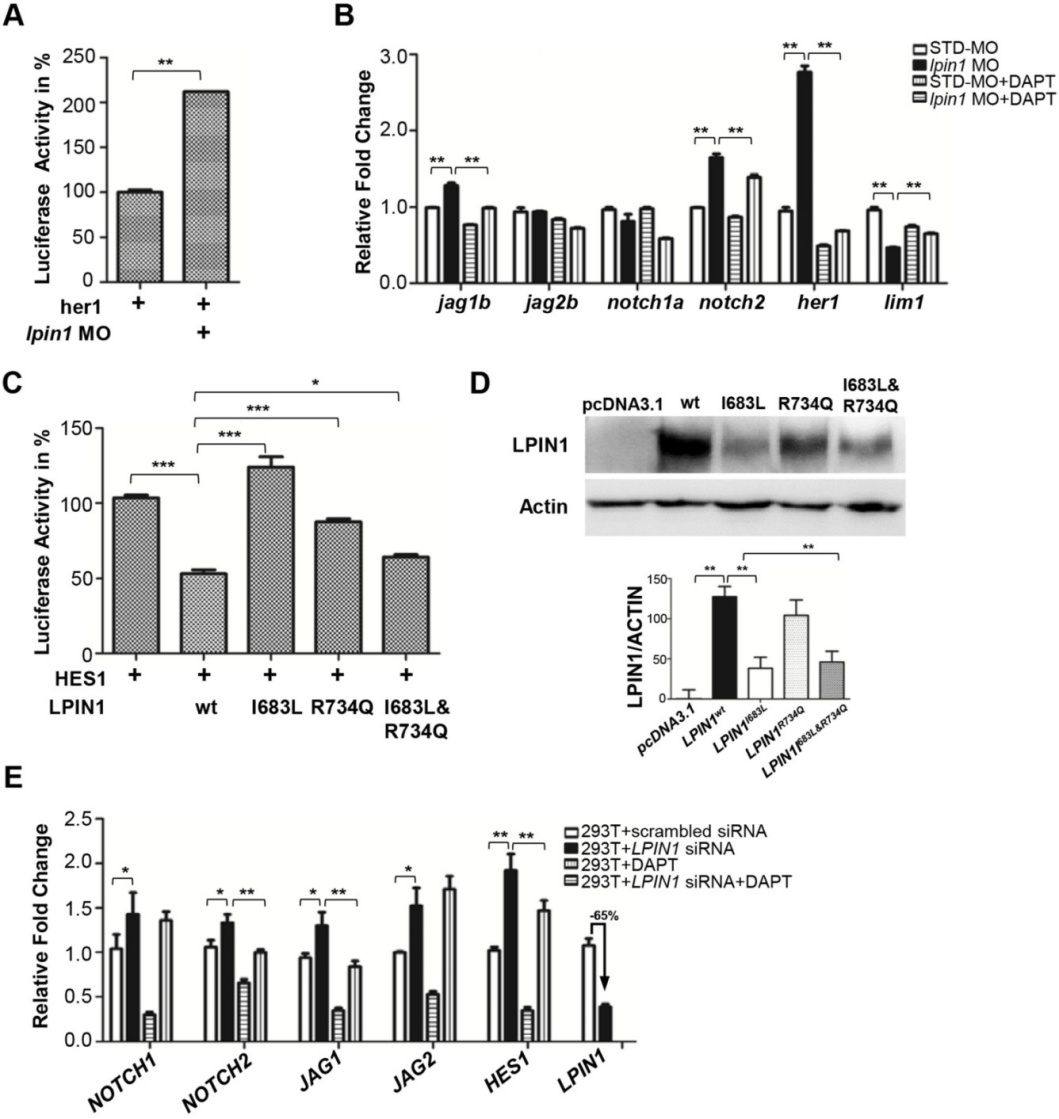
** Loss of lipin 1 function results in activation of Notch signaling in zebrafish and mammalian cells. (A)** Dual luciferase reporter assay to quantify the Notch signaling activity as *her1* was coinjected with *lpin1* morpholino into zebrafish embryos. **(B)** Lpin1-deficient zebrafish embryos showed upregulated gene expressions of Notch signaling components, which can be partially rescued by Notch inhibitor DAPT. **(C)** Dual luciferase reporter assay to quantify the Notch signaling activity as HES1 was coexpressed with the indicated *LPIN1* cDNA in HEK293T cells. **(D)** Western blot and quantification analysis of LPIN1 wild type and mutant proteins. **(E)** *LPIN1* siRNA in HEK293T cells achieved 65% knockdown with upregulated gene expressions of NOTCH signaling components. The abnormal upregulation of NOTCH signaling can be rescued by NOTCH inhibitor DAPT. Values in **(A-E)** represent means ± SE of data from three independent experiments, *p< 0.05, **p< 0.01, and ***p< 0.001 (Student's t-test).

In zebrafish, the highly specific expression of *lpin1* mRNA and protein at somites, and the dysfunction of myogenesis and neurogenesis due to the loss of Lpin1 function, are reminiscent of the gene expressions and phenotypes regulated by Notch signaling 66, 67. Therefore, we assessed whether the Notch pathway was affected by the loss of lipin 1 function in zebrafish. Dual-luciferase Notch reporter assay performed in zebrafish indicated that the Notch pathway activity was upregulated in Lpin1*-*deficient embryos (Figure 8A). qRT-PCR analysis further confirmed that the relative transcript abundance of *jag1b*, a Notch ligand; *notch2*, a Notch receptor; and *her1*, an effector of Notch; were significantly upregulated in the *lpin1* morphant. In contrast, the expression of *lim1* known to be repressed by Notch signaling was decreased markedly. The addition of DAPT, a Notch signaling inhibitor, could partially rescue the over-activation of Notch pathway in Lpin1*-*deficient embryos (Figure 8B). These results suggested that the Notch activity was negatively regulated by Lpin1 in zebrafish.

To investigate whether the two identified mutations in patients lead to a loss of LPIN1 function in Notch pathway, we analyzed the effect of I683L and R734Q on the activation of NOTCH signaling by Dual-luciferase reporter assay in transiently transfected HEK293T cells. Consistent with the findings in zebrafish, LPIN1 was able to significantly inhibit NOTCH signaling as measured by HES1 reporter. In contrast, the mutated LPIN1 harboring I683L, or R734Q, or both I683L and R734Q abolished the antagonistic effect of LPIN1 on the NOTCH signaling activity (Figure 8C). Western blot analysis further revealed that the protein abundance of LPIN1^p.I683L^, LPIN1^p.R734Q^ and LPIN1^p.I683L& R734Q^ were lower than that of LPIN1^wt^ protein, implicating compromised stability of mutant LPIN1 proteins (Figure 8D).

To further substantiate the influence of LPIN1 on NOTCH signaling, we carried out siRNA knockdowns of LPIN1 in HEK293T, human primary glioblastoma U87, mouse myoblast C2C12, and human osteosarcoma U2OS cells, respectively. In HEK293T cells where *LPIN1* siRNA achieved > 65% knockdown efficiency, the levels of *NOTCH1*, *NOTCH2*, *JAG1*, *JAG2* and HES1were increased significantly. The abnormal augmentation of NOTCH pathway components could be rescued by the addition of DAPT (Figure 8E). Similarly, the siRNA knockdown of LPIN1 in U87, C2C12, and U2OS cells also led to an abnormal increase of NOTCH signaling at different levels (Figure S10). Taken together, these results from zebrafish and mammalian cell lines showed that LPIN1 possessed widespread repressive activity on NOTCH signaling, which might be a possible pathomechanism for the muscle and neuron phenotypes induced by lipin 1 deficiency in human and zebrafish.

## Discussion

Since Zeharia *et.al* 15 identified six mutations of the *LPIN1* gene in 25 patients aged 2-7 years with recurrent myoglobinuria for the first time, up to now there are 48 *LPIN1* mutation sites reported in human. More than 90% recessive *LPIN1* mutations, including homozygous or compound heterozygous genotypes, cause myoglobinuria and rhabdomyolysis of which more than 80% of patients developed in early childhood (<5 years old). Although the majority of human LPIN1 mutations manifested myoglobinuria, no obvious myoglobinuria phenotype was found in the *Lpin1* mutant mice 19, 20, 68. LPIN1-deficient mice exhibited defects not only in lipid metabolism, but also in muscle and neuron development. For example, the first reported* Lpin1* mutant mice *Lpin1^fld/fld^* was identified by neonatal fatty liver, lipodystrophy, insulin resistance, and peripheral neuropathy 18, 19. Nadra *et. al.* 9 verified the function of Lpin1 for myelin formation and maintenance through the analysis of *Lpin1^fld/fld^* mice. Sellers *et. al.* 20 also reported the phenotypic changes in skeletal muscle fibers of *Lpin1^fld/fld^* mice. Recently, Stepien *et. al.* 44 reported a case of 25-year-old Irish woman who carried compound heterozygous mutations of *LPIN1* (c.2295-866_2410-30del1763; c.942C>A). The patient was reported to have persistent myalgia and bilateral common peroneal neuropathies. This is the first description of a human patient with nerve and muscle phenotypes caused by LPIN1 mutation. However, the patient with LPIN1 deficiency also had an episode of rhabdomyolysis requiring intensive care admission. Here, we were first to report two novel compound heterozygous mutations, c. 2047A>C (p.I683L) and c. 2201G>A (p.R734Q) in LPIN1, which caused adult-onset syndromic myasthenia. The clinical diagnosis of the proband revealed the abnormality of skeletal muscle fibers and neurogenic damage with severe nerve demyelination. The progressive muscle weakness appeared in adulthood without any episode of myoglobinuria. Previously, Jama *et. al.* 48 generated conditional knockout mice* Lpin1^Myf5cKO^* and found that LPIN1 deficiency induced reduced muscle mass and myopathy. In addition, *HSA-Cre;Lipin1^floxed/floxed^* mice, where a skeletal muscle-specific lipin 1 deficiency was achieved, exhibited a severe sarcoplasmic reticulum stress, necrosis of skeletal muscle fibers, stress disorder of gastrocnemius muscle, and triggered myopathy 68. The phenotypes of skeletal muscle-specific lipin 1 deficiency in mice were consistent with the clinical features of our patient, which included predominance of type I muscle fibers, atrophy of type II muscle fibers, morphological abnormality of muscle fiber mitochondria, and progressive myopathy. On the other hand, by inactivating of *Lpin1* gene in Schwann cells, *MPZ^Cre/+^/Lp^ fE2-3 / fE2-3^* mice showed demyelination of nerve cells, dedifferentiation of Schwann cells, and reduced nerve conduction velocity 9. Consistently, the neurohistological examination of left sural in our patient showed a significant decrease in myelinated nerve fibers and severe nerve demyelination, which was similar to the neural phenotypes observed in LPIN1-deficient mice. Furthermore, Lpin1-deficiency in the zebrafish larvae induced by either *lpin1* MO knockdown or overexpression of human point mutations caused myofibrillar disorganization in the axial skeletal muscles, abnormal formation of PMNs, SMNs and post-synaptic clusters, and myelination defects; which in turn led to serve defects in locomotion ability and swimming behaviors (Figure 3,4,5,6,S4, S5, S6, S7 and S8). The phenotypic analysis of lipin 1 deficiency in previous studies using mice, and in our study of human mutations and the zebrafish model, together supported a highly conserved role for lipin 1 in the development and function of neuromuscular tissues.

Notch signaling is well-known to play critical roles in the regulation of somitogenesis and post-natal myogenesis. During early zebrafish embryogenesis, a number of Notch pathway components including *deltaC*, *deltaD*, *notch1a*, *notch6*, *her1*, *her4* are expressed in the presomitic mesoderm, paraxial mesoderm and corresponding somites, which are important to the maintenance of segmental clock and establishment of the somite polarity 69-71. ISH analysis showed that* lpin1 mRNA* was expressed in the presomitic mesoderm at the tailbud stage, and in the adaxial cells and segmental somites during somitogenesis, similar to the expression pattern of myoD, a member of the bHLH transcription factors acting sequentially in myogenic differentiation 72. Antibody staining further revealed a highly specific expression of Lpin1 protein in the myotome (Figure 2). The expression patterns and deficiency phenotypes of *lpin1* in zebrafish were reminiscent of those regulated by Notch signaling during somitogenesis and myogenesis. Consistently, *deltaC* and *deltaD* expressions in *lpin1* morphants were expanded at the presomitic mesoderm and abnormally distributed in newly formed somites, as compared to control embryos (Figure 7, S9); thereby suggesting an alteration of Notch signaling during somite formation. The *her1* reporter activity assay and the notch inhibitor treatment led to the discovery that Lpin1 acted as an antagonist of Notch signaling pathway during zebrafish embryogenesis. Further analysis in human and mouse cells confirmed the negative regulation of Notch pathway by LPIN1. HES1 reporter assay in HEK293T cells revealed that the repression of Notch activity by LPIN1 was partially abrogated by I683L and R734Q mutations. Strikingly, LPIN1 deficiency facilitated by siRNA knockdown in human and mouse cells caused abnormally upregulated Notch pathway, which can be partially rescued by the Notch inhibitor DAPT (Figure 8). Therefore, the experiments in both zebrafish and mammalian cells confirmed the conserved inhibitory functions of LPIN1 upon Notch signaling activity during development.

Another interesting phenotype observed in Lpin1-deficient zebrafish embryos was the defects in motor neuron outgrowth and synapse formation. Analysis of PMNs and SMNs in Lpin1-deficient embryos revealed impaired axonal outgrowth, as well as an increased arborization of the PMNs and SMNs that altered the normal ventral pathfinding. The morphological changes and mis-patterning of post-synapses were also observed in Lpin1-deficient embryos. Furthermore, the co-localization of primary motor axons and post-synapses was disorganized, indicating that the NMJ was deformed and dysfunctional in* lpin1* morphants. NMJ is the synapse formed between motor neurons and skeletal muscle fibers, and is covered by SCs in vertebrates 73, 74. NMJ-related diseases include congenital myasthenic syndromes, myasthenia gravis, and Lambert-Eaton myasthemic syndrome, etc. 75. Other neuromuscular diseases also include amyotrophic lateral sclerosis, muscular dystrophy, and different kinds of muscle weakness. AGRIN-LRP4-MuSk is a well-known signaling complex during NMJ formation. In zebrafish mutant *unplugged*, the homologue of muscle specific tyrosine kinase (MuSK), AChR clustering could be initiated but the approaching of growth cones to the muscle center is restricted, which is similar to the phenotype observed in Lpin1-deficient zebrafish embryos 76, 77. The expression patterns of *lpin1* is also very similar to that of *unplugged*; both of genes are highly expressed in somites during somitegenesis and in myotomes at 24 hpf, subsequently the expressions are restricted to pectoral fins and cephalic musculature at later stages. In *lpin1* morphants, the expression patterns and levels of *musk* and *lrp4* were altered as well. These evidences implicate that LPIN1 may play a role in the NMJ formation and function, either directly or indirectly through the AGRIN-LRP4-MuSk signaling.

## Supplementary Material

Supplementary figures and tables.

## Figures and Tables

**Table 1:** Main clinical features of the investigated patient in current study compared to patients with LPIN1 mutations in the literature

Features	Present patient	Reported patients	Percentage
Asian origin	+	15/48	31.3%
Consanguinity	-	17/48	35.4%
High Creatine kinase value	+	48/48	100%
Rhabdomyolysis	-	48/48	100%
Myohemoglobinuria	-	40/48	83.3%
Infantile onset (5y)	-	39/48	81.3%
Dyslipidemia	+	2/48	4.2%
Increasd Alanine aminotransferase (ALT) and Aspartate aminotransferase (AST)	+	12/48	25.0%
Increased lipid droplets in muscle fibers	+	23/48	47.9%
Myasthenia	+	20/48	41.7%
Myalgia	-	16/48	33.3%
Amyotrophy	+	2/48	4.2%
Predominance of type I muscle fibers	+	20/48	41.7%
Nerve demyelination	+	n.m.	n.m.
Reduced myelinated nerves to the gastrocnemius muscle	+	n.m.	n.m.
Abnormal Electromyography	+	n.m.	n.m.
Neurogenic injury of skeletal muscle	+	1/48	2.1%
Abnormal pyruvate/lactic acid ratio	+	n.m.	n.m.
Abnormal mitochondrial morphology of muscle fibers	+	n.m.	n.m.
Lysosomal polymerization of muscle fibers	+	n.m.	n.m.
History of kidney ailments	+	5/48	10.4%
History of heart ailments	+	2/48	4.2%

+ affirmative; - negative; +/- variable; n.m., not mentioned.

**Table 2:** Mutation information

Genome-level consequence		Gene		Gene-level consequence	
NC_000002.12:g.11944690 A>C		*LPIN1*		NM_145693:c.A2047C:p.I683L	
NC_000002.12:g.11955273 G>A				NM_145693:c.G2201A:p.R734Q	
***In silico* nonsynonymous mutations deleteriousness prediction algorithms and corresponding predictions**					
Mutation Variety	Polyphen2 HDIV	Polyphen2 HVAR	SIFT	MutationTaster	MutationAssessor
A>C	Damaging	Damaging	Damaging	Disease causing	Medium impact
G>A	Damaging	Damaging	Damaging	Disease causing	Medium impact
**Frequency of variants in large-scale population studies (mutant allele count/total alleles)**					
Mutation Variety	Exome Aggregation Consortium	TopMed	gnomAD-Exomes	gnomAD-Genomes	Chinese Millionome DataBase
A>C	0.001647% (2/121402)	0.00001 (1/125568)	0 (0/246214)	0 (0/30946)	0 (0/141431)
G>A	0 (0/121220)	0.00001 (1/125568)	0 (1/246214)	0 (1/30946)	0 (0/141431)

## Abbreviations

Lipin 1: phosphatidic acid phosphatase 1, PAP1
LPIN1: human LPIN1 gene
Lpin1: mouse LPIN1 gene
lpin1: zebrafish LPIN1 gene
PAP: phosphatidic acid phosphohydrolase
AChR: acetylcholine receptor
PA: phosphatidic acid
DAG: diacylglycerol
SCs): Schwann cells
TAG: triglycerides
PMNs: primary motor neurons
SMNs: secondary motor neurons
NMJ: neuromuscular junction
qRT-PCR: quantitative real time polymerase chain reaction
MOs: morpholinos
ISH: *in situ* hybridization
IHC: immunohistochemistry
DIG: Digoxigenin
CPK: creatine phosphokinase
ALT: Alanine aminotransferase
AST: Aspartate aminotransferase
SNPs: small nucleotide variants
C-LIP: C-terminal
α-BTX: α-bungrotoxin
MBP: Myelin basic protein
MuSK: muscle specific tyrosine kinase
dpf: day-post-fertilization
hpf: hour-post-fertilization
