# Supplementary material for: Lipin 1 deficiency causes adult-onset myasthenia with motor neuron dysfunction in humans and neuromuscular junction defects in zebrafish
Source: Theranostics. 2021 Jan 1;11(6):2788–805. doi: 10.7150/thno.53330 (PMC7806489; doi:10.7150/thno.53330)
Supplement: Supplementary file 1 — Supplementary figures and tables. [file thnov11p2788s1.pdf]

Supplementary materials

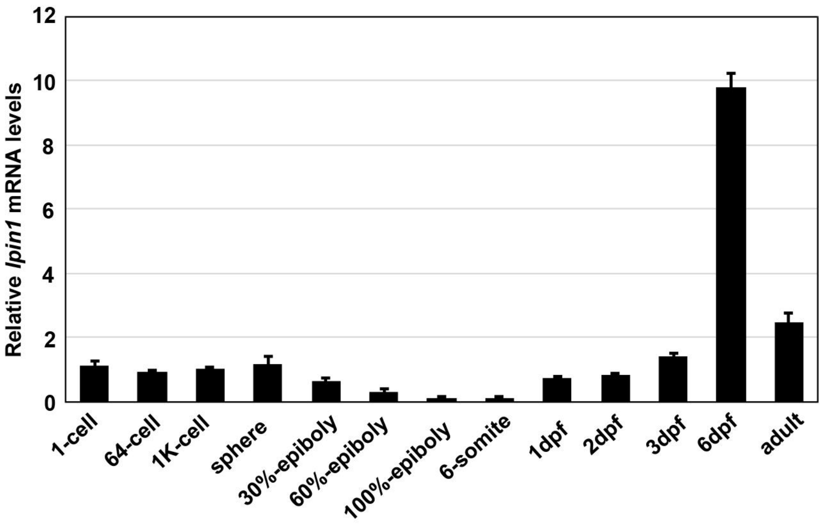

Figure S1. qRT-PCR results of *lpin1* transcript levels from 1-cell to adult stages.

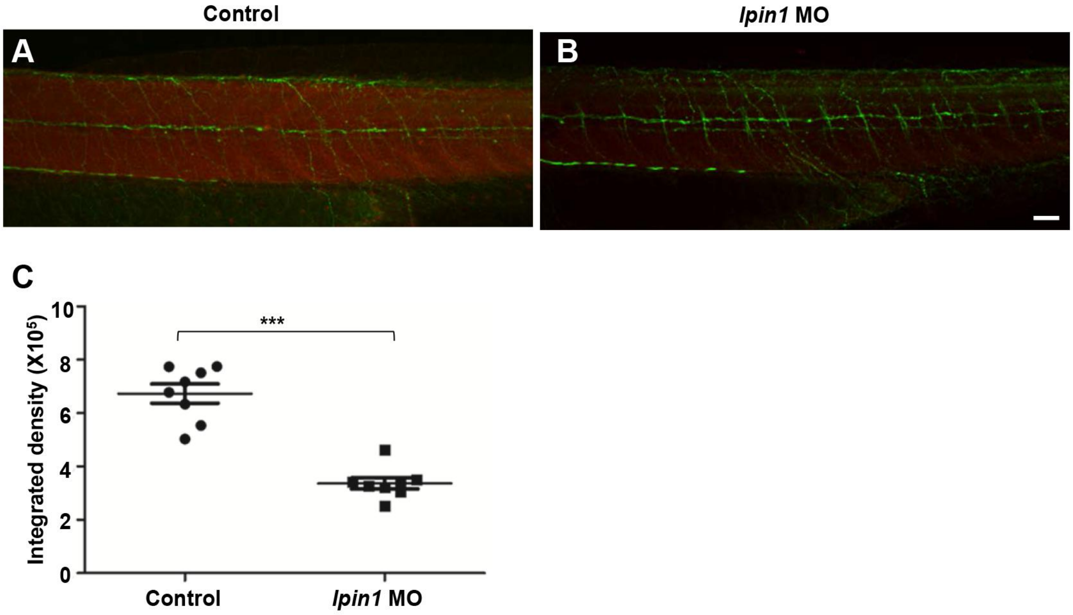

Figure S2. The expression of Lpin1 protein in somites. The expression of Lpin1 protein at somites in control embryos (A) was compared to *lpin1* morphants (B) at 24 hpf. (C) Quantification analysis by calculating the integrated density at the selected trunk areas (between somite 5 and 15) of the larvae further revealed a significantly decreased expression of Lpin1 protein in *lpin1* morphants (n=8 for each group). *** indicates $p < 0.001$ by Student's t-test. Scale bar: 100 μm.

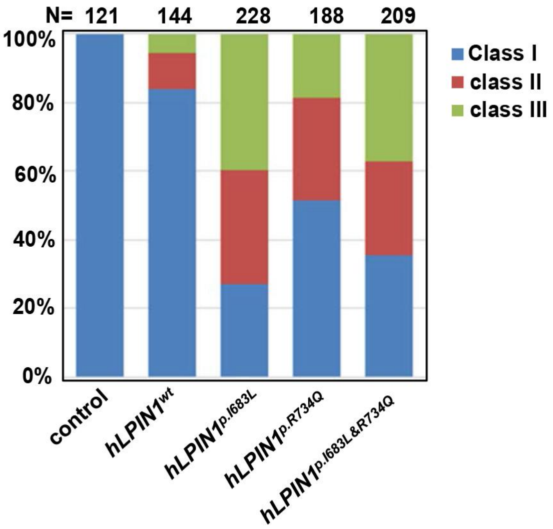

Figure S3. *LPIN1* mutant mRNAs affect skeletal muscle formation at 4 dpf. Overexpression of *hLPIN1*^{I683L}, *hLPIN*^{R734Q}, or *hLPIN1*$^{I683L\&\ R734Q}$, not *hLPIN1*^{wt} mRNA caused overall reduction in birefringence of the skeletal muscle.

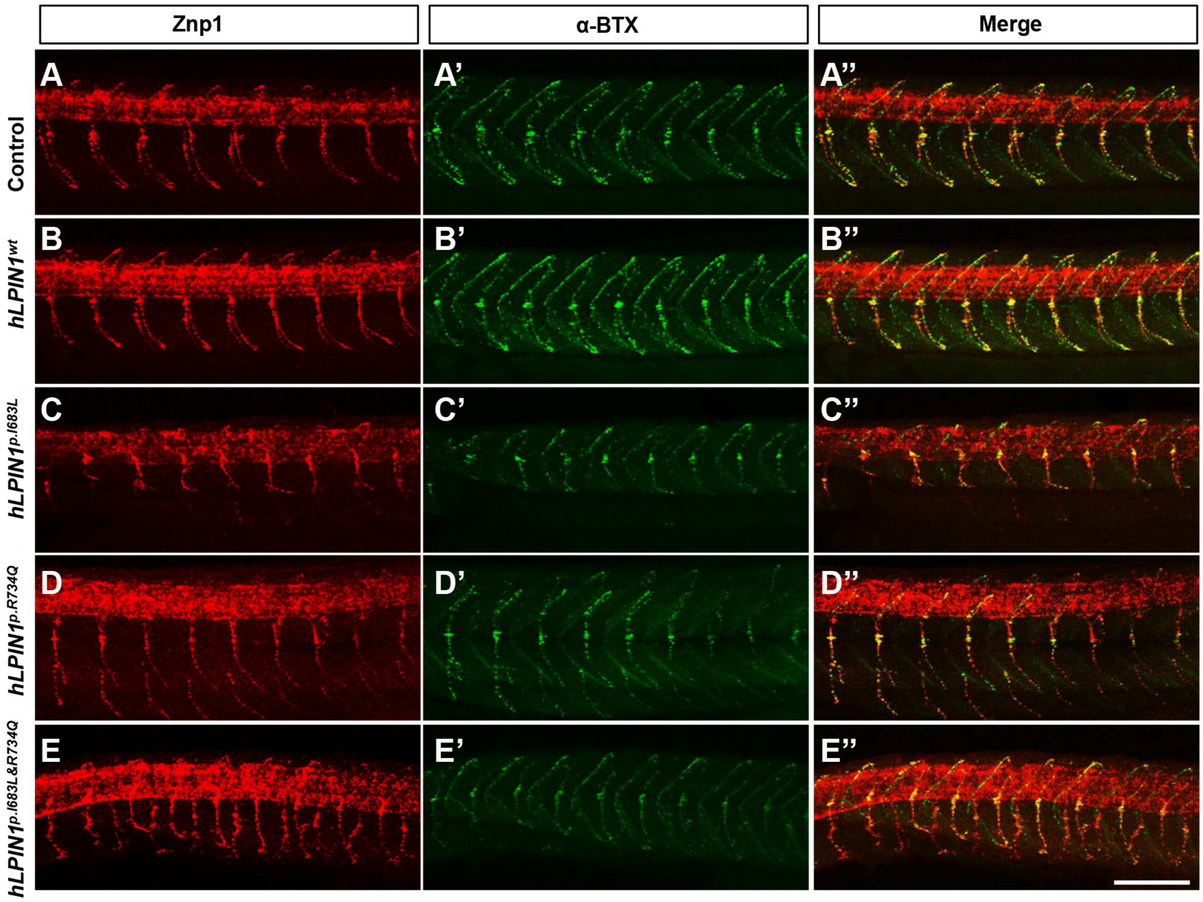

Figure S4. *LPIN1* mutant mRNAs affect PMNs development and AChR clustering in zebrafish embryos. Znp1 antibody staining (A, B, C, D, E) and α-BTX labeling (A', B', C', D', E') at 26 hpf. Control (A-A'') and *lpin1*^{wt} injected embryos (B-B'') exhibited normal PMNs outgrowth and post-synapse patterning. In embryos injected with *hLPIN1*^{I683L} (C-C''), *hLPIN*^{R734Q} (D-D''), or *hLPIN1*$^{I683L\&R734Q}$ (E-E''), PMNs outgrowth and post-synapse patterning were abnormal. Scale bar: 100 μm.

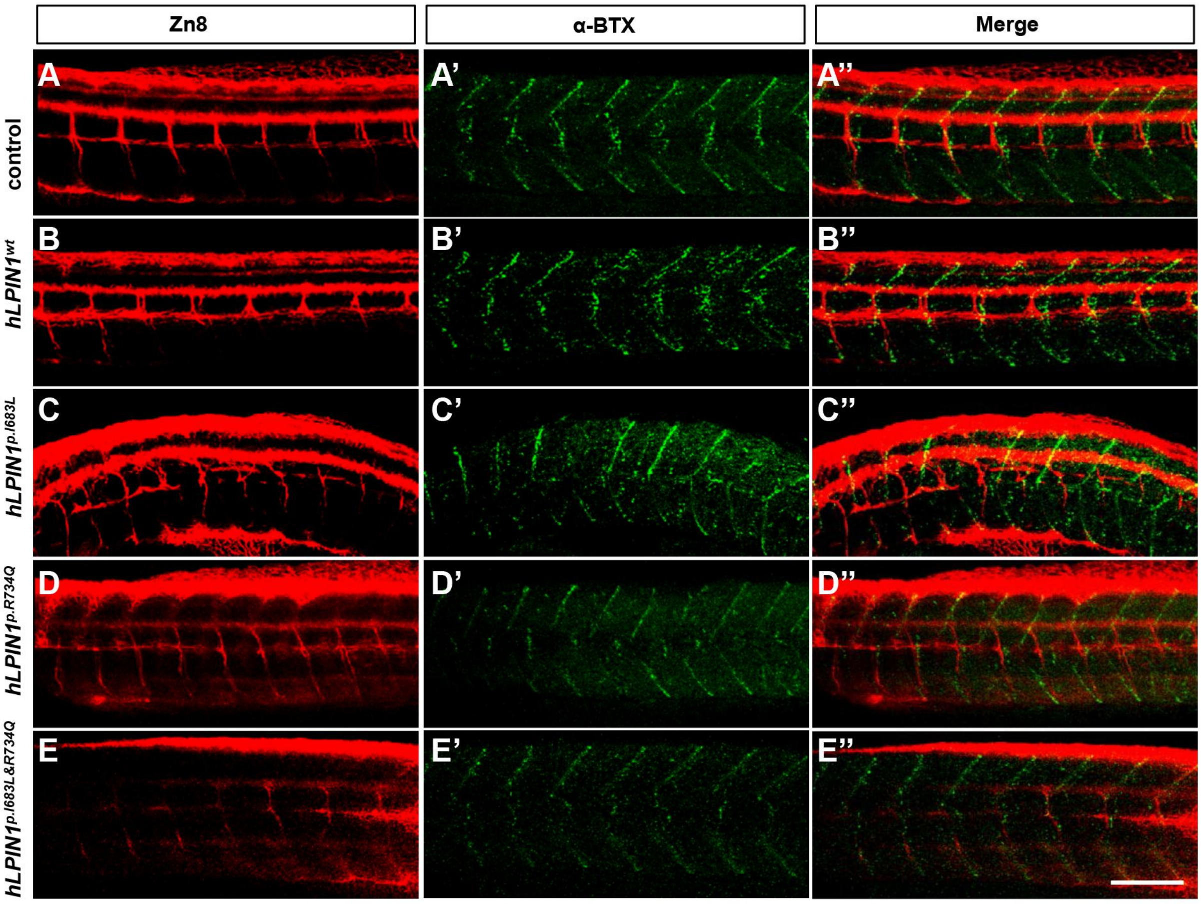

Figure S5. *LPIN1* mutant mRNAs affect SMNs development and AChR clustering in zebrafish embryos. Zn8 antibody staining (A, B, C, D, E) and α-BTX labeling (A', B', C', D', E') at 60 hpf. Control (A-A'') and *lpin1*^{wt} injected embryos (B-B'') exhibited normal SMNs outgrowth and post-synapse patterning. In embryos injected with *hLPIN1*^{I683L} (C-C''), *hLPIN*^{R734Q} (D-D''), or *hLPIN1*$^{I683L\&\ R734Q}$ (E-E''), SMNs outgrowth and post-synapse patterning were abnormal. Scale bar: 100 μm.

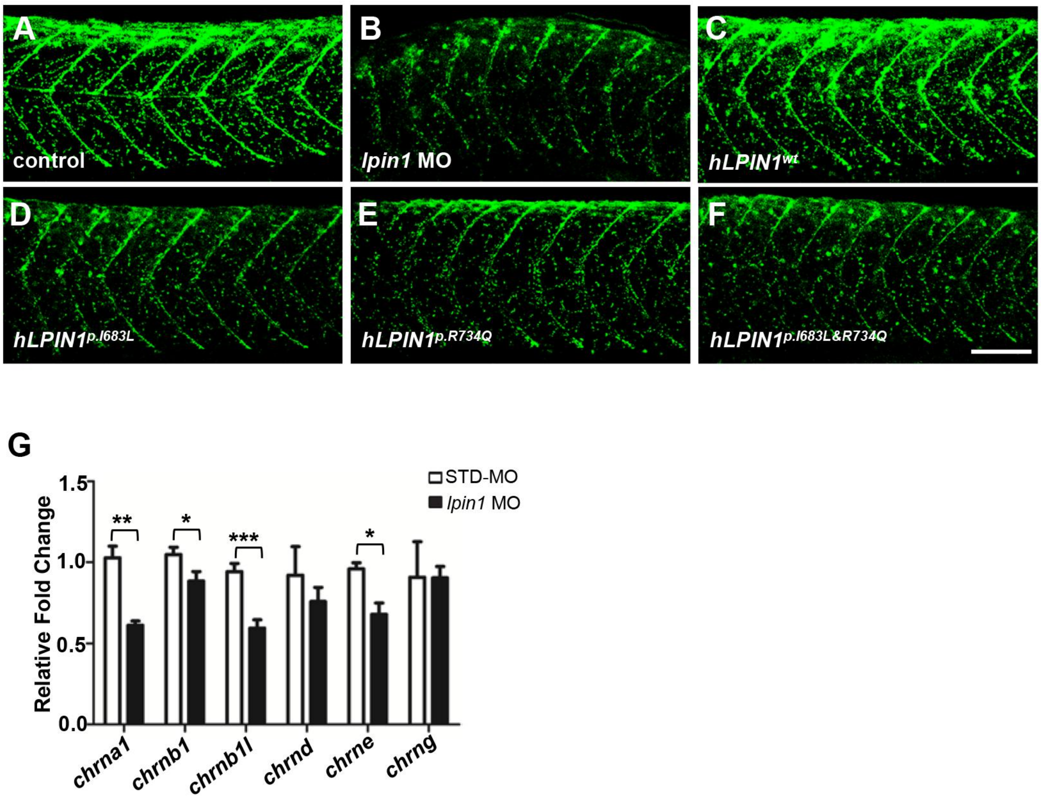

Figure S6. (A-F) AChRs clusters stained by α-BTX in *lpin1* MOs and *hLPIN1* mRNAs injected embryos at 5 dpf, lateral view, scale bar: 100 μm. (G) The expressions of nicotinic AChRs subunits markers, as determined by qRT-PCR, were significantly changed in *lpin1* morphants at 5 dpf.

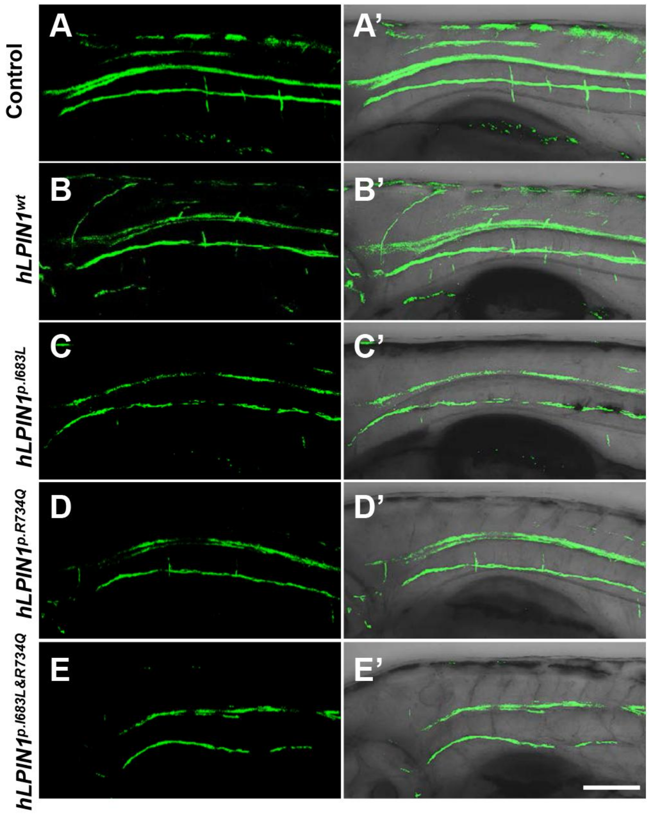

Figure S7. GFP signals of 5 dpf *Tg(mbp:eGFP)* larvae was visualized by fluorescent microscope when injected with different *hLPIN1* mRNAs, lateral view, scale bar: 100 μm.

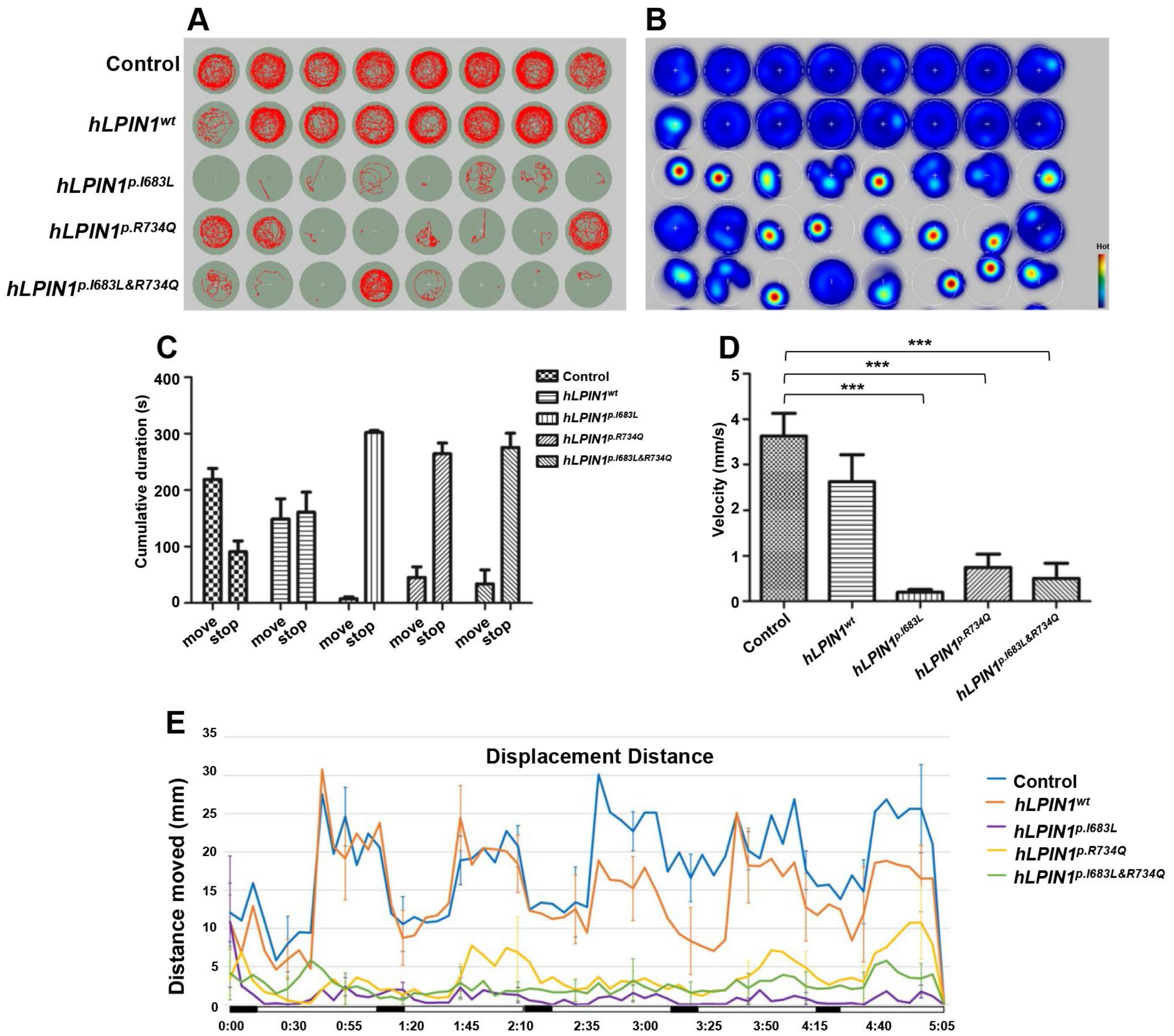

Figure S8. Locomotor deficits in the zebrafish larvae injected with mutant *LPIN1* mRNA. (A) The significant reduction in movement was observed in the zebrafish larvae injected with *hLPIN1*^{I683L}, *hLPIN*^{R734Q} and *hLPIN1*$^{I683L\&R734Q}$ respectively, compared to the control embryos and the *hLPIN1*^{wt} -injected larvae. Total movement was shown for a 5 min recording epoch. (B) Heat map plots where the activity levels of movement were represented by the intensity of blue color. Red dots indicated the long rest time without any movement. (C) Cumulative duration records showed that the larvae injected with the mRNA of *hLPIN1*^{I683L}, *hLPIN*^{R734Q} and *hLPIN1*$^{I683L\&\ R734Q}$ respectively exhibited poor activity. (D) Velocity was dramatically decreased in the zebrafish larvae injected with *hLPIN1*^{I683L}, *hLPIN*^{R734Q} or *hLPIN1*$^{I683L\&\ R734Q}$

but not *hLPIN1*^{wt}; as compared to uninjected controls. (E) Total distance travelled during a 5 min startle response analysis as follows：20 s spontaneous movement tracking followed by cycle stimulation with light on 5 s, interval 1 s, trigger tap 1 s, and interval 48 s; repeated 5 cycles. Data shown are means of distance moved in 5 s ± SE (n=8 for each group of larva at 5 dpf). Assays were carried out in flat-bottom 48-well plates.

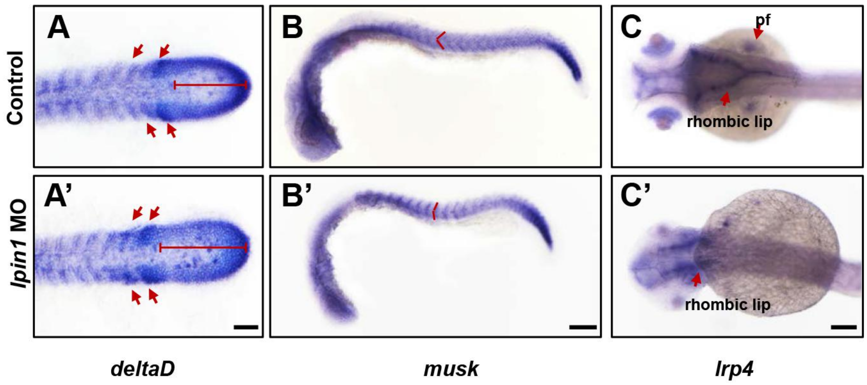

Figure S9. The expression of *deltaD*, *musk*, *lrp4* were affected in *lpin1* morphants. The expression of somite marker *deltaD* in control embryos (A) and *lpin1* morphant (A') embryos at 8-somite stage. Red arrows indicate newly formed somites and red bars indicate the length of presomitic mesoderm (psm). Control embryos (B) and *lpin1* morphant (B') embryos at 25 hpf were stained for *musk*. The expression of *lrp4* (C, C') in rhombic lip and pectoral fin was reduced in *lpin1* morphants at 50 hpf.

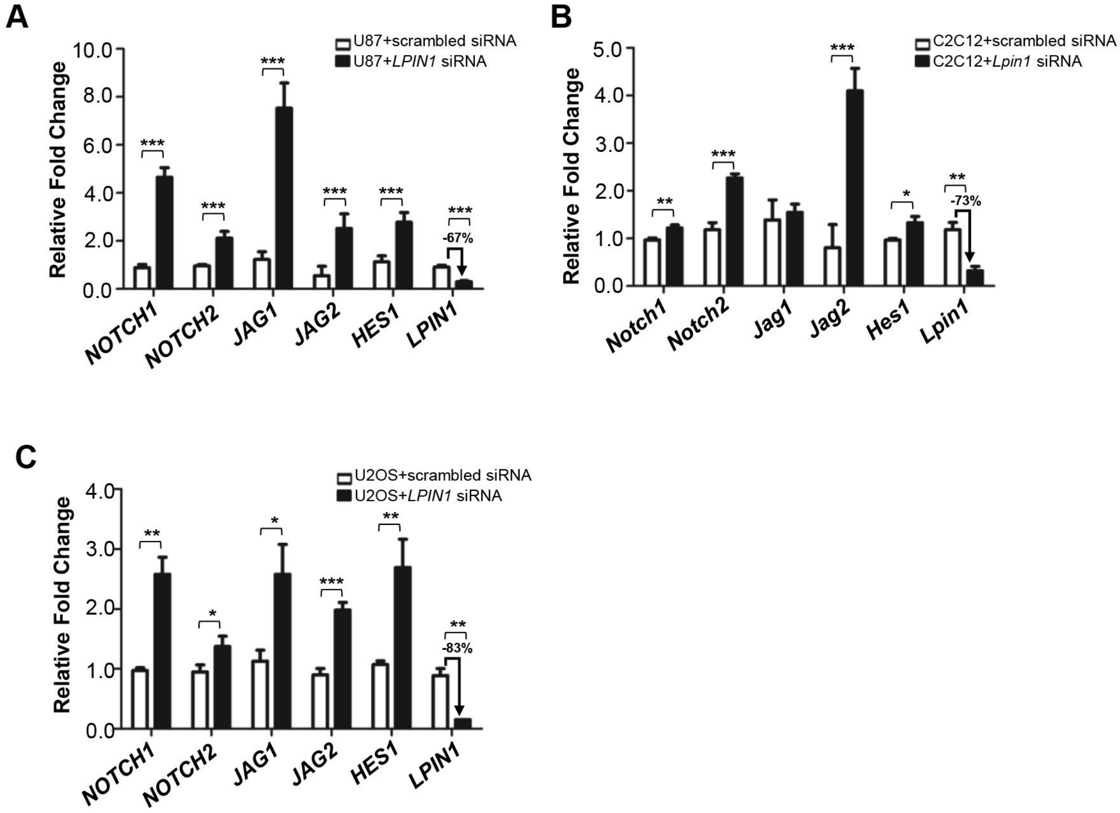

Figure S10. *LPIN1* knockdown in different cell lines triggered abnormal NOTCH activation. (A) *LPIN1* siRNA in human primary glioblastoma U87 cells achieved 67% knockdown efficiency with significantly upregulated expressions of NOTCH signaling components. (B) *LPIN1* siRNA in mouse myoblast C2C12 cells achieved 73% knockdown efficiency with significantly upregulated expressions of NOTCH signaling components. (C) *LPIN1* siRNA in human osteosarcoma U2OS cells achieved 83% knockdown efficiency with significantly upregulated expressions of NOTCH signaling components. Values in (A-C) represent means ± SE of data from three independent experiments, $*p < 0.05$, $**p < 0.01$, and $***p < 0.001$ (Student's t-test).

Supplementary Table S1: List of all primers, morpholino sequences, siRNAs sequences

Genomic DNA primer sequences

Gene	Exon	Forward	Reverse
LPIN1	E17	TGTCACCACGCAGTACCAAG	CACCAGAATTCGAACCGTGC
	E19	GAGTACAAGGGGCTCAAGCG	GGAAGAGAGCGGGTCTGAAC

Morpholino sequences

Name	sequence
lpin1- MO	ATATTCTGTTTGTTCTGACCTGCTG

siRNAs sequences

Gene name	Forward	Reverse
LPIN1-Human-siRNA	GGAGAUAAUGGAGAAGCAUTT	AUGCUUCUCCAUUAUCUCCTT
Lpin1-Mouse-siRNA	GCAUCGACAUCAUUGUCAUTT	AUGACAAUGAUGUCGAUGCTT

Human cDNA primer sequences used for Quantitative PCR

Gene Name	Forward	Reverse
LPIN1	TGGGTGTTTGCAATACAAAGG	GCTCCTTCACGGTGACAAA
LRP4	GTTCCAATGACGCTGTTCCT	CCAATGGCGTAGCTGATATG
AXIN2	GCTGACGGATGATTCCATGT	ACTGCCCACACGATAAGGAG
PAX2A	CACTTGCGAGCTGACACCTT	TGCAGATAGACTCGACTTGACTT
MPZ	CATCGTGGTTTACACCGACAG	TGGAAGATCGAAATGGCATCTCT
ACHE	CCTGTCCTCGTCTGGATCTATG	AAGAAGCGGCCATCGTACAC
ISL1	AAGGACAAGAAGCGAAGCAT	TTCCTGTCATCCCCTGGATA
CHAT	GGAGCCTGAGCACGTCAT	CGGAAATTAATGACAACATCCA
NEUROD1	ATGACCAAATCGTACAGCGAG	GTTCATGGCTTCGAGGTCGT
NOTCH1	GGCAATCCGAGGACTATGAG	CTCAGAACGCACTCGTTGAT
NOTCH2	TGGTGGCAGAACTGATCAAC	CTGCCCAGTGAAGAGCAGAT
JAG1	GAATGGCAACAAAACTTGCAT	AGCCTTGTCGGCAAATAGC
JAG2	TCATCCCCTTCCAGTTCG	ATGCGACACTCGCTCGAT
HES1	GAAGCACCTCCGGAACCT	GTCACCTCGTTCATGCACTC
ACTIN	ATCAAGATTGCTCCTCCTGAG	CTGCTTGCTGATCCACATCTG

Mouse cDNA primer sequences used for Quantitative PCR

Gene Name	Forward	Reverse
Lpin1	GACGGTGAAGGAGCTCTACA	ATGCTTCTCCGTTGTCTCCC
Moyg	GAGACATCCCCCTATTTCTACCA	GCTCAGTCCGCTCATAGCC
Actα1	ACCGGGAGAAGATGACTCAA	CCACGCTCAGTGAGGATTTT
Actα2	CGCCATGTATGTGGCTATTCAG	CTTGATGTCACGGACAATCTC
Dll1	CCCATCCGATTCCCCTTCG	GGTTTTCTGTTGCGAGGTCATC
Mylpf	CCCCAGAAGAACTGCTCCAA	TGCAAAGGTGTCCCGAAGAT
Musk	TTCAGCGGGACTGAGAAACT	TGTCTTCCACGCTCAGAATG
Myh1	CGGAGTCAGGTGAATACTCACG	GAGCATGAGCTAAGGCACTCT
Dysf	CAGCCTGATTTTTCGAGGGGT	ACCACCACGAGAAGTTCTGAG
Ttn	GACACCACAAGGTGCAAAGTC	CCCACTGTTCTTGACCGTATCT
Notch1	GGATGCTGACTGCATGGAT	AATCATGAGGGGTGTGAAGC
Notch2	CCATTTCAAGTGTTCGTGTCC	CACATTCATCGATGTTCTCTTCA
Jag1	TGGCCGAGGTCCTACACTT	GCCTTTTCAATTATGCTATCAGG
Jag2	CTTTCACCCTCATCGTGGA	TCAGCAGCTCCTCATCTGG
Hes1	TGCCAGCTGATATAATGGAGAA	CCATGATAGGCTTTGATGACTTT
Actin	AAGGCCAACCGTGAAAAGAT	GTGGTACGACCAGAGGCATAC

Zebrafish cDNA primer sequences used for Quantitative PCR

Gene Name	Forward	Reverse
lpin1	AGACAAGAGAAGCCCACACT	CACTACTGCCCACTGACTGA
pou3f1	CTCCAACACCGACTGGACCAG	GTCTGCGAGTAAACCAGAGAC
egr1	CAACGCCACAGCACCTGAAGG	GATGCCCACCAGCCCACTCAC
egr2a	CAGGTCCACCTTCAGACTGC	CTTTGGCAGCTGCTGTTCTG
egr2b	GATAGCATCTATTCGGTGGAC	GATCACGCCCTCTGGGTTCCAG
apoea	GAGGGACAACATCAAGGCCAC	GTATTCAGTGACACGATCCTTC
ccnd1	CAGAGAAGTTGTGCATATACAC	CAGTATCTGCTTGGAGCTCTG
mpz	CTGGCAGTGGACTTCTCCAG	CAGGTGAAGGTTCCATTGTC
ppm22a	CACATGGAGATACGAGTTATG	GATGATGGCACCGCTCATCAC
myog	AGCACATTGATGAACCCCAAC	CGGGGACACAGTGATCAGAC
acta1a	GTGATGGTGTGACCCACAATG	GACCTGACCGTCAGGAAGCTC
acta2	CAGCCATGTATGTAGCTATTC	GACGAATGAGTAGCCTCGTTC
deltaC	AGAAATACTGAGCGCGGACT	CGAGTCTTCTTTTGGTGGCC
mylpfa	ATACCGTCTCGACATGGCAC	CCTCCAGCTCCTCATTCTTCA
musk	CAATAAGGTATTACGATTCCAG	CATGGCAGCACTGCTTTGGAC
myhz1.1	CCTCCAGCTCCTCATTCTTCA	ATGCTTCCAGTCCGAGATCC
ryr1a	CCTGTGAGGGTTTTGGGAAT	TCTGAGTAGGATAGCGTGGC

ttn	GAGTACCTCTTCCGTGTCACTG	CAAGTCAGGAACCATAGTGC
lrp4	ATCCCGGCGTGAGTAACC	TGTGCGGTCTCGATCTTCA
axin2	GTAGTGGGTCTGTCTGCCAA	ACTTCACTGTCATTGGCACTC
neurog1	GGGTTAAGAGCAAGACAGACG	GCGAAAAGGAGTAGTCACAGC
pax2a	GTGTGGAGAGTTTACGGAAGC	AGACTGGGCTTGACTTCGTC
ache	CTTCAATACACTGATTGGATG	CCTGAGTGGATGGGCGTCATC
isl1	GTGATGAGAGCACGTTCGAAGG	CTTGTGCACATGAGGTCGAAG
neurod1	GACTCGAGGATGAGGACGACG	CTCGATCTTGGAGAGCTTCTG
nrg1	CTGAAGGCAGGAGGGCTGATC	CGTCCTCCACTGTCACGCTC
jag1b	GTGTGGATGGTGAAAACTGGT	GGGAGAAGACTGACACTCATTTATATT
jag2b	GAGAAAAGAGCGCGAGAGAC	GGTTCCCACTGGTTGTTGAC
notch1a	ATGCAGAGCCGGATTCAG	GAATCCAGCCACGTCGTC
notch2	ATCGACGAGTGTGTCATTGG	GATGTCCTGCTCACAACGTG
her1	GCGACTGCGAGAGATCAAG	ATGGCATCTGGGGTCTCC
lim1	AGTCCGAGAAGAATGCGAAC	GGCCGTAGTACTCGCTTTGA
chrna1	GATGGCAACCTTGTGATCATC	GTGAGGAAGGAGAACAGCATG
chrnb1	GTGGCTTCCTGATATTGTTCTC	CATGTTGAAGATCCACTTCTG
chrnb1l	GACTGGCAGAATTGCTCAATG	CAGGTAGAAGGTGATGTCCTC
chrnd	GTAGATATTTACCTGGCTCTGAC	CCAGTACATATCTCCACTGG
chrne	CATACTACACAGTGTGGCTAC	CTGAGACCTGAAGACCAGTG
chrng	CAGAATTGCAGCATGGTGTTC	GATCTCCTGGTGCTCCAGCTC

Supplementary Table S2: Variants from exome analysis

	total	1000G	Function	synonymous	deleterious
SNP	112651	8214	2526	1601	817
Indel	15152	5834	414	414	414

Supplementary Table S3: List of all SNP variants

CHROM	POS	REF	ALT	GeneName	Gene	AAChange
1	2704254	T	C	TTC34	NM_001242672	TTC34:NM_001242672:exon2:c.A107G:p.H36R
1	3428582	G	A	MEGF6	NM_001409	MEGF6:NM_001409:exon8:c.C964T:p.R322W
1	6215695	G	A	CHD5	NM_015557	CHD5:NM_015557:exon4:c.C470T:p.T157M
1	6272311	G	C	RNF207	NM_207396	RNF207:NM_207396:exon14:c.G1317C:p.Q439H
1	7838187	A	G	VAMP3	NM_004781	VAMP3:NM_004781:exon4:c.A241G:p.I81V
1	9801308	C	T	CLSTN1	NM_001009566,NM_001302883,NM_014944	CLSTN1:NM_014944:exon9:c.G1333A:p.D445N,CLSTN1:NM_001009566:exon10:c.G1363A:p.D455N,CLSTN1:NM_001302883:exon10:c.G1363A:p.D455N
1	10521569	C	T	DFFA	NM_004401	DFFA:NM_004401:exon6:c.G974A:p.R325Q
1	11721244	G	A	FBXO44	NM_001014765,NM_001304790,NM_001304791,NM_033182,NM_183412,NM_183413	FBXO44:NM_183412:exon5:c.G557A:p.R186Q,FBXO44:NM_183413:exon5:c.G557A:p.R186Q,FBXO44:NM_001304790:exon6:c.G557A:p.R186Q,FBXO44:NM_001304791:exon6:c.G682A:p.G228S,FBXO44:NM_033182:exon6:c.G682A:p.G228S,FBXO44:NM_001014765:exon7:c.G682A:p.G228S
1	11856343	C	T	MTHFR	NM_005957	MTHFR:NM_005957:exon5:c.G700A:p.D234N
1	12907449	C	G	HNRNPCL3,HNRNPCL4,HNRNPCL1	NM_001013631,NM_001146181,NM_001302551	HNRNPCL3:NM_001146181:exon1:c.G694C:p.D232H,HNRNPCL4:NM_001302551:exon1:c.G694C:p.D232H,HNRNPCL1:NM_001013631:exon2:c.G694C:p.D232H
1	16461581	G	A	EPHA2	NM_004431	EPHA2:NM_004431:exon7:c.C1532T:p.T511M
1	17945875	G	A	ARHGEF10L	NM_001011722,NM_018125	ARHGEF10L:NM_001011722:exon8:c.G760A:p.V254I,ARHGEF10L:NM_018125:exon10:c.G877A:p.V293I
1	20640937	G	A	VWA5B1	NM_001039500	VWA5B1:NM_001039500:exon4:c.G415A:p.E139K
1	22222455	G	T	HSPG2	NM_001291860,NM_005529	HSPG2:NM_001291860:exon3:c.C204A:p.D68E,HSPG2:NM_005529:exon3:c.C204A:p.D68E

1	25729169	T	C	RHCE	NM_020485,NM_138616,NM_138617,NM_138618	RHCE:NM_020485:exon3:c.A404G:p.N135S,RHCE:NM_138616:exon3:c.A404G:p.N135S,RHCE:NM_138617:exon3:c.A404G:p.N135S,RHCE:NM_138618:exon3:c.A404G:p.N135S
1	26314810	G	A	PAFAH2	NM_000437	PAFAH2:NM_000437:exon4:c.C253T:p.R85C
1	29391485	C	T	EPB41	NM_001166005,NM_001166006,NM_001166007,NM_004437,NM_203342,NM_203343	.
1	32204476	G	A	BAI2	NM_001294335,NM_001294336	ADGRB2:NM_001294335:exon16:c.C2558T:p.P853L,ADGRB2:NM_001294336:exon16:c.C2558T:p.P853L
1	36772294	C	T	SH3D21	NM_001162530	SH3D21:NM_001162530:exon2:c.C100T:p.R34W
1	41107525	C	T	RIMS3	NM_014747	RIMS3:NM_014747:exon3:c.G73A:p.G25S
1	42902196	C	T	ZMYND12	NM_001146192,NM_032257	ZMYND12:NM_001146192:exon4:c.G283A:p.A95T,ZMYND12:NM_032257:exon5:c.G613A:p.A205T
1	47607807	A	G	CYP4A22	NM_001010969	CYP4A22:NM_001010969:exon4:c.A410G:p.Q137R
1	62189400	T	C	TM2D1	NM_032027	TM2D1:NM_032027:exon2:c.A223G:p.N75D
1	68564437	C	T	GNG12-AS1,WLS	NR_040077;NM_001002292	.
1	74716413	C	A	FPGT-TNNI3K,TNNI3K	NM_001112808,NM_001199327,NM_015978	TNNI3K:NM_015978:exon4:c.C293A:p.T98K,FPGT-TNNI3K:NM_001112808:exon6:c.C635A:p.T212K,FPGT-TNNI3K:NM_001199327:exon6:c.C635A:p.T212K
1	89414937	C	A	CCBL2	NM_001008661,NM_001008662	CCBL2:NM_001008662:exon10:c.G876T:p.W292C,CCBL2:NM_001008661:exon11:c.G978T:p.W326C
1	95699802	C	T	RWDD3	NM_001128142,NM_001199682,NM_015485	RWDD3:NM_001128142:exon1:c.C16T:p.Q6X,RWDD3:NM_001199682:exon1:c.C16T:p.Q6X,RWDD3:NM_015485:exon1:c.C16T:p.Q6X
1	117142613	C	T	IGSF3	NM_001007237,NM_001542	IGSF3:NM_001007237:exon7:c.G1979A:p.R660Q,IGSF3:NM_001542:exon7:c.G2039A:p.R680Q
1	117142641	G	A	IGSF3	NM_001007237,NM_001542	IGSF3:NM_001007237:exon7:c.C1951T:p.R651W,IGSF3:NM_001542:exon7:c.C2011T:p.R671W
1	117142700	C	A	IGSF3	NM_001007237,NM_001542	IGSF3:NM_001007237:exon7:c.G1892T:p.S631I,IGSF3:NM_001542:exon7:c.G1952T:p.S651I

1	117142736	A	G	IGSF3	NM_001007237,NM_001542	IGSF3:NM_001007237:exon7:c.T1856C:p.I619T,IGSF3:NM_001542:exon7:c.T1916C:p.I639T
1	117142868	C	T	IGSF3	NM_001007237,NM_001542	IGSF3:NM_001007237:exon7:c.G1724A:p.W575X,IGSF3:NM_001542:exon7:c.G1784A:p.W595X
1	117146504	G	A	IGSF3	NM_001007237,NM_001542	IGSF3:NM_001007237:exon6:c.C1366T:p.R456C,IGSF3:NM_001542:exon6:c.C1426T:p.R476C
1	117311140	A	G	CD2	NM_001767	CD2:NM_001767:exon5:c.A791G:p.K264R
1	120056870	G	A	HSD3B1	NM_000862	HSD3B1:NM_000862:exon4:c.G724A:p.D242N
1	143767513	A	T	PPIAL4G	NM_001123068	PPIAL4G:NM_001123068:exon1:c.T336A:p.F112L
1	144866643	G	A	PDE4DIP	NM_001198832,NM_001198834,NM_014644	PDE4DIP:NM_001198834:exon34:c.C5599T:p.R1867C,PDE4DIP:NM_014644:exon34:c.C5599T:p.R1867C,PDE4DIP:NM_001198832:exon36:c.C5281T:p.R1761C
1	144868170	C	T	PDE4DIP	NM_001198832,NM_001198834,NM_014644	PDE4DIP:NM_001198834:exon33:c.G5269A:p.A1757T,PDE4DIP:NM_014644:exon33:c.G5269A:p.A1757T,PDE4DIP:NM_001198832:exon35:c.G4951A:p.A1651T
1	144874038	T	C	PDE4DIP	NM_001198832,NM_001198834,NM_014644	.
1	144874815	T	C	PDE4DIP	NM_001198832,NM_001198834,NM_014644	PDE4DIP:NM_001198834:exon30:c.A4793G:p.H1598R,PDE4DIP:NM_014644:exon30:c.A4793G:p.H1598R,PDE4DIP:NM_001198832:exon33:c.A4661G:p.H1554R
1	144882823	C	T	PDE4DIP	NM_001198832,NM_001198834,NM_014644	PDE4DIP:NM_001198834:exon24:c.G3196A:p.A1066T,PDE4DIP:NM_014644:exon24:c.G3196A:p.A1066T,PDE4DIP:NM_001198832:exon27:c.G3394A:p.A1132T
1	144912233	C	T	PDE4DIP	NM_001002811,NM_001002812,NM_001198832,NM_001198834,NM_014644	PDE4DIP:NM_001002811:exon11:c.G2531A:p.R844H,PDE4DIP:NM_001002812:exon15:c.G2042A:p.R681H,PDE4DIP:NM_001198834:exon15:c.G2042A:p.R681H,PDE4DIP:NM_014644:exon15:c.G2042A:p.R681H,PDE4DIP:NM_001198832:exon18:c.G2240A:p.R747H
1	144915561	G	A	PDE4DIP	NM_001002811,NM_001002812,NM_001198832,NM_001198834,NM_014644	PDE4DIP:NM_001002811:exon10:c.C2353T:p.R785X,PDE4DIP:NM_001002812:exon14:c.C1864T:p.R622X,PDE4DIP:NM_001198834:exon14:c.C1864T:p.R622X,PDE4DIP:NM_014644:exon14:c.C1864T:p.R622X,PDE4DIP:NM_001198832:exon17:c.C2062T:p.R688X

1	144916676	C	T	PDE4DIP	NM_001002811,NM_001002812,NM_001198832,NM_001198834,NM_014644	PDE4DIP:NM_001002811:exon9:c.G2168A:p.W723X,PDE4DIP:NM_001002812:exon13:c.G1679A:p.W560X,PDE4DIP:NM_001198834:exon13:c.G1679A:p.W560X,PDE4DIP:NM_014644:exon13:c.G1679A:p.W560X,PDE4DIP:NM_001198832:exon16:c.G1877A:p.W626X
1	144916748	C	G	PDE4DIP	NM_001002811,NM_001002812,NM_001198832,NM_001198834,NM_014644	PDE4DIP:NM_001002811:exon9:c.G2096C:p.S699T,PDE4DIP:NM_001002812:exon13:c.G1607C:p.S536T,PDE4DIP:NM_001198834:exon13:c.G1607C:p.S536T,PDE4DIP:NM_014644:exon13:c.G1607C:p.S536T,PDE4DIP:NM_001198832:exon16:c.G1805C:p.S602T
1	144918957	T	A	PDE4DIP	NM_001002811,NM_001002812,NM_001198832,NM_001198834,NM_014644	PDE4DIP:NM_001002811:exon6:c.A1718T:p.E573V,PDE4DIP:NM_001002812:exon10:c.A1229T:p.E410V,PDE4DIP:NM_001198834:exon10:c.A1229T:p.E410V,PDE4DIP:NM_014644:exon10:c.A1229T:p.E410V,PDE4DIP:NM_001198832:exon13:c.A1427T:p.E476V
1	144922523	C	T	PDE4DIP	NM_001002811,NM_001002812,NM_001198832,NM_001198834,NM_014644	PDE4DIP:NM_001002811:exon3:c.G1373A:p.R458H,PDE4DIP:NM_001002812:exon7:c.G884A:p.R295H,PDE4DIP:NM_001198834:exon7:c.G884A:p.R295H,PDE4DIP:NM_014644:exon7:c.G884A:p.R295H,PDE4DIP:NM_001198832:exon10:c.G1082A:p.R361H
1	144922583	G	A	PDE4DIP	NM_001002811,NM_001002812,NM_001198832,NM_001198834,NM_014644	PDE4DIP:NM_001002811:exon3:c.C1313T:p.S438L,PDE4DIP:NM_001002812:exon7:c.C824T:p.S275L,PDE4DIP:NM_001198834:exon7:c.C824T:p.S275L,PDE4DIP:NM_014644:exon7:c.C824T:p.S275L,PDE4DIP:NM_001198832:exon10:c.C1022T:p.S341L
1	144930940	T	C	PDE4DIP	NM_001002811	PDE4DIP:NM_001002811:exon1:c.A769G:p.K257E
1	144931330	C	T	PDE4DIP	NM_001002811	PDE4DIP:NM_001002811:exon1:c.G379A:p.A127T
1	145021150	T	C	PDE4DIP	NM_001198832,NM_022359	PDE4DIP:NM_001198832:exon2:c.A38G:p.D13G,PDE4DIP:NM_022359:exon2:c.A251G:p.D84G
1	145075683	C	T	PDE4DIP	NM_022359	PDE4DIP:NM_022359:exon1:c.G180A:p.W60X
1	145103947	T	C	SEC22B	NM_004892	UNKNOWN
1	145109549	G	T	SEC22B	NM_004892	UNKNOWN
1	145109583	C	A	SEC22B	NM_004892	UNKNOWN
1	145109661	G	A	SEC22B	NM_004892	UNKNOWN
1	145112414	T	C	SEC22B	NM_004892	UNKNOWN
1	145112420	C	T	SEC22B	NM_004892	UNKNOWN

1	145112428	C	T	SEC22B	NM_004892	UNKNOWN
1	145112485	C	T	SEC22B	NM_004892	UNKNOWN
1	145115810	A	G	SEC22B	NM_004892	UNKNOWN
1	145115820	T	C	SEC22B	NM_004892	UNKNOWN
1	149859105	G	A	HIST2H2AB	NM_175065	HIST2H2AB:NM_175065:exon1:c.C362T:p.T121M
1	155217643	C	T	FAM189B	NM_001267608,NM_006589,NM_198264	FAM189B:NM_198264:exon9:c.G1649A:p.R550H,FAM189B:NM_001267608:exon11:c.G1883A:p.R628H,FAM189B:NM_006589:exon12:c.G1937A:p.R646H
1	161165952	C	T	ADAMTS4	NM_005099	.
1	179965908	T	A	CEP350	NM_014810	CEP350:NM_014810:exon6:c.T616A:p.L206M
1	196309515	T	C	KCNT2	NM_001287819,NM_001287820,NM_198503	KCNT2:NM_001287820:exon15:c.A1589G:p.H530R,KCNT2:NM_001287819:exon16:c.A1739G:p.H580R,KCNT2:NM_198503:exon16:c.A1739G:p.H580R
1	196918703	T	G	CFHR2	NM_005666	CFHR2:NM_005666:exon2:c.T177G:p.F59L
1	197069932	C	A	ASPM	NM_018136	ASPM:NM_018136:exon18:c.G8449T:p.A2817S
1	197564470	G	A	DENND1B	NM_001195215,NM_001300858,NM_144977	.
1	201178819	G	A	IGFN1	NM_001164586	IGFN1:NM_001164586:exon12:c.G4798A:p.A1600T
1	201181220	C	T	IGFN1	NM_001164586	IGFN1:NM_001164586:exon12:c.C7199T:p.T2400M
1	201778370	C	T	NAV1	NM_001167738,NM_020443	NAV1:NM_001167738:exon18:c.C3104T:p.P1035L,NAV1:NM_020443:exon21:c.C4286T:p.P1429L
1	201869461	C	T	LMOD1	NM_012134	LMOD1:NM_012134:exon2:c.G680A:p.R227H
1	205353468	G	A	LEMD1	NM_001199050,NM_001199051	LEMD1:NM_001199051:exon4:c.C172T:p.R58C,LEMD1:NM_001199050:exon5:c.C295T:p.R99C
1	206516261	C	T	SRGAP2B,SRGAP2,SRGAP2C	NM_001170637,NM_001271870,NM_001271872,NM_001300952,NM_015326	UNKNOWN

1	206566903	G	A	SRGAP2B, SRGAP2,SRGAP2C	NM_001170637,NM_001271870,NM_001271872,NM_001300952,NM_015326	UNKNOWN
1	226570838	T	G	PARP1	NM_001618	PARP1:NM_001618:exon8:c.A1058C:p.Q353P
1	229662992	A	G	ABCB10	NM_012089	ABCB10:NM_012089:exon9:c.T1709C:p.I570T
1	232581501	C	T	SIPA1L2	NM_020808	SIPA1L2:NM_020808:exon9:c.G3127A:p.V1043M
1	236702306	C	T	LGALS8	NM_006499,NM_201543,NM_201544,NM_201545	LGALS8:NM_201544:exon4:c.C262T:p.R88W,LGALS8:NM_006499:exon5:c.C262T:p.R88W,LGALS8:NM_201543:exon5:c.C262T:p.R88W,LGALS8:NM_201545:exon5:c.C262T:p.R88W
1	240370985	C	T	FMN2	NM_001305424,NM_020066	FMN2:NM_020066:exon5:c.C2873T:p.P958L,FMN2:NM_001305424:exon6:c.C2885T:p.P962L
1	241718903	T	C	KMO	NM_003679	.
1	243328887	G	T	CEP170	NM_001042404,NM_001042405,NM_014812	CEP170:NM_001042404:exon12:c.C2081A:p.S694X,CEP170:NM_001042405:exon12:c.C2081A:p.S694X,CEP170:NM_014812:exon13:c.C2375A:p.S792X
1	245222729	C	T	EFCAB2	NM_001143943,NM_001290327,NM_032328	EFCAB2:NM_001143943:exon3:c.C152T:p.T51M,EFCAB2:NM_001290327:exon4:c.C182T:p.T61M,EFCAB2:NM_032328:exon4:c.C152T:p.T51M
1	245861491	C	T	KIF26B	NM_018012	KIF26B:NM_018012:exon13:c.C5908T:p.R1970C
1	248262834	C	T	OR2L13	NM_001304535,NM_175911	OR2L13:NM_001304535:exon2:c.C157T:p.R53C,OR2L13:NM_175911:exon3:c.C157T:p.R53C
2	10186320	G	A	KLF11	NM_001177716,NM_001177718,NM_003597	KLF11:NM_001177716:exon2:c.G35A:p.R12Q,KLF11:NM_001177718:exon2:c.G35A:p.R12Q,KLF11:NM_003597:exon2:c.G86A:p.R29Q
2	11944690	A	C	LPIN1	NM_001261427,NM_001261428,NM_145693	LPIN1:NM_145693:exon15:c.A2047C:p.I683L,LPIN1:NM_001261427:exon16:c.A2065C:p.I689L,LPIN1:NM_001261428:exon17:c.A2302C:p.I768L
2	11955273	G	A	LPIN1	NM_001261427,NM_001261428,NM_145693	LPIN1:NM_145693:exon17:c.G2201A:p.R734Q,LPIN1:NM_001261427:exon18:c.G2219A:p.R740Q,LPIN1:NM_001261428:exon19:c.G2456A:p.R819Q
2	18741450	A	T	RDH14	NM_020905	RDH14:NM_020905:exon1:c.T389A:p.L130H
2	20205933	C	T	MATN3	NM_002381	MATN3:NM_002381:exon2:c.G362A:p.R121Q
2	37296064	C	A	HEATR5B	NM_019024	HEATR5B:NM_019024:exon8:c.G937T:p.V313F
2	38302213	G	C	CYP1B1	NM_000104	CYP1B1:NM_000104:exon2:c.C319G:p.L107V

2	39095403	C	T	DHX57	NM_198963	DHX57:NM_198963:exon2:c.G145A:p.G49S
2	44586653	G	A	PREPL	NM_001042385,NM_001042386,NM_001171603,NM_001171606,NM_006036	PREPL:NM_001042385:exon1:c.C202T:p.R68W,PREPL:NM_001042386:exon1:c.C202T:p.R68W,PREPL:NM_006036:exon1:c.C202T:p.R68W,PREPL:NM_001171603:exon2:c.C202T:p.R68W,PREPL:NM_001171606:exon2:c.C202T:p.R68W
2	53941512	T	C	ASB3,GPR75-ASB3	NM_001164165,NM_001201965,NM_016115,NM_145863	.
2	54561964	A	C	C2orf73	NM_001100396	C2orf73:NM_001100396:exon2:c.A37C:p.I13L
2	61348420	G	A	KIAA1841	NM_001129993	.
2	68389669	C	G	PNO1	NM_020143	.
2	71036500	C	T	CLEC4F	NM_173535	CLEC4F:NM_173535:exon7:c.G1673A:p.G558E
2	73762010	C	G	ALMS1	NM_015120	ALMS1:NM_015120:exon12:c.C9838G:p.Q3280E
2	74687644	C	T	WBP1	NM_012477	WBP1:NM_012477:exon4:c.C646T:p.L216F
2	74709546	C	T	CCDC142	NM_032779	CCDC142:NM_032779:exon1:c.G419A:p.W140X
2	74717206	C	T	TTC31	NM_022492	TTC31:NM_022492:exon3:c.C184T:p.R62W
2	75899101	T	C	GCFC2	NM_001201334,NM_003203	GCFC2:NM_001201334:exon14:c.A1424G:p.E475G,GCFC2:NM_003203:exon14:c.A1931G:p.E644G
2	86697366	T	A	KDM3A	NM_001146688,NM_018433	KDM3A:NM_001146688:exon11:c.T1559A:p.L520H,KDM3A:NM_018433:exon11:c.T1559A:p.L520H
2	97217534	G	C	ARID5A	NM_212481	ARID5A:NM_212481:exon7:c.G1269C:p.K423N
2	97877478	G	A	ANKRD36	NM_001164315	ANKRD36:NM_001164315:exon58:c.G3469A:p.V1157M
2	100199386	G	T	AFF3	NM_001025108,NM_002285	AFF3:NM_001025108:exon16:c.C2742A:p.H914Q,AFF3:NM_002285:exon16:c.C2667A:p.H889Q
2	102626142	C	G	IL1R2	NM_001261419,NM_004633	IL1R2:NM_001261419:exon3:c.C186G:p.S62R,IL1R2:NM_004633:exon3:c.C186G:p.S62R
2	103095699	G	A	SLC9A4	NM_001011552	SLC9A4:NM_001011552:exon2:c.G658A:p.V220M
2	112922638	T	A	FBLN7	NM_001128165,NM_153214	FBLN7:NM_001128165:exon3:c.T296A:p.V99E,FBLN7:NM_153214:exon3:c.T296A:p.V99E
2	120414023	A	G	CFAP221	NM_001271049	CFAP221:NM_001271049:exon24:c.A2500G:p.N834D
2	130951949	G	A	TUBA3E	NM_207312	TUBA3E:NM_207312:exon4:c.C466T:p.R156W

2	135744469	T	C	MAP3K19	NM_001018044,NM_025052	MAP3K19:NM_001018044:exon5:c.A1634G:p.N545S,MAP3K19:NM_025052:exon7:c.A1973G:p.N658S
2	136480126	A	G	R3HDM1	NM_001282798,NM_001282799,NM_001282800,NM_015361	.
2	148676144	A	C	ACVR2A	NM_001278579,NM_001278580,NM_001616	ACVR2A:NM_001278580:exon7:c.A621C:p.K207N,ACVR2A:NM_001616:exon7:c.A945C:p.K315N,ACVR2A:NM_001278579:exon8:c.A945C:p.K315N
2	152484154	C	G	NEB	NM_001164507,NM_001164508,NM_001271208,NM_004543	NEB:NM_004543:exon65:c.G9261C:p.K3087N,NEB:NM_001164507:exon69:c.G10026C:p.K3342N,NEB:NM_001164508:exon69:c.G10026C:p.K3342N,NEB:NM_001271208:exon69:c.G10026C:p.K3342N
2	153532910	T	C	PRPF40A	NM_017892	.
2	155102453	G	C	GALNT13	NM_001301627,NM_052917	GALNT13:NM_001301627:exon5:c.G815C:p.R272T,GALNT13:NM_052917:exon7:c.G815C:p.R272T
2	160181398	G	A	BAZ2B	NM_001289975,NM_013450	BAZ2B:NM_001289975:exon35:c.C6169T:p.L2057F,BAZ2B:NM_013450:exon36:c.C6277T:p.L2093F
2	163144647	T	C	IFIH1	NM_022168	IFIH1:NM_022168:exon5:c.A1093G:p.K365E
2	165381518	A	G	GRB14	NM_001303422,NM_004490	GRB14:NM_001303422:exon4:c.T413C:p.L138S,GRB14:NM_004490:exon5:c.T674C:p.L225S
2	165947256	C	T	SCN3A	NM_001081676,NM_001081677,NM_006922	SCN3A:NM_001081676:exon28:c.G5260A:p.D1754N,SCN3A:NM_001081677:exon28:c.G5260A:p.D1754N,SCN3A:NM_006922:exon28:c.G5407A:p.D1803N
2	166912912	T	G	SCN1A	NM_001165963,NM_001165964,NM_001202435,NM_006920	.
2	169801131	G	A	ABCB11	NM_003742	ABCB11:NM_003742:exon21:c.C2594T:p.A865V
2	170762672	A	G	UBR3	NM_172070	UBR3:NM_172070:exon10:c.A1777G:p.R593G
2	178095985	C	T	NFE2L2	NM_001145412,NM_001145413,NM_006164	NFE2L2:NM_001145412:exon5:c.G1298A:p.R433H,NFE2L2:NM_001145413:exon5:c.G1277A:p.R426H,NFE2L2:NM_006164:exon5:c.G1346A:p.R449H
2	179414895	C	T	TTN	NM_001256850,NM_001267550,NM_003319,NM_133378,NM_133432,NM_133437	TTN:NM_003319:exon165:c.G64475A:p.R21492Q,TTN:NM_133432:exon166:c.G64850A:p.R21617Q,TTN:NM_133437:exon166:c.G65051A:p.R21684Q,TTN:NM_133378:exon286:c.G83966A:p.R27989Q,TTN:NM_001256850:exon287:c.G86747A:p.R28916Q,TTN:NM_001267550:exon337:c.G91670A:p.R30557Q

2	179422112	G	A	TTN	NM_001256850,NM_001267550,NM_003319,NM_133378,NM_133432,NM_133437	TTN:NM_003319:exon157:c.C60682T:p.R20228C,TTN:NM_133432:exon158:c.C61057T:p.R20353C,TTN:NM_133437:exon158:c.C61258T:p.R20420C,TTN:NM_133378:exon278:c.C80173T:p.R26725C,TTN:NM_001256850:exon279:c.C82954T:p.R27652C,TTN:NM_001267550:exon329:c.C87877T:p.R29293C
2	179430744	C	A	TTN	NM_001256850,NM_001267550,NM_003319,NM_133378,NM_133432,NM_133437	TTN:NM_003319:exon154:c.G52920T:p.E17640D,TTN:NM_133432:exon155:c.G53295T:p.E17765D,TTN:NM_133437:exon155:c.G53496T:p.E17832D,TTN:NM_133378:exon275:c.G72411T:p.E24137D,TTN:NM_001256850:exon276:c.G75192T:p.E25064D,TTN:NM_001267550:exon326:c.G80115T:p.E26705D
2	179441340	C	T	TTN	NM_001256850,NM_001267550,NM_003319,NM_133378,NM_133432,NM_133437	TTN:NM_003319:exon153:c.G42436A:p.E14146K,TTN:NM_133432:exon154:c.G42811A:p.E14271K,TTN:NM_133437:exon154:c.G43012A:p.E14338K,TTN:NM_133378:exon274:c.G61927A:p.E20643K,TTN:NM_001256850:exon275:c.G64708A:p.E21570K,TTN:NM_001267550:exon325:c.G69631A:p.E23211K
2	179466433	T	G	TTN	NM_001256850,NM_001267550,NM_003319,NM_133378,NM_133432,NM_133437	TTN:NM_003319:exon114:c.A28189C:p.K9397Q,TTN:NM_133432:exon115:c.A28564C:p.K9522Q,TTN:NM_133437:exon115:c.A28765C:p.K9589Q,TTN:NM_133378:exon235:c.A47680C:p.K15894Q,TTN:NM_001256850:exon236:c.A50461C:p.K16821Q,TTN:NM_001267550:exon286:c.A55384C:p.K18462Q
2	179631326	T	C	TTN	NM_001256850,NM_001267550,NM_003319,NM_133378,NM_133379,NM_133432,NM_133437	TTN:NM_003319:exon40:c.A9347G:p.Q3116R,TTN:NM_133432:exon40:c.A9347G:p.Q3116R,TTN:NM_133437:exon40:c.A9347G:p.Q3116R,TTN:NM_001256850:exon41:c.A9485G:p.Q3162R,TTN:NM_001267550:exon41:c.A9485G:p.Q3162R,TTN:NM_133378:exon41:c.A9485G:p.Q3162R,TTN:NM_133379:exon41:c.A9485G:p.Q3162R
2	180829352	T	C	CWC22	NM_020943	CWC22:NM_020943:exon13:c.A1319G:p.Q440R
2	198414992	C	T	HSPE1-MOB4,MOB4	NM_001100819,NM_001202485,NM_001204094,NM_015387,NM_199482	.
2	203620264	C	T	FAM117B	NM_173511	FAM117B:NM_173511:exon5:c.C964T:p.P322S
2	204000661	A	C	NBEAL1	NM_001114132	NBEAL1:NM_001114132:exon27:c.A3988C:p.S1330R
2	207171216	A	G	ZDBF2	NM_001285549,NM_020923	ZDBF2:NM_020923:exon5:c.A1964G:p.D655G,ZDBF2:NM_001285549:exon

						7:c.A1958G:p.D653G
2	207625630	A	G	MDH1B	NM_001039845,NM_001282940	MDH1B:NM_001039845:exon2:c.T130C:p.W44R,MDH1B:NM_001282940:exon2:c.T130C:p.W44R
2	209108301	T	C	IDH1	NM_001282386,NM_001282387,NM_005896	IDH1:NM_001282386:exon6:c.A548G:p.Y183C,IDH1:NM_001282387:exon6:c.A548G:p.Y183C,IDH1:NM_005896:exon6:c.A548G:p.Y183C
2	211456637	A	G	CPS1	NM_001122633,NM_001875	CPS1:NM_001875:exon10:c.A1030G:p.T344A,CPS1:NM_001122633:exon11:c.A1048G:p.T350A
2	215914429	T	G	ABCA12	NM_173076	ABCA12:NM_173076:exon6:c.A614C:p.N205T
2	219290468	T	G	VIL1	NM_007127	VIL1:NM_007127:exon4:c.T281G:p.V94G
2	220500493	C	T	SLC4A3	NM_005070,NM_201574	SLC4A3:NM_005070:exon14:c.C2071T:p.R691C,SLC4A3:NM_201574:exon14:c.C2152T:p.R718C
2	233273011	C	G	ALPPL2	NM_031313	ALPPL2:NM_031313:exon6:c.C683G:p.P228R
2	233735070	C	G	C2orf82	NM_206895	C2orf82:NM_206895:exon1:c.C19G:p.L7V
2	242716387	G	A	GAL3ST2	NM_022134	GAL3ST2:NM_022134:exon1:c.G17A:p.G6D
3	8661567	C	G	SSUH2	NM_001256748,NM_001256749,NM_015931	SSUH2:NM_001256748:exon12:c.G1115C:p.C372S,SSUH2:NM_001256749:exon12:c.G896C:p.C299S,SSUH2:NM_015931:exon12:c.G1049C:p.C350S
3	9787553	C	T	BRPF1	NM_001003694,NM_004634	BRPF1:NM_001003694:exon12:c.C3263T:p.P1088L,BRPF1:NM_004634:exon12:c.C3245T:p.P1082L
3	10401576	C	T	ATP2B2	NM_001001331,NM_001683	ATP2B2:NM_001683:exon10:c.G1756A:p.V586M,ATP2B2:NM_001001331:exon13:c.G1891A:p.V631M
3	12046131	A	C	SYN2	NM_003178,NM_133625	UNKNOWN
3	12187229	C	T	SYN2	NM_003178,NM_133625	UNKNOWN
3	14712693	G	A	CCDC174	NM_016474	CCDC174:NM_016474:exon11:c.G1396A:p.V466M
3	17052327	C	T	PLCL2	NM_001144382,NM_015184	PLCL2:NM_015184:exon2:c.C1111T:p.R371C
3	27203969	C	T	NEK10	NM_001031741,NM_001304384,NM_152534	NEK10:NM_001031741:exon10:c.G929A:p.R310Q,NEK10:NM_001304384:exon10:c.G929A:p.R310Q,NEK10:NM_152534:exon32:c.G2993A:p.R998Q
3	28378233	G	T	AZI2	NM_001134432,NM_001134433,NM_001271650,NM_022461	AZI2:NM_001134432:exon5:c.C583A:p.Q195K,AZI2:NM_001134433:exon5:c.C583A:p.Q195K,AZI2:NM_022461:exon5:c.C583A:p.Q195K,AZI2:NM_001271650:exon6:c.C247A:p.Q83K

3	33668484	T	C	CLASP2	NM_001207044,NM_015097	CLASP2:NM_001207044:exon4:c.A332G:p.N111S,CLASP2:NM_015097:exon10:c.A1034G:p.N345S
3	36897630	C	A	TRANK1	NM_014831	TRANK1:NM_014831:exon12:c.G3451T:p.V1151L
3	38752340	G	A	SCN10A	NM_001293306,NM_001293307,NM_006514	SCN10A:NM_001293307:exon22:c.C3844T:p.R1282W,SCN10A:NM_001293306:exon23:c.C4135T:p.R1379W,SCN10A:NM_006514:exon23:c.C4138T:p.R1380W
3	38798298	A	C	SCN10A	NM_001293306,NM_001293307,NM_006514	SCN10A:NM_001293306:exon9:c.T1157G:p.F386C,SCN10A:NM_001293307:exon9:c.T1157G:p.F386C,SCN10A:NM_006514:exon9:c.T1157G:p.F386C
3	39185834	G	A	CSRNP1	NM_033027	CSRNP1:NM_033027:exon4:c.C574T:p.R192W
3	42728107	C	G	KLHL40	NM_152393	KLHL40:NM_152393:exon1:c.C997G:p.P333A
3	48561230	C	G	PFKFB4	NM_004567	PFKFB4:NM_004567:exon11:c.G1126C:p.V376L
3	49155449	G	A	USP19	NM_001199160,NM_001199161,NM_001199162,NM_006677	USP19:NM_001199160:exon3:c.C229T:p.R77C,USP19:NM_001199161:exon3:c.C229T:p.R77C,USP19:NM_001199162:exon3:c.C229T:p.R77C,USP19:NM_006677:exon3:c.C229T:p.R77C
3	49736537	C	T	RNF123	NM_022064	RNF123:NM_022064:exon10:c.C763T:p.R255C
3	49751224	T	A	RNF123	NM_022064	RNF123:NM_022064:exon29:c.T2803A:p.S935T
3	49843379	A	G	UBA7	NM_003335	UBA7:NM_003335:exon23:c.T2860C:p.Y954H
3	51967583	C	G	RRP9	NM_004704	RRP9:NM_004704:exon15:c.G1367C:p.R456P
3	52553551	G	A	STAB1	NM_015136	STAB1:NM_015136:exon50:c.G5206A:p.G1736S
3	56763357	G	A	ARHGEF3	NM_001128615,NM_001128616,NM_001289698,NM_019555	ARHGEF3:NM_001128616:exon10:c.C1540T:p.R514C,ARHGEF3:NM_019555:exon10:c.C1522T:p.R508C,ARHGEF3:NM_001289698:exon11:c.C1540T:p.R514C,ARHGEF3:NM_001128615:exon13:c.C1618T:p.R540C
3	57494251	T	C	DNAH12	NM_001291661,NM_198564	DNAH12:NM_198564:exon7:c.A559G:p.N187D
3	72864491	G	A	SHQ1	NM_018130	.
3	75786670	G	A	ZNF717	NM_001128223,NM_001290208,NM_001290209	ZNF717:NM_001128223:exon5:c.C2104T:p.P702S,ZNF717:NM_001290208:exon5:c.C2104T:p.P702S,ZNF717:NM_001290209:exon5:c.C1954T:p.P652S
3	75786942	C	A	ZNF717	NM_001128223,NM_001290208,NM_001290209	ZNF717:NM_001128223:exon5:c.G1832T:p.R611I,ZNF717:NM_001290208:exon5:c.G1832T:p.R611I,ZNF717:NM_001290209:exon5:c.G1682T:p.R561I

3	75787405	C	T	ZNF717	NM_001128223,NM_001290208,NM_001290209	ZNF717:NM_001128223:exon5:c.G1369A:p.G457R,ZNF717:NM_001290208:exon5:c.G1369A:p.G457R,ZNF717:NM_001290209:exon5:c.G1219A:p.G407R
3	75787516	C	G	ZNF717	NM_001128223,NM_001290208,NM_001290209	ZNF717:NM_001128223:exon5:c.G1258C:p.E420Q,ZNF717:NM_001290208:exon5:c.G1258C:p.E420Q,ZNF717:NM_001290209:exon5:c.G1108C:p.E370Q
3	75787519	C	A	ZNF717	NM_001128223,NM_001290208,NM_001290209	ZNF717:NM_001128223:exon5:c.G1255T:p.G419W,ZNF717:NM_001290208:exon5:c.G1255T:p.G419W,ZNF717:NM_001290209:exon5:c.G1105T:p.G369W
3	75788130	C	A	ZNF717	NM_001128223,NM_001290208,NM_001290209	ZNF717:NM_001128223:exon5:c.G644T:p.G215V,ZNF717:NM_001290208:exon5:c.G644T:p.G215V,ZNF717:NM_001290209:exon5:c.G494T:p.G165V
3	75788158	G	C	ZNF717	NM_001128223,NM_001290208,NM_001290209	ZNF717:NM_001128223:exon5:c.C616G:p.L206V,ZNF717:NM_001290208:exon5:c.C616G:p.L206V,ZNF717:NM_001290209:exon5:c.C466G:p.L156V
3	97598601	A	G	CRYBG3	NM_153605	.
3	105243240	A	T	ALCAM	NM_001243280,NM_001243281,NM_001243283,NM_001627	ALCAM:NM_001243280:exon3:c.A282T:p.E94D,ALCAM:NM_001243281:exon3:c.A282T:p.E94D,ALCAM:NM_001243283:exon3:c.A282T:p.E94D,ALCAM:NM_001627:exon3:c.A282T:p.E94D
3	112546020	G	A	CD200R1L	NM_001008784,NM_001199215	CD200R1L:NM_001008784:exon4:c.C499T:p.P167S,CD200R1L:NM_001199215:exon5:c.C436T:p.P146S
3	113322844	G	A	SIDT1	NM_017699	SIDT1:NM_017699:exon13:c.G1301A:p.R434Q
3	113753763	C	G	KIAA1407	NM_020817	.
3	113755549	T	C	KIAA1407	NM_020817	KIAA1407:NM_020817:exon5:c.A500G:p.D167G
3	121209212	G	A	POLQ	NM_199420	POLQ:NM_199420:exon16:c.C2566T:p.R856C
3	124492558	G	C	ITGB5	NM_002213	ITGB5:NM_002213:exon11:c.C1895G:p.P632R
3	138738941	G	C	PRR23B	NM_001013650	PRR23B:NM_001013650:exon1:c.C563G:p.P188R
3	142215345	A	G	ATR	NM_001184	ATR:NM_001184:exon34:c.T5756C:p.M1919T
3	148545839	T	A	CPB1	NM_001871	CPB1:NM_001871:exon2:c.T122A:p.I41N
3	150931203	G	A	P2RY14	NM_001081455,NM_014879	P2RY14:NM_001081455:exon3:c.C902T:p.P301L,P2RY14:NM_014879:exon3:c.C902T:p.P301L
3	151545958	T	C	AADAC	NM_001086	AADAC:NM_001086:exon5:c.T1198C:p.X400Q

3	155267674	C	G	PLCH1	NM_001130960,NM_001130961,NM_014996	PLCH1:NM_001130960:exon9:c.G1228C:p.G410R,PLCH1:NM_001130961:exon9:c.G1228C:p.G410R,PLCH1:NM_014996:exon9:c.G1174C:p.G392R
3	155838607	C	G	KCNAB1	NM_172160	KCNAB1:NM_172160:exon1:c.C207G:p.N69K
3	169485590	T	C	ACTRT3	NM_032487	ACTRT3:NM_032487:exon2:c.A749G:p.Q250R
3	170584249	C	A	RPL22L1	NM_001099645	RPL22L1:NM_001099645:exon4:c.G289T:p.V97F
3	171330183	C	T	PLD1	NM_001130081,NM_002662	PLD1:NM_001130081:exon24:c.G2654A:p.R885H,PLD1:NM_002662:exon25:c.G2768A:p.R923H
3	179439202	C	T	USP13	NM_003940	USP13:NM_003940:exon8:c.C913T:p.L305F
3	180327501	C	T	TTC14	NM_001288582,NM_133462	TTC14:NM_001288582:exon12:c.C1484T:p.S495F,TTC14:NM_133462:exon12:c.C1484T:p.S495F
3	190374095	G	A	IL1RAP	NM_001167931	IL1RAP:NM_001167931:exon12:c.G1763A:p.R588K
3	191179177	T	G	PYDC2	NM_001083308	PYDC2:NM_001083308:exon1:c.T226G:p.F76V
3	193332805	T	C	OPA1	NM_015560,NM_130831,NM_130832,NM_130833,NM_130834,NM_130835,NM_130836,NM_130837	OPA1:NM_015560:exon2:c.T326C:p.V109A,OPA1:NM_130831:exon2:c.T326C:p.V109A,OPA1:NM_130832:exon2:c.T326C:p.V109A,OPA1:NM_130833:exon2:c.T326C:p.V109A,OPA1:NM_130834:exon2:c.T326C:p.V109A,OPA1:NM_130835:exon2:c.T326C:p.V109A,OPA1:NM_130836:exon2:c.T326C:p.V109A,OPA1:NM_130837:exon2:c.T326C:p.V109A
3	195456561	C	G	MUC20	NM_001282506,NM_001291833,NM_020790,NM_152673	MUC20:NM_001282506:exon3:c.C2012G:p.S671C,MUC20:NM_001291833:exon5:c.C1499G:p.S500C,MUC20:NM_020790:exon5:c.C1556G:p.S519C,MUC20:NM_152673:exon5:c.C1499G:p.S500C
3	195935382	T	C	ZDHHC19	NM_001039617	ZDHHC19:NM_001039617:exon4:c.A458G:p.N153S
4	2932856	G	A	MFSD10	NM_001120,NM_001146069	MFSD10:NM_001120:exon11:c.C1184T:p.T395M,MFSD10:NM_001146069:exon12:c.C1184T:p.T395M
4	2993963	G	C	GRK4	NM_001004056,NM_001004057,NM_005307,NM_182982	GRK4:NM_001004056:exon3:c.G187C:p.D63H,GRK4:NM_005307:exon3:c.G187C:p.D63H,GRK4:NM_001004057:exon4:c.G283C:p.D95H,GRK4:NM_182982:exon4:c.G283C:p.D95H
4	3516528	C	T	LRPAP1	NM_002337	LRPAP1:NM_002337:exon7:c.G962A:p.R321H
4	9922071	G	A	SLC2A9	NM_001001290,NM_020041	SLC2A9:NM_020041:exon7:c.C940T:p.R314C,SLC2A9:NM_001001290:exon8:c.C853T:p.R285C
4	15504497	G	A	CC2D2A	NM_001080522	CC2D2A:NM_001080522:exon7:c.G389A:p.R130H

4	17492351	G	A	QDPR	NM_000320	QDPR:NM_000320:exon6:c.C563T:p.P188L
4	38800152	A	G	TLR1	NM_003263	TLR1:NM_003263:exon4:c.T301C:p.S101P
4	38972741	C	G	TMEM156	NM_001303228,NM_024943	TMEM156:NM_001303228:exon6:c.G837C:p.R279S,TMEM156:NM_024943:exon6:c.G840C:p.R280S
4	39219646	G	A	WDR19	NM_025132	WDR19:NM_025132:exon14:c.G1400A:p.R467Q
4	39512385	T	C	UGDH	NM_001184701,NM_003359	UGDH:NM_001184701:exon3:c.A70G:p.N24D,UGDH:NM_003359:exon4:c.A361G:p.N121D
4	41673609	C	T	LIMCH1	NM_001112717,NM_001112718,NM_001112719,NM_001112720,NM_001289122,NM_001289124,NM_014988	LIMCH1:NM_001112720:exon11:c.C1783T:p.P595S,LIMCH1:NM_001112719:exon12:c.C1819T:p.P607S,LIMCH1:NM_001112717:exon17:c.C2281T:p.P761S,LIMCH1:NM_001112718:exon17:c.C2281T:p.P761S,LIMCH1:NM_001289122:exon17:c.C2320T:p.P774S,LIMCH1:NM_001289124:exon17:c.C1843T:p.P615S,LIMCH1:NM_014988:exon17:c.C2281T:p.P761S
4	47408896	G	A	GABRB1	NM_000812	GABRB1:NM_000812:exon8:c.G1033A:p.D345N
4	52942986	G	C	SPATA18	NM_001297608,NM_145263	SPATA18:NM_001297608:exon6:c.G704C:p.R235P,SPATA18:NM_145263:exon7:c.G800C:p.R267P
4	54294293	C	G	FIP1L1	NM_001134937,NM_001134938,NM_030917	FIP1L1:NM_001134938:exon10:c.C895G:p.P299A,FIP1L1:NM_001134937:exon12:c.C1072G:p.P358A,FIP1L1:NM_030917:exon13:c.C1117G:p.P373A
4	89020503	A	G	ABCG2	NM_001257386,NM_004827	ABCG2:NM_001257386:exon12:c.T1465C:p.F489L,ABCG2:NM_004827:exon12:c.T1465C:p.F489L
4	103451068	A	G	NFKB1	NM_001165412,NM_003998	NFKB1:NM_001165412:exon3:c.A115G:p.T39A,NFKB1:NM_003998:exon3:c.A115G:p.T39A
4	114208830	C	G	ANK2	NM_001127493,NM_001148,NM_020977	ANK2:NM_001148:exon19:c.C2149G:p.H717D,ANK2:NM_020977:exon19:c.C2149G:p.H717D,ANK2:NM_001127493:exon20:c.C2086G:p.H696D
4	120057709	A	C	MYOZ2	NM_016599	MYOZ2:NM_016599:exon2:c.A29C:p.Q10P
4	128904204	C	T	C4orf29	NM_001039717	.
4	134071773	G	A	PCDH10	NM_020815,NM_032961	PCDH10:NM_020815:exon1:c.G478A:p.E160K,PCDH10:NM_032961:exon1:c.G478A:p.E160K
4	140454313	G	A	SETD7	NM_030648	.
4	155486997	A	T	FGB	NM_001184741,NM_005141	FGB:NM_001184741:exon2:c.A152T:p.K51M,FGB:NM_005141:exon2:c.A152T:p.K51M

4	156830079	T	G	TDO2	NM_005651	TDO2:NM_005651:exon5:c.T344G:p.M115R
4	164087555	C	A	NAF1	NM_001128931,NM_138386	NAF1:NM_001128931:exon1:c.G325T:p.D109Y,NAF1:NM_138386:exon1:c.G325T:p.D109Y
4	187086544	C	T	FAM149A	NM_001006655,NM_015398	FAM149A:NM_001006655:exon10:c.C1090T:p.R364W,FAM149A:NM_015398:exon11:c.C1090T:p.R364W
4	188924533	C	T	ZFP42	NM_001304358,NM_174900	ZFP42:NM_001304358:exon3:c.C572T:p.P191L,ZFP42:NM_174900:exon4:c.C572T:p.P191L
5	54941697	T	A	SLC38A9	NM_001258286,NM_001258287,NM_001282429,NM_173514	SLC38A9:NM_001258286:exon10:c.A898T:p.T300S,SLC38A9:NM_001258287:exon10:c.A898T:p.T300S,SLC38A9:NM_001282429:exon11:c.A715T:p.T239S,SLC38A9:NM_173514:exon12:c.A1087T:p.T363S
5	60455940	T	C	SMIM15	NM_001048249	SMIM15:NM_001048249:exon3:c.A59G:p.Y20C
5	72864311	T	C	UTP15	NM_001284430,NM_032175	UTP15:NM_001284430:exon4:c.T193C:p.Y65H,UTP15:NM_032175:exon4:c.T250C:p.Y84H
5	78379596	G	T	BHMT2	NM_001178005,NM_017614	BHMT2:NM_001178005:exon6:c.G735T:p.R245S,BHMT2:NM_017614:exon7:c.G927T:p.R309S
5	78985833	G	A	CMYA5	NM_153610	CMYA5:NM_153610:exon1:c.G103A:p.E35K
5	79031546	T	C	CMYA5	NM_153610	CMYA5:NM_153610:exon2:c.T6958C:p.Y2320H
5	79465316	G	A	SERINC5	NM_001174071,NM_001174072,NM_178276	SERINC5:NM_001174071:exon6:c.C605T:p.T202M,SERINC5:NM_001174072:exon6:c.C605T:p.T202M,SERINC5:NM_178276:exon6:c.C605T:p.T202M
5	82816229	G	A	VCAN	NM_001164098,NM_004385	VCAN:NM_001164098:exon7:c.G2104A:p.E702K,VCAN:NM_004385:exon7:c.G2104A:p.E702K
5	82837060	G	C	VCAN	NM_001164097,NM_004385	VCAN:NM_001164097:exon7:c.G5277C:p.R1759S,VCAN:NM_004385:exon8:c.G8238C:p.R2746S
5	89979916	A	G	GPR98	NM_032119	ADGRV1:NM_032119:exon28:c.A6178G:p.I2060V
5	96430474	G	A	LIX1	NM_153234	LIX1:NM_153234:exon6:c.C827T:p.T276M
5	110438051	C	G	WDR36	NM_139281	WDR36:NM_139281:exon6:c.C718G:p.L240V
5	110439903	G	A	WDR36	NM_139281	WDR36:NM_139281:exon8:c.G926A:p.S309N
5	115394604	G	A	ARL14EPL	NM_001195581	ARL14EPL:NM_001195581:exon3:c.G419A:p.G140E
5	126383565	G	A	C5orf63	NM_001164479	C5orf63:NM_001164479:exon5:c.C214T:p.R72C
5	126860562	G	A	PRRC1	NM_001286808,NM_130809	PRRC1:NM_001286808:exon3:c.G443A:p.R148K,PRRC1:NM_130809:exon3

						:c.G443A:p.R148K
5	129019910	C	A	ADAMTS19	NM_133638	ADAMTS19:NM_133638:exon18:c.C2744A:p.P915H
5	137222565	T	A	MYOT	NM_001135940,NM_001300911,NM_006790	MYOT:NM_001135940:exon9:c.T651A:p.D217E,MYOT:NM_006790:exon9:c.T1203A:p.D401E,MYOT:NM_001300911:exon10:c.T858A:p.D286E
5	138855862	C	A	TMEM173	NM_198282	TMEM173:NM_198282:exon8:c.G1124T:p.R375L
5	140209553	T	C	PCDHA6	NM_018909,NM_031848	PCDHA6:NM_018909:exon1:c.T1877C:p.L626P,PCDHA6:NM_031848:exon1:c.T1877C:p.L626P
5	140250471	G	T	PCDHA11	NM_018902,NM_031861	PCDHA11:NM_018902:exon1:c.G1783T:p.A595S,PCDHA11:NM_031861:exon1:c.G1783T:p.A595S
5	140563561	C	A	PCDHB16	NM_020957	PCDHB16:NM_020957:exon1:c.C1427A:p.T476K
5	143539987	C	T	YIPF5	NM_001024947,NM_001271732,NM_030799	YIPF5:NM_001271732:exon5:c.G586A:p.V196I,YIPF5:NM_001024947:exon6:c.G748A:p.V250I,YIPF5:NM_030799:exon6:c.G748A:p.V250I
5	147820689	G	T	FBXO38	NM_001271723,NM_030793	FBXO38:NM_001271723:exon20:c.G2542T:p.V848F,FBXO38:NM_030793:exon21:c.G3052T:p.V1018F
5	149277984	A	G	PDE6A	NM_000440	PDE6A:NM_000440:exon10:c.T1349C:p.F450S
5	149929236	C	G	NDST1	NM_001301063,NM_001543	.
5	150056367	G	A	MYOZ3	NM_001122853,NM_133371	MYOZ3:NM_001122853:exon7:c.G686A:p.R229H,MYOZ3:NM_133371:exon7:c.G686A:p.R229H
5	156533787	T	C	HAVCR2	NM_032782	HAVCR2:NM_032782:exon2:c.A245G:p.Y82C
5	156917329	C	A	ADAM19	NM_033274	ADAM19:NM_033274:exon19:c.G2229T:p.R743S
5	167645928	G	A	TENM2	NM_001122679	TENM2:NM_001122679:exon23:c.G5005A:p.G1669S
5	170221295	G	C	GABRP	NM_001291985,NM_014211	GABRP:NM_001291985:exon4:c.G233C:p.S78T,GABRP:NM_014211:exon4:c.G233C:p.S78T
5	170883789	C	T	FGF18	NM_003862	FGF18:NM_003862:exon5:c.C604T:p.R202W
5	171480055	G	A	STK10	NM_005990	.
5	172659590	A	T	NKX2-5	NM_004387	NKX2-5:NM_004387:exon2:c.T957A:p.H319Q
5	175959412	C	T	RNF44	NM_014901	RNF44:NM_014901:exon2:c.G62A:p.R21Q
5	176793213	G	A	RGS14	NM_006480	RGS14:NM_006480:exon3:c.G103A:p.E35K

5	180551775	T	C	OR2V1	NM_001258283	OR2V1:NM_001258283:exon1:c.A530G:p.H177R
6	24828410	C	T	FAM65B	NM_001286445,NM_014722	FAM65B:NM_001286445:exon18:c.G2620A:p.G874S,FAM65B:NM_014722:exon19:c.G2683A:p.G895S
6	25813093	A	C	SLC17A1	NM_005074	SLC17A1:NM_005074:exon8:c.T863G:p.F288C
6	26285633	T	G	HIST1H4H	NM_003543	HIST1H4H:NM_003543:exon1:c.A95C:p.K32T
6	26446093	G	A	BTN3A3	NM_006994,NM_197974	BTN3A3:NM_006994:exon5:c.G595A:p.V199M,BTN3A3:NM_197974:exon5:c.G469A:p.V157M
6	27879448	G	A	OR2B2	NM_033057	OR2B2:NM_033057:exon1:c.C650T:p.S217L
6	29012478	A	C	OR2W1	NM_030903	OR2W1:NM_030903:exon1:c.T475G:p.L159V
6	29691576	G	C	HLA-F	NM_001098478,NM_001098479,NM_018950	HLA-F:NM_001098478:exon2:c.G206C:p.R69P,HLA-F:NM_001098479:exon2:c.G206C:p.R69P,HLA-F:NM_018950:exon2:c.G206C:p.R69P
6	30653737	G	A	PPP1R18	NM_001134870,NM_133471	PPP1R18:NM_133471:exon1:c.C59T:p.A20V,PPP1R18:NM_001134870:exon2:c.C59T:p.A20V
6	30670557	C	A	MDC1	NM_014641	MDC1:NM_014641:exon13:c.G5963T:p.S1988I
6	30673779	G	A	MDC1	NM_014641	MDC1:NM_014641:exon10:c.C3181T:p.L1061F
6	31597380	C	G	PRRC2A	NM_004638,NM_080686	PRRC2A:NM_004638:exon14:c.C2012G:p.S671C,PRRC2A:NM_080686:exon14:c.C2012G:p.S671C
6	31778948	C	T	HSPA1L	NM_005527	HSPA1L:NM_005527:exon2:c.G802A:p.A268T
6	32084867	C	A	ATF6B	NM_001136153,NM_004381	ATF6B:NM_001136153:exon15:c.G1623T:p.K541N,ATF6B:NM_004381:exon15:c.G1632T:p.K544N
6	32165239	G	C	NOTCH4	NM_004557	NOTCH4:NM_004557:exon27:c.C4889G:p.T1630S
6	32725625	T	C	HLA-DQB2	NM_001300790	HLA-DQB2:NM_001300790:exon4:c.A682G:p.S228G
6	32726803	C	T	HLA-DQB2	NM_001198858,NM_001300790	HLA-DQB2:NM_001198858:exon3:c.G470A:p.R157Q,HLA-DQB2:NM_001300790:exon3:c.G470A:p.R157Q
6	32827301	G	C	PSMB9	NM_002800	PSMB9:NM_002800:exon6:c.G652C:p.D218H
6	33236863	C	T	VPS52	NM_001289174,NM_001289175,NM_001289176,NM_022553	VPS52:NM_001289174:exon5:c.G275A:p.R92Q,VPS52:NM_001289175:exon6:c.G101A:p.R34Q,VPS52:NM_001289176:exon6:c.G101A:p.R34Q,VPS52:NM_022553:exon6:c.G476A:p.R159Q
6	33282824	C	T	ZBTB22	NM_001145338,NM_005453	ZBTB22:NM_001145338:exon2:c.G1870A:p.E624K,ZBTB22:NM_005453:exon2:c.G1870A:p.E624K

6	33411463	C	G	SYNGAP1	NM_006772	SYNGAP1:NM_006772:exon15:c.C3134G:p.A1045G
6	33694588	C	T	IP6K3	NM_001142883,NM_054111	IP6K3:NM_054111:exon4:c.G509A:p.S170N,IP6K3:NM_001142883:exon5:c.G509A:p.S170N
6	35209353	C	T	SCUBE3	NM_001303136,NM_152753	SCUBE3:NM_001303136:exon11:c.C1226T:p.S409L,SCUBE3:NM_152753:exon11:c.C1229T:p.S410L
6	41126454	T	A	TREM2	NM_001271821	TREM2:NM_001271821:exon4:c.A547T:p.S183C
6	41740541	G	C	FRS3	NM_006653	FRS3:NM_006653:exon5:c.C410G:p.P137R
6	47501393	G	T	CD2AP	NM_012120	CD2AP:NM_012120:exon3:c.G221T:p.R74M
6	52344468	C	G	EFHC1	NM_001172420,NM_018100	EFHC1:NM_018100:exon9:c.C1523G:p.T508R,EFHC1:NM_001172420:exon10:c.C1466G:p.T489R
6	54002243	C	G	MLIP	NM_001281746,NM_001281747	MLIP:NM_001281746:exon4:c.C1343G:p.P448R,MLIP:NM_001281747:exon4:c.C1376G:p.P459R
6	57393125	A	G	PRIM2	NM_000947	UNKNOWN
6	57393144	A	T	PRIM2	NM_000947	UNKNOWN
6	57467100	G	C	PRIM2	NM_000947	UNKNOWN
6	57467175	A	G	PRIM2	NM_000947	UNKNOWN
6	87968265	G	C	ZNF292	NM_015021	ZNF292:NM_015021:exon8:c.G4918C:p.A1640P
6	88331672	G	A	ORC3	NM_001197259,NM_012381,NM_181837	ORC3:NM_001197259:exon10:c.G697A:p.V233M,ORC3:NM_012381:exon11:c.G1126A:p.V376M,ORC3:NM_181837:exon11:c.G1126A:p.V376M
6	100062606	G	T	PRDM13	NM_021620	PRDM13:NM_021620:exon4:c.G2095T:p.V699F
6	106756282	T	C	ATG5	NM_001286106,NM_001286108,NM_004849	ATG5:NM_001286106:exon3:c.A193G:p.I65V,ATG5:NM_001286108:exon3:c.A193G:p.I65V,ATG5:NM_004849:exon3:c.A193G:p.I65V
6	109560552	G	A	LOC100996634	NM_001277339	LOC100996634:NM_001277339:exon2:c.G142A:p.E48K
6	123101544	C	T	FABP7	NM_001446	FABP7:NM_001446:exon2:c.C182T:p.T61M
6	145093086	G	A	UTRN	NM_007124	UTRN:NM_007124:exon58:c.G8539A:p.E2847K
6	152621819	C	A	SYNE1	NM_033071,NM_182961	SYNE1:NM_033071:exon92:c.G17426T:p.R5809L,SYNE1:NM_182961:exon93:c.G17639T:p.R5880L
6	152720820	C	T	SYNE1	NM_033071,NM_182961	SYNE1:NM_033071:exon48:c.G7189A:p.E2397K,SYNE1:NM_182961:exon4

						8:c.G7168A:p.E2390K
6	158359726	A	G	SNX9	NM_016224	SNX9:NM_016224:exon16:c.A1595G:p.Q532R
6	160328358	C	T	MAS1	NM_002377	MAS1:NM_002377:exon1:c.C371T:p.T124M
6	161807897	G	A	PARK2	NM_004562,NM_013987,NM_013988	PARK2:NM_013988:exon7:c.C649T:p.R217W,PARK2:NM_013987:exon9:c.C1012T:p.R338W,PARK2:NM_004562:exon10:c.C1096T:p.R366W
7	1786604	A	T	ELFN1	NM_001128636	ELFN1:NM_001128636:exon2:c.A2372T:p.E791V
7	4050669	C	T	SDK1	NM_152744	SDK1:NM_152744:exon15:c.C2203T:p.P735S
7	4272931	G	A	SDK1	NM_001079653,NM_152744	SDK1:NM_001079653:exon15:c.G1333A:p.A445T,SDK1:NM_152744:exon41:c.G5872A:p.A1958T
7	11582681	T	C	THSD7A	NM_015204	THSD7A:NM_015204:exon5:c.A1517G:p.H506R
7	12666254	T	C	SCIN	NM_001112706,NM_033128	SCIN:NM_033128:exon6:c.T286C:p.F96L,SCIN:NM_001112706:exon8:c.T1027C:p.F343L
7	16255722	A	G	ISPD	NM_001101417,NM_001101426	ISPD:NM_001101417:exon8:c.T1070C:p.L357S,ISPD:NM_001101426:exon9:c.T1220C:p.L407S
7	21892164	C	T	DNAH11	NM_001277115	DNAH11:NM_001277115:exon67:c.C10976T:p.A3659V
7	27689231	G	A	HIBADH	NM_152740	HIBADH:NM_152740:exon2:c.C113T:p.S38L
7	29070342	C	T	CPVL	NM_019029,NM_031311	CPVL:NM_019029:exon12:c.G1171A:p.V391M,CPVL:NM_031311:exon12:c.G1171A:p.V391M
7	29943929	C	T	WIPF3	NM_001080529	WIPF3:NM_001080529:exon8:c.C1379T:p.A460V
7	36671667	C	T	AOAH	NM_001177506,NM_001177507,NM_001637	AOAH:NM_001177507:exon5:c.G400A:p.A134T,AOAH:NM_001637:exon6:c.G496A:p.A166T
7	40234653	A	C	SUGCT	NM_001193311,NM_001193312,NM_001193313,NM_024728	SUGCT:NM_001193311:exon6:c.A499C:p.I167L,SUGCT:NM_001193312:exon6:c.A499C:p.I167L,SUGCT:NM_001193313:exon6:c.A499C:p.I167L,SUGCT:NM_024728:exon6:c.A388C:p.I130L
7	44113399	C	T	POLM	NM_001284330,NM_001284331,NM_013284	POLM:NM_001284330:exon7:c.G1057A:p.G353S,POLM:NM_001284331:exon8:c.G1186A:p.G396S,POLM:NM_013284:exon9:c.G1297A:p.G433S
7	44561801	T	C	NPC1L1	NM_001101648,NM_013389	NPC1L1:NM_001101648:exon11:c.A2678G:p.Y893C,NPC1L1:NM_013389:exon11:c.A2678G:p.Y893C
7	70228072	C	T	AUTS2	NM_001127231,NM_015570	AUTS2:NM_001127231:exon7:c.C959T:p.P320L,AUTS2:NM_015570:exon7:c.C959T:p.P320L

7	73151600	C	T	ABHD11	NM_148912,NM_148913	ABHD11:NM_148912:exon4:c.G584A:p.R195H,ABHD11:NM_148913:exon4:c.G563A:p.R188H
7	73245622	C	T	CLDN4	NM_001305	CLDN4:NM_001305:exon1:c.C91T:p.R31C
7	80141152	C	T	GNAT3	NM_001102386	GNAT3:NM_001102386:exon1:c.G91A:p.A31T
7	87180124	T	C	ABCB1	NM_000927	ABCB1:NM_000927:exon11:c.A1030G:p.S344G
7	94228105	G	T	SGCE	NM_001099400,NM_001099401,NM_001301139,NM_003919	SGCE:NM_001099400:exon8:c.C1208A:p.P403Q,SGCE:NM_001301139:exon8:c.C1112A:p.P371Q,SGCE:NM_001099401:exon9:c.C1235A:p.P412Q,SGCE:NM_003919:exon9:c.C1235A:p.P412Q
7	99704497	C	T	AP4M1	NM_004722	AP4M1:NM_004722:exon15:c.C1354T:p.R452W
7	99755917	C	G	C7orf43	NM_018275	C7orf43:NM_018275:exon1:c.G206C:p.R69T
7	100691388	T	C	MUC17	NM_001040105	MUC17:NM_001040105:exon4:c.T12527C:p.I4176T
7	102724261	C	G	ARMC10	NM_001161009,NM_001161010,NM_001161011,NM_001161012,NM_001161013,NM_031905	ARMC10:NM_001161009:exon2:c.C272G:p.A91G,ARMC10:NM_001161011:exon2:c.C272G:p.A91G,ARMC10:NM_001161012:exon2:c.C272G:p.A91G,ARMC10:NM_001161013:exon2:c.C272G:p.A91G,ARMC10:NM_001161010:exon3:c.C377G:p.A126G,ARMC10:NM_031905:exon3:c.C377G:p.A126G
7	103180699	C	T	RELN	NM_005045,NM_173054	RELN:NM_005045:exon44:c.G6875A:p.R2292H,RELN:NM_173054:exon44:c.G6875A:p.R2292H
7	120776099	G	T	CPED1	NM_001105533,NM_024913	CPED1:NM_001105533:exon13:c.G1654T:p.V552F,CPED1:NM_024913:exon14:c.G1654T:p.V552F
7	121624114	A	C	PTPRZ1	NM_001206838,NM_001206839,NM_002851	PTPRZ1:NM_001206838:exon8:c.A871C:p.K291Q,PTPRZ1:NM_001206839:exon8:c.A871C:p.K291Q,PTPRZ1:NM_002851:exon8:c.A871C:p.K291Q
7	122769473	A	G	SLC13A1	NM_022444	SLC13A1:NM_022444:exon9:c.T995C:p.V332A
7	128531540	G	C	KCP	NM_001135914,NM_199349	UNKNOWN
7	139715641	G	A	TBXAS1	NM_001061,NM_001130966,NM_001166253,NM_001166254,NM_030984	TBXAS1:NM_001061:exon11:c.G1348A:p.E450K,TBXAS1:NM_030984:exon11:c.G1348A:p.E450K,TBXAS1:NM_001166253:exon12:c.G1486A:p.E496K,TBXAS1:NM_001166254:exon13:c.G1144A:p.E382K,TBXAS1:NM_001130966:exon15:c.G1348A:p.E450K
7	141635690	G	T	CLEC5A	NM_001301167,NM_013252	CLEC5A:NM_001301167:exon4:c.C200A:p.S67X,CLEC5A:NM_013252:exon5:c.C269A:p.S90X
7	141889296	C	G	LOC93432	NM_001293626	LOC93432:NM_001293626:exon37:c.C4337G:p.P1446R

7	151797901	G	A	GALNT11	NM_001304514,NM_022087	GALNT11:NM_001304514:exon2:c.G71A:p.R24H,GALNT11:NM_022087:exon3:c.G314A:p.R105H
7	151845702	G	A	KMT2C	NM_170606	KMT2C:NM_170606:exon52:c.C13310T:p.T4437M
7	151945007	C	T	KMT2C	NM_170606	KMT2C:NM_170606:exon14:c.G2512A:p.G838S
7	151945204	G	A	KMT2C	NM_170606	KMT2C:NM_170606:exon14:c.C2315T:p.S772L
7	152357858	G	A	XRCC2	NM_005431	XRCC2:NM_005431:exon2:c.C49T:p.R17X
7	155251166	G	C	EN2	NM_001427	EN2:NM_001427:exon1:c.G94C:p.G32R
8	6728273	T	C	DEFB1	NM_005218	DEFB1:NM_005218:exon2:c.A137G:p.Y46C
8	17942229	C	T	ASAH1	NM_001127505,NM_004315	ASAH1:NM_001127505:exon1:c.G82A:p.E28K,ASAH1:NM_004315:exon1:c.G82A:p.E28K
8	21769976	C	T	DOK2	NM_003974	DOK2:NM_003974:exon2:c.G109A:p.A37T
8	21857150	T	G	XPO7	NM_015024	XPO7:NM_015024:exon24:c.T2747G:p.I916S
8	22451419	C	T	PDLIM2	NM_021630	PDLIM2:NM_021630:exon10:c.C1805T:p.A602V
8	22473725	G	C	CCAR2	NM_021174	CCAR2:NM_021174:exon14:c.G1809C:p.Q603H
8	24771405	T	A	NEFM	NM_005382	NEFM:NM_005382:exon1:c.T99A:p.S33R
8	27516969	G	A	SCARA3	NM_016240,NM_182826	SCARA3:NM_016240:exon5:c.G1282A:p.V428I,SCARA3:NM_182826:exon5:c.G1282A:p.V428I
8	28013555	A	G	ELP3	NM_001284220,NM_001284222,NM_001284224,NM_001284225,NM_001284226,NM_018091	.
8	33449695	G	C	DUSP26	NM_001305115,NM_001305116,NM_024025	DUSP26:NM_001305116:exon3:c.C472G:p.R158G,DUSP26:NM_001305115:exon4:c.C472G:p.R158G,DUSP26:NM_024025:exon4:c.C472G:p.R158G
8	39644572	T	C	ADAM2	NM_001278113,NM_001278114,NM_001464	ADAM2:NM_001278113:exon9:c.A755G:p.Y252C,ADAM2:NM_001278114:exon10:c.A812G:p.Y271C,ADAM2:NM_001464:exon10:c.A812G:p.Y271C
8	41399354	G	A	GINS4	NM_032336	GINS4:NM_032336:exon7:c.G511A:p.V171M
8	42611002	T	C	CHRNA6	NM_001199279,NM_004198	CHRNA6:NM_001199279:exon4:c.A1295G:p.N432S,CHRNA6:NM_004198:exon5:c.A1340G:p.N447S
8	59407065	C	T	CYP7A1	NM_000780	CYP7A1:NM_000780:exon4:c.G1039A:p.D347N

8	68075995	C	T	CSPP1	NM_001291339,NM_024790	CSPP1:NM_001291339:exon18:c.C1622T:p.P541L,CSPP1:NM_024790:exon21:c.C2657T:p.P886L
8	77775961	G	T	ZFHX4	NM_024721	ZFHX4:NM_024721:exon11:c.G10011T:p.Q3337H
8	94805495	C	T	TMEM67	NM_001142301,NM_153704	TMEM67:NM_153704:exon16:c.C1645T:p.R549C,TMEM67:NM_001142301:exon17:c.C1402T:p.R468C
8	103842137	G	A	AZIN1	NM_001301668,NM_015878,NM_148174	AZIN1:NM_001301668:exon10:c.C932T:p.A311V,AZIN1:NM_148174:exon10:c.C932T:p.A311V,AZIN1:NM_015878:exon11:c.C932T:p.A311V
8	104928774	G	T	RIMS2	NM_001100117,NM_001282881,NM_014677	.
8	107773623	T	C	ABRA	NM_139166	ABRA:NM_139166:exon2:c.A788G:p.N263S
8	121259936	C	T	COL14A1	NM_021110	COL14A1:NM_021110:exon21:c.C2564T:p.P855L
8	130760802	G	A	GSDMC	NM_031415	GSDMC:NM_031415:exon14:c.C1472T:p.P491L
8	135545203	C	T	ZFAT	NM_001029939,NM_001167583,NM_001174157,NM_001174158,NM_001289394,NM_020863	ZFAT:NM_001174157:exon11:c.G2803A:p.A935T,ZFAT:NM_001167583:exon12:c.G2953A:p.A985T,ZFAT:NM_001174158:exon12:c.G2953A:p.A985T,ZFAT:NM_020863:exon12:c.G2989A:p.A997T,ZFAT:NM_001029939:exon13:c.G2953A:p.A985T,ZFAT:NM_001289394:exon13:c.G2953A:p.A985T
8	140744344	G	A	TRAPPC9	NM_001160372,NM_031466	TRAPPC9:NM_001160372:exon22:c.C3157T:p.R1053C,TRAPPC9:NM_031466:exon22:c.C3451T:p.R1151C
8	142185385	C	G	DENND3	NM_014957	DENND3:NM_014957:exon14:c.C2122G:p.L708V
8	145106273	T	A	OPLAH	NM_017570	OPLAH:NM_017570:exon28:c.A3821T:p.E1274V
8	145154282	G	A	SHARPIN	NM_030974	SHARPIN:NM_030974:exon6:c.C820T:p.R274W
9	712757	C	T	KANK1	NM_001256876,NM_001256877,NM_015158,NM_153186	KANK1:NM_153186:exon2:c.C1517T:p.A506V,KANK1:NM_015158:exon3:c.C1991T:p.A664V,KANK1:NM_001256877:exon4:c.C1991T:p.A664V,KANK1:NM_001256876:exon7:c.C1991T:p.A664V
9	4618086	T	C	SPATA6L	NM_001039395	SPATA6L:NM_001039395:exon8:c.A658G:p.I220V
9	5044432	G	A	JAK2	NM_004972	JAK2:NM_004972:exon5:c.G380A:p.G127D
9	17298289	C	T	CNTLN	NM_001114395,NM_017738	CNTLN:NM_001114395:exon7:c.C1085T:p.T362I,CNTLN:NM_017738:exon7:c.C1085T:p.T362I
9	33941702	C	T	UBAP2	NM_001282529,NM_018449	UBAP2:NM_001282529:exon12:c.G1073A:p.R358K,UBAP2:NM_018449:exon16:c.G1874A:p.R625K

9	35377549	G	A	UNC13B	NM_006377	UNC13B:NM_006377:exon15:c.G1673A:p.R558Q
9	35389944	G	C	UNC13B	NM_006377	UNC13B:NM_006377:exon24:c.G2949C:p.K983N
9	35906604	A	C	HRCT1	NM_001039792	HRCT1:NM_001039792:exon1:c.A320C:p.H107P
9	37801374	G	A	DCAF10	NM_024345	DCAF10:NM_024345:exon1:c.G511A:p.V171I
9	72874183	A	C	SMC5	NM_015110	.
9	86504035	T	A	KIF27	NM_001271927,NM_001271928,NM_017576	KIF27:NM_001271927:exon7:c.A1943T:p.D648V,KIF27:NM_001271928:exon7:c.A1943T:p.D648V,KIF27:NM_017576:exon7:c.A1943T:p.D648V
9	88960693	C	T	ZCCHC6	NM_001185059,NM_001185074,NM_024617	ZCCHC6:NM_001185059:exon4:c.G710A:p.R237Q,ZCCHC6:NM_001185074:exon4:c.G710A:p.R237Q,ZCCHC6:NM_024617:exon4:c.G710A:p.R237Q
9	91616590	G	T	S1PR3	NM_005226	S1PR3:NM_005226:exon2:c.G475T:p.G159W
9	94797146	G	A	SPTLC1	NM_001281303,NM_006415	SPTLC1:NM_001281303:exon14:c.C1274T:p.A425V,SPTLC1:NM_006415:exon14:c.C1274T:p.A425V
9	95055715	A	T	IARS	NM_013417	.
9	95063881	C	T	NOL8	NM_001256394,NM_017948	NOL8:NM_017948:exon13:c.G3127A:p.G1043R,NOL8:NM_001256394:exon14:c.G2923A:p.G975R
9	96289514	A	T	FAM120A	NM_001286722,NM_001286723,NM_001286724,NM_014612	FAM120A:NM_001286722:exon8:c.A1493T:p.D498V,FAM120A:NM_001286723:exon8:c.A1496T:p.D499V,FAM120A:NM_001286724:exon8:c.A1496T:p.D499V,FAM120A:NM_014612:exon8:c.A1496T:p.D499V
9	97333800	C	A	FBP2	NM_003837	FBP2:NM_003837:exon4:c.G511T:p.A171S
9	99250506	C	T	HABP4	NM_014282	HABP4:NM_014282:exon7:c.C1135T:p.R379W
9	101536290	G	T	ANKS6	NM_173551	ANKS6:NM_173551:exon9:c.C1690A:p.P564T
9	101594151	G	A	GALNT12	NM_024642	GALNT12:NM_024642:exon4:c.G829A:p.G277S
9	107288586	T	C	OR13C4	NM_001001919	OR13C4:NM_001001919:exon1:c.A905G:p.D302G
9	107298322	A	G	OR13C3	NM_001001961	OR13C3:NM_001001961:exon1:c.T773C:p.M258T
9	113013711	C	T	TXN	NM_001244938,NM_003329	TXN:NM_001244938:exon2:c.G56A:p.G19D,TXN:NM_003329:exon2:c.G56A:p.G19D
9	113547891	G	A	MUSK	NM_001166280,NM_001166281,NM_005592	MUSK:NM_001166281:exon10:c.G1383A:p.M461I,MUSK:NM_001166280:exon11:c.G1413A:p.M471I,MUSK:NM_005592:exon12:c.G1671A:p.M557I
9	116812116	A	C	ZNF618	NM_133374	ZNF618:NM_133374:exon14:c.A2255C:p.D752A

9	116812124	C	T	ZNF618	NM_133374	ZNF618:NM_133374:exon14:c.C2263T:p.P755S
9	117136363	G	A	AKNA	NM_030767	.
9	117838677	G	A	TNC	NM_002160	TNC:NM_002160:exon8:c.C2852T:p.T951I
9	119249734	G	A	ASTN2	NM_001184734,NM_014010,NM_198186,NM_198187,NM_198188	ASTN2:NM_001184734:exon5:c.C557T:p.T186I,ASTN2:NM_198186:exon5:c.C704T:p.T235I,ASTN2:NM_198187:exon5:c.C557T:p.T186I,ASTN2:NM_198188:exon5:c.C557T:p.T186I,ASTN2:NM_014010:exon19:c.C3248T:p.T1083I
9	119976780	T	A	ASTN2	NM_014010	ASTN2:NM_014010:exon3:c.A872T:p.D291V
9	125145963	A	G	PTGS1	NM_000962,NM_001271164,NM_001271165,NM_001271166,NM_001271367,NM_001271368,NM_080591	PTGS1:NM_001271164:exon7:c.A794G:p.D265G,PTGS1:NM_001271165:exon7:c.A611G:p.D204G,PTGS1:NM_000962:exon8:c.A938G:p.D313G,PTGS1:NM_001271166:exon8:c.A611G:p.D204G,PTGS1:NM_001271367:exon8:c.A611G:p.D204G,PTGS1:NM_080591:exon8:c.A938G:p.D313G,PTGS1:NM_001271368:exon9:c.A863G:p.D288G
9	128001326	C	T	HSPA5	NM_005347	HSPA5:NM_005347:exon5:c.G890A:p.R297Q
9	130493004	T	G	TTC16	NM_144965	TTC16:NM_144965:exon14:c.T1942G:p.S648A
9	131072140	G	A	TRUB2	NM_015679	TRUB2:NM_015679:exon8:c.C685T:p.H229Y
9	133787235	C	T	FIBCD1	NM_001145106,NM_032843	FIBCD1:NM_032843:exon5:c.G890A:p.R297Q,FIBCD1:NM_001145106:exon6:c.G890A:p.R297Q
9	135117313	C	A	NTNG2	NM_032536	NTNG2:NM_032536:exon8:c.C1408A:p.L470M
9	136522275	G	A	DBH	NM_000787	DBH:NM_000787:exon11:c.G1646A:p.R549H
9	138657001	T	C	KCNT1	NM_001272003,NM_020822	KCNT1:NM_001272003:exon11:c.T1025C:p.M342T,KCNT1:NM_020822:exon12:c.T1160C:p.M387T
9	139749495	G	A	MAMDC4	NM_206920	MAMDC4:NM_206920:exon10:c.G1130A:p.R377H
9	140001194	G	A	MAN1B1	NM_016219	MAN1B1:NM_016219:exon10:c.G1499A:p.R500Q
9	140007915	C	T	DPP7	NM_013379	DPP7:NM_013379:exon5:c.G519A:p.M173I
9	140110727	C	T	NDOR1	NM_001144026,NM_001144027,NM_001144028,NM_014434	NDOR1:NM_001144027:exon13:c.C1529T:p.T510M
9	140323749	C	T	NOXA1	NM_001256067,NM_006647	NOXA1:NM_001256067:exon5:c.C526T:p.R176W,NOXA1:NM_006647:exon5:c.C526T:p.R176W
9	140446879	A	G	MRPL41	NM_032477	MRPL41:NM_032477:exon2:c.A346G:p.K116E

9	140777306	C	G	CACNA1B	NM_000718,NM_001243812	CACNA1B:NM_000718:exon3:c.C501G:p.N167K,CACNA1B:NM_001243812:exon3:c.C501G:p.N167K
10	1279729	G	A	ADARB2	NM_018702	ADARB2:NM_018702:exon6:c.C1420T:p.R474W
10	5437430	G	A	TUBAL3	NM_001171864,NM_024803	TUBAL3:NM_001171864:exon3:c.C136T:p.R46W,TUBAL3:NM_024803:exon3:c.C256T:p.R86W
10	7218090	C	T	SFMBT2	NM_001018039,NM_001029880	SFMBT2:NM_001018039:exon17:c.G1846A:p.V616I,SFMBT2:NM_001029880:exon17:c.G1846A:p.V616I
10	16990479	G	A	CUBN	NM_001081	CUBN:NM_001081:exon35:c.C5207T:p.S1736L
10	19678479	A	G	MALRD1	NM_001142308	MALRD1:NM_001142308:exon28:c.A4786G:p.M1596V
10	21435321	G	T	C10orf113	NM_001010896,NM_001177483	C10orf113:NM_001010896:exon1:c.C117A:p.N39K,C10orf113:NM_001177483:exon1:c.C117A:p.N39K
10	27035311	A	G	PDSS1	NM_014317	PDSS1:NM_014317:exon12:c.A1157G:p.E386G
10	29783908	A	G	SVIL	NM_003174,NM_021738	SVIL:NM_003174:exon18:c.T2498C:p.M833T,SVIL:NM_021738:exon20:c.T3776C:p.M1259T
10	29784072	G	C	SVIL	NM_003174,NM_021738	SVIL:NM_003174:exon17:c.C2425G:p.P809A,SVIL:NM_021738:exon19:c.C3703G:p.P1235A
10	29813481	G	A	SVIL	NM_003174,NM_021738	SVIL:NM_003174:exon12:c.C1228T:p.R410W,SVIL:NM_021738:exon14:c.C2506T:p.R836W
10	30315269	G	A	KIAA1462	NM_020848	KIAA1462:NM_020848:exon3:c.C3808T:p.R1270W
10	44053013	G	C	ZNF239	NM_001099282,NM_001099283,NM_001099284,NM_005674	ZNF239:NM_001099283:exon2:c.C515G:p.A172G,ZNF239:NM_005674:exon2:c.C515G:p.A172G,ZNF239:NM_001099284:exon3:c.C515G:p.A172G,ZNF239:NM_001099282:exon4:c.C515G:p.A172G
10	46998995	C	G	GPRIN2	NM_014696	GPRIN2:NM_014696:exon3:c.C115G:p.L39V
10	46999019	G	A	GPRIN2	NM_014696	GPRIN2:NM_014696:exon3:c.G139A:p.V47M
10	46999484	G	T	GPRIN2	NM_014696	GPRIN2:NM_014696:exon3:c.G604T:p.G202W
10	46999922	G	T	GPRIN2	NM_014696	GPRIN2:NM_014696:exon3:c.G1042T:p.V348L
10	47087078	G	T	NPY4R,LOC100996758	NM_001278794,NM_001278795,NM_005972	LOC100996758:NM_001278795:exon1:c.G295T:p.A99S,NPY4R:NM_001278794:exon2:c.G295T:p.A99S,NPY4R:NM_005972:exon3:c.G295T:p.A99S
10	47087501	C	T	NPY4R,LOC10099675	NM_001278794,NM_001278795,NM_005972	LOC100996758:NM_001278795:exon1:c.C718T:p.R240C,NPY4R:NM_001278794:exon2:c.C718T:p.R240C,NPY4R:NM_005972:exon3:c.C718T:p.R240C

				8		
10	48388228	C	T	RBP3	NM_002900	RBP3:NM_002900:exon1:c.G2650A:p.V884M
10	50669489	T	C	ERCC6	NM_000124	ERCC6:NM_000124:exon19:c.A3892G:p.R1298G
10	55944949	T	C	PCDH15	NM_001142763,NM_001142764,NM_001142765,NM_001142766,NM_001142767,NM_001142768,NM_001142769,NM_001142770,NM_001142771,NM_001142772,NM_001142773,NM_033056	PCDH15:NM_001142767:exon11:c.A1274G:p.Y425C,PCDH15:NM_001142768:exon11:c.A1319G:p.Y440C,PCDH15:NM_001142773:exon11:c.A1319G:p.Y440C,PCDH15:NM_001142764:exon12:c.A1385G:p.Y462C,PCDH15:NM_001142765:exon12:c.A1385G:p.Y462C,PCDH15:NM_001142766:exon12:c.A1385G:p.Y462C,PCDH15:NM_033056:exon12:c.A1385G:p.Y462C,PCDH15:NM_001142763:exon13:c.A1400G:p.Y467C
10	70404467	A	C	TET1	NM_030625	TET1:NM_030625:exon4:c.A1981C:p.N661H
10	70847918	T	C	SRGN	NM_002727	SRGN:NM_002727:exon1:c.T5C:p.M2T
10	70992549	G	A	HKDC1	NM_025130	HKDC1:NM_025130:exon3:c.G256A:p.G86R
10	72014877	G	A	NPFFR1	NM_022146	NPFFR1:NM_022146:exon4:c.C1129T:p.R377W
10	73472415	C	T	CDH23	NM_001171930,NM_022124	.
10	73492032	T	C	CDH23	NM_001171930,NM_022124	CDH23:NM_001171930:exon31:c.T4004C:p.V1335A,CDH23:NM_022124:exon31:c.T4004C:p.V1335A
10	74899468	T	A	ECD	NM_001135752,NM_001135753,NM_007265	ECD:NM_001135753:exon9:c.A1026T:p.E342D,ECD:NM_007265:exon10:c.A1155T:p.E385D,ECD:NM_001135752:exon11:c.A1254T:p.E418D
10	74999077	C	A	FAM149B1	NM_173348	FAM149B1:NM_173348:exon13:c.C1610A:p.T537K
10	76994918	G	A	COMTD1	NM_144589	COMTD1:NM_144589:exon4:c.C347T:p.S116F
10	85944516	G	T	C10orf99	NM_207373	C10orf99:NM_207373:exon3:c.G240T:p.Q80H
10	96121503	G	T	NOC3L	NM_022451	NOC3L:NM_022451:exon2:c.C136A:p.Q46K
10	97023725	C	A	PDLIM1	NM_020992	PDLIM1:NM_020992:exon4:c.G429T:p.Q143H
10	97200941	T	C	SORBS1	NM_001290294,NM_001290295,NM_015385,NM_024991	.
10	97919631	G	C	ZNF518A	NM_001278524,NM_001278525,NM_001278526,NM_014803	UNKNOWN

10	98080388	T	C	DNTT	NM_001017520,NM_004088	DNTT:NM_001017520:exon4:c.T521C:p.I174T,DNTT:NM_004088:exon4:c.T521C:p.I174T
10	99139505	A	T	RRP12	NM_001145114,NM_001284337,NM_015179	RRP12:NM_001284337:exon11:c.T1256A:p.L419Q,RRP12:NM_001145114:exon12:c.T1373A:p.L458Q,RRP12:NM_015179:exon14:c.T1556A:p.L519Q
10	101560288	C	T	ABCC2	NM_000392	ABCC2:NM_000392:exon9:c.C1177T:p.R393W
10	101668723	A	G	DNMBP	NM_015221	DNMBP:NM_015221:exon5:c.T2441C:p.M814T
10	102762659	G	A	LZTS2	NM_032429	LZTS2:NM_032429:exon2:c.G364A:p.G122S
10	103917272	A	G	NOLC1	NM_001284388,NM_001284389,NM_004741	NOLC1:NM_001284388:exon4:c.A401G:p.D134G,NOLC1:NM_001284389:exon4:c.A404G:p.D135G,NOLC1:NM_004741:exon4:c.A401G:p.D134G
10	105793739	T	C	COL17A1	NM_000494	COL17A1:NM_000494:exon52:c.A4120G:p.N1374D
10	115366006	G	T	NRAP	NM_001261463,NM_006175,NM_198060	NRAP:NM_006175:exon32:c.C3633A:p.H1211Q,NRAP:NM_001261463:exon33:c.C3738A:p.H1246Q,NRAP:NM_198060:exon33:c.C3738A:p.H1246Q
10	115529558	G	C	PLEKHS1	NM_001193434,NM_001193435,NM_024889,NM_182601	PLEKHS1:NM_001193435:exon5:c.G187C:p.G63R,PLEKHS1:NM_182601:exon6:c.G433C:p.G145R,PLEKHS1:NM_001193434:exon7:c.G187C:p.G63R,PLEKHS1:NM_024889:exon7:c.G451C:p.G151R
10	118386509	G	A	PNLIPRP2	NM_005396	UNKNOWN
10	121286832	C	T	RGS10	NM_001005339,NM_002925	RGS10:NM_001005339:exon2:c.G154A:p.V52M,RGS10:NM_002925:exon2:c.G112A:p.V38M
10	126519970	C	G	FAM175B	NM_032182	FAM175B:NM_032182:exon8:c.C716G:p.A239G
10	129911856	C	G	MKI67	NM_001145966,NM_002417	MKI67:NM_001145966:exon7:c.G411C:p.E137D,MKI67:NM_002417:exon8:c.G1491C:p.E497D
10	134013904	G	A	DPYSL4	NM_006426	DPYSL4:NM_006426:exon9:c.G856A:p.D286N
10	134650370	C	T	CFAP46	NM_001200049	CFAP46:NM_001200049:exon45:c.G6487A:p.E2163K
10	135342034	G	A	CYP2E1	NM_000773	CYP2E1:NM_000773:exon2:c.G227A:p.R76H
11	1213424	A	G	MUC5AC	NM_001304359	UNKNOWN
11	1213440	T	C	MUC5AC	NM_001304359	UNKNOWN
11	2436476	G	A	TRPM5	NM_014555	TRPM5:NM_014555:exon9:c.C1354T:p.R452W
11	4411623	C	T	TRIM21	NM_003141	TRIM21:NM_003141:exon2:c.G17A:p.R6H
11	5153821	C	T	OR52A5	NM_001005160	OR52A5:NM_001005160:exon1:c.G52A:p.G18R

11	5344737	T	A	OR51B2	NM_033180	OR51B2:NM_033180:exon1:c.A791T:p.K264M
11	5510553	C	T	OR52D1	NM_001005163	OR52D1:NM_001005163:exon1:c.C617T:p.A206V
11	6239315	G	A	FAM160A2	NM_001098794,NM_032127	FAM160A2:NM_001098794:exon9:c.C1501T:p.R501C,FAM160A2:NM_032127:exon9:c.C1543T:p.R515C
11	6530401	G	C	DNHD1	NM_144666,NM_173589	DNHD1:NM_173589:exon5:c.G1134C:p.K378N,DNHD1:NM_144666:exon6:c.G1134C:p.K378N
11	7689026	T	G	CYB5R2	NM_001302826,NM_001302827,NM_016229	CYB5R2:NM_001302827:exon6:c.A491C:p.Q164P,CYB5R2:NM_001302826:exon7:c.A491C:p.Q164P,CYB5R2:NM_016229:exon7:c.A491C:p.Q164P
11	8646450	T	G	TRIM66	NM_014818	TRIM66:NM_014818:exon11:c.A2201C:p.Q734P
11	8706353	T	G	RPL27A	NM_000990	RPL27A:NM_000990:exon4:c.T232G:p.L78V
11	17635225	A	C	OTOG	NM_001277269,NM_001292063	OTOG:NM_001277269:exon38:c.A6541C:p.T2181P,OTOG:NM_001292063:exon39:c.A6505C:p.T2169P
11	18956196	C	T	MRGPRX1	NM_147199	MRGPRX1:NM_147199:exon1:c.G136A:p.A46T
11	27016411	A	G	FIBIN	NM_203371	FIBIN:NM_203371:exon1:c.A338G:p.E113G
11	44076808	C	A	ACCSL	NM_001031854	ACCSL:NM_001031854:exon9:c.C1106A:p.S369Y
11	46354997	C	T	DGKZ	NM_201532	DGKZ:NM_201532:exon1:c.C172T:p.R58C
11	57256441	T	C	SLC43A1	NM_001198810,NM_003627	SLC43A1:NM_001198810:exon13:c.A1357G:p.T453A,SLC43A1:NM_003627:exon13:c.A1357G:p.T453A
11	62288061	C	T	AHNAK	NM_001620	AHNAK:NM_001620:exon5:c.G13828A:p.D4610N
11	62598564	G	A	STX5	NM_001244666,NM_003164	STX5:NM_001244666:exon2:c.C152T:p.P51L,STX5:NM_003164:exon2:c.C152T:p.P51L
11	63067061	C	T	SLC22A10	NM_001039752	SLC22A10:NM_001039752:exon6:c.C1030T:p.P344S
11	63586472	C	T	C11orf84	NM_138471	C11orf84:NM_138471:exon5:c.C932T:p.P311L
11	64575505	C	T	MEN1	NM_000244,NM_130799,NM_130800,NM_130801,NM_130802,NM_130803,NM_130804	MEN1:NM_000244:exon3:c.G527A:p.R176Q,MEN1:NM_130799:exon3:c.G512A:p.R171Q,MEN1:NM_130800:exon3:c.G527A:p.R176Q,MEN1:NM_130801:exon3:c.G527A:p.R176Q,MEN1:NM_130802:exon3:c.G527A:p.R176Q,MEN1:NM_130803:exon3:c.G527A:p.R176Q,MEN1:NM_130804:exon4:c.G527A:p.R176Q
11	66281952	G	A	BBS1	NM_024649	BBS1:NM_024649:exon4:c.G235A:p.E79K
11	68855341	A	G	TPCN2	NM_139075	.

11	70331960	G	T	SHANK2	NM_012309,NM_133266	SHANK2:NM_133266:exon10:c.C2674A:p.P892T
11	72418416	G	C	ARAP1	NM_001040118,NM_001135190,NM_015242	ARAP1:NM_001135190:exon10:c.C792G:p.F264L,ARAP1:NM_015242:exon10:c.C792G:p.F264L,ARAP1:NM_001040118:exon12:c.C1527G:p.F509L
11	74556109	C	T	XRRA1	NM_001270380,NM_001270381,NM_182969	.
11	76969468	G	A	GDPD4	NM_182833	GDPD4:NM_182833:exon10:c.C827T:p.S276L
11	82895905	A	G	PCF11	NM_015885	PCF11:NM_015885:exon16:c.A4637G:p.K1546R
11	94862502	A	G	ENDOD1	NM_015036	ENDOD1:NM_015036:exon2:c.A1262G:p.K421R
11	95568581	G	C	MTMR2	NM_001243571,NM_016156,NM_201278,NM_201281	MTMR2:NM_016156:exon15:c.C1805G:p.A602G,MTMR2:NM_201281:exon16:c.C1589G:p.A530G,MTMR2:NM_201278:exon17:c.C1589G:p.A530G,MTMR2:NM_001243571:exon18:c.C1589G:p.A530G
11	102248317	A	G	BIRC2	NM_001166,NM_001256163,NM_001256166	BIRC2:NM_001166:exon7:c.A1457G:p.N486S,BIRC2:NM_001256163:exon7:c.A1457G:p.N486S,BIRC2:NM_001256166:exon7:c.A1310G:p.N437S
11	103907858	G	C	DDI1	NM_001001711	DDI1:NM_001001711:exon1:c.G308C:p.G103A
11	111941175	G	T	PIH1D2	NM_001082619,NM_138789	PIH1D2:NM_001082619:exon5:c.C798A:p.D266E,PIH1D2:NM_138789:exon5:c.C798A:p.D266E
11	113846041	G	T	HTR3A	NM_000869,NM_213621	HTR3A:NM_000869:exon1:c.G12T:p.K4N,HTR3A:NM_213621:exon1:c.G12T:p.K4N
11	118185215	G	A	CD3E	NM_000733	.
11	118376737	A	G	KMT2A	NM_001197104,NM_005933	KMT2A:NM_001197104:exon27:c.A10130G:p.D3377G,KMT2A:NM_005933:exon27:c.A10121G:p.D3374G
11	118509676	G	A	PHLDB1	NM_001144758,NM_001144759,NM_015157	PHLDB1:NM_001144758:exon11:c.G2603A:p.R868H,PHLDB1:NM_001144759:exon11:c.G2603A:p.R868H,PHLDB1:NM_015157:exon12:c.G2603A:p.R868H
11	118518751	G	A	PHLDB1	NM_001144758,NM_001144759,NM_015157	PHLDB1:NM_001144759:exon17:c.G3472A:p.V1158I,PHLDB1:NM_001144758:exon18:c.G3613A:p.V1205I,PHLDB1:NM_015157:exon19:c.G3613A:p.V1205I
11	118532376	A	G	TREH	NM_007180	UNKNOWN
11	119045520	G	A	NLRX1	NM_001282143,NM_001282144,NM_001282358,NM_024618	NLRX1:NM_001282143:exon6:c.G1208A:p.R403H,NLRX1:NM_001282144:exon6:c.G1208A:p.R403H,NLRX1:NM_001282358:exon6:c.G1208A:p.R403

						H,NLRX1:NM_024618:exon6:c.G1208A:p.R403H
11	119243866	C	T	USP2	NM_004205	USP2:NM_004205:exon2:c.G325A:p.G109R
11	130109776	C	G	ZBTB44	NM_001301098,NM_001301099,NM_014155	ZBTB44:NM_001301098:exon3:c.G1034C:p.G345A,ZBTB44:NM_001301099:exon3:c.G1034C:p.G345A,ZBTB44:NM_014155:exon3:c.G1034C:p.G345A
11	130750661	G	A	SNX19	NM_001301089,NM_014758	SNX19:NM_001301089:exon9:c.C754T:p.R252C,SNX19:NM_014758:exon9:c.C2614T:p.R872C
12	6640531	A	G	NCAPD2	NM_014865	NCAPD2:NM_014865:exon32:c.A4159G:p.K1387E
12	6729676	A	G	LPAR5	NM_001142961,NM_020400	LPAR5:NM_001142961:exon2:c.T739C:p.F247L,LPAR5:NM_020400:exon2:c.T739C:p.F247L
12	6976687	G	A	TPI1	NM_001159287	TPI1:NM_001159287:exon1:c.G68A:p.R23Q
12	11139458	A	G	TAS2R50	NM_176890	TAS2R50:NM_176890:exon1:c.T2C:p.M1T
12	18891318	G	A	CAPZA3	NM_033328	CAPZA3:NM_033328:exon1:c.G116A:p.R39H
12	21036413	A	C	SLCO1B3	NM_019844	SLCO1B3:NM_019844:exon13:c.A1559C:p.H520P
12	21375289	C	T	SLCO1B1	NM_006446	SLCO1B1:NM_006446:exon13:c.C1738T:p.R580X
12	24048776	G	A	SOX5	NM_001261414,NM_001261415,NM_006940,NM_152989	SOX5:NM_001261415:exon2:c.C191T:p.T64M,SOX5:NM_006940:exon2:c.C221T:p.T74M,SOX5:NM_152989:exon5:c.C182T:p.T61M,SOX5:NM_001261414:exon6:c.C182T:p.T61M
12	32134413	G	A	KIAA1551	NM_018169	KIAA1551:NM_018169:exon4:c.G524A:p.R175Q
12	45270407	G	C	NELL2	NM_001145107	NELL2:NM_001145107:exon1:c.C102G:p.H34Q
12	49391634	C	T	DDN	NM_015086	DDN:NM_015086:exon2:c.G1025A:p.G342D
12	49420078	C	T	KMT2D	NM_003482	KMT2D:NM_003482:exon48:c.G15671A:p.R5224H
12	49743098	G	A	DNAJC22	NM_001304944,NM_024902	DNAJC22:NM_024902:exon2:c.G443A:p.R148H,DNAJC22:NM_001304944:exon3:c.G443A:p.R148H
12	51773409	C	T	GALNT6	NM_007210	GALNT6:NM_007210:exon3:c.G157A:p.V53I
12	52642506	C	T	KRT7	NM_005556	KRT7:NM_005556:exon9:c.C1372T:p.R458W
12	52822128	G	A	KRT75	NM_004693	KRT75:NM_004693:exon7:c.C1294T:p.R432C
12	56213186	A	G	ORMDL2	NM_014182	ORMDL2:NM_014182:exon3:c.A235G:p.K79E

12	56351128	G	T	PMEL	NM_001200053,NM_001200054,NM_006928	PMEL:NM_001200053:exon4:c.C701A:p.P234H,PMEL:NM_001200054:exon6:c.C959A:p.P320H,PMEL:NM_006928:exon7:c.C959A:p.P320H
12	56420706	G	C	IKZF4	NM_022465	IKZF4:NM_022465:exon4:c.G428C:p.G143A
12	56536651	A	G	ESYT1	NM_001184796,NM_015292	ESYT1:NM_001184796:exon27:c.A2951G:p.Q984R,ESYT1:NM_015292:exon27:c.A2921G:p.Q974R
12	57351020	C	A	RDH16	NM_003708	RDH16:NM_003708:exon1:c.G227T:p.R76M
12	57493541	T	G	STAT6	NM_001178078,NM_001178079,NM_001178080,NM_001178081,NM_003153	.
12	57919529	C	G	MBD6	NM_052897	MBD6:NM_052897:exon6:c.C778G:p.L260V
12	58204606	C	T	AVIL	NM_006576	AVIL:NM_006576:exon5:c.G551A:p.R184H
12	62861043	G	A	MON2	NM_001278469,NM_001278470,NM_001278471,NM_015026	MON2:NM_001278469:exon1:c.G56A:p.S19N,MON2:NM_001278470:exon1:c.G56A:p.S19N,MON2:NM_001278471:exon1:c.G56A:p.S19N,MON2:NM_015026:exon1:c.G56A:p.S19N
12	65564671	C	G	LEMD3	NM_001167614,NM_014319	LEMD3:NM_001167614:exon1:c.C1295G:p.S432C,LEMD3:NM_014319:exon1:c.C1295G:p.S432C
12	78225384	A	C	NAV3	NM_001024383,NM_014903	NAV3:NM_001024383:exon1:c.A143C:p.E48A,NAV3:NM_014903:exon1:c.A143C:p.E48A
12	81064181	T	C	PTPRQ	NM_001145026	PTPRQ:NM_001145026:exon39:c.T5996C:p.V1999A
12	102576340	G	A	PARPBP	NM_017915	PARPBP:NM_017915:exon9:c.G1198A:p.V400M
12	104391279	A	G	GLT8D2	NM_031302	GLT8D2:NM_031302:exon7:c.T437C:p.I146T
12	120593094	G	A	GCN1L1	NM_006836	GCN1L1:NM_006836:exon30:c.C3581T:p.A1194V
12	122995650	A	C	RSRC2	NM_023012	.
12	123879668	G	C	SETD8	NM_020382	SETD8:NM_020382:exon4:c.G364C:p.G122R
12	125302144	A	C	SCARB1	NM_001082959,NM_005505	SCARB1:NM_001082959:exon2:c.T236G:p.L79R,SCARB1:NM_005505:exon2:c.T236G:p.L79R
12	129190186	C	A	TMEM132C	NM_001136103	TMEM132C:NM_001136103:exon9:c.C2673A:p.D891E
13	23910339	G	A	SACS	NM_001278055,NM_014363	SACS:NM_001278055:exon8:c.C7235T:p.T2412I,SACS:NM_014363:exon10:c.C7676T:p.T2559I

13	25478083	T	C	CENPJ	NM_018451	CENPJ:NM_018451:exon8:c.A2806G:p.S936G
13	25671292	C	T	PABPC3	NM_030979	PABPC3:NM_030979:exon1:c.C956T:p.T319I
13	25831336	T	C	MTMR6	NM_004685	MTMR6:NM_004685:exon9:c.A1093G:p.M365V
13	32821610	A	G	FRY	NM_023037	FRY:NM_023037:exon48:c.A6979G:p.T2327A
13	32972548	C	T	BRCA2	NM_000059	BRCA2:NM_000059:exon27:c.C9898T:p.P3300S
13	33016603	G	A	N4BP2L2	NM_001278432,NM_033111	N4BP2L2:NM_001278432:exon7:c.C2026T:p.R676C,N4BP2L2:NM_033111:exon7:c.C2071T:p.R691C
13	48611934	G	A	NUDT15	NM_001304745,NM_018283	NUDT15:NM_001304745:exon1:c.G52A:p.V18I,NUDT15:NM_018283:exon1:c.G52A:p.V18I
13	53624380	A	C	OLFM4	NM_006418	OLFM4:NM_006418:exon5:c.A1007C:p.N336T
13	67802256	T	C	PCDH9	NM_020403,NM_203487	PCDH9:NM_020403:exon2:c.A317G:p.E106G,PCDH9:NM_203487:exon2:c.A317G:p.E106G
13	77759465	T	C	MYCBP2	NM_015057	MYCBP2:NM_015057:exon32:c.A4492G:p.I1498V
13	102235561	C	T	ITGBL1	NM_001271754,NM_001271755,NM_001271756,NM_004791	.
13	103389158	A	T	CCDC168	NM_001146197	CCDC168:NM_001146197:exon4:c.T13889A:p.M4630K
13	103473476	C	T	BIVM-ERCC5,BIVM	NM_001159596,NM_001204425,NM_017693	BIVM:NM_001159596:exon3:c.C8T:p.A3V,BIVM-ERCC5:NM_001204425:exon3:c.C695T:p.A232V,BIVM:NM_017693:exon5:c.C695T:p.A232V
13	115007743	A	G	CDC16	NM_001078645,NM_003903	CDC16:NM_001078645:exon6:c.A529G:p.T177A,CDC16:NM_003903:exon6:c.A529G:p.T177A
14	20249176	G	A	OR4M1	NM_001005500	OR4M1:NM_001005500:exon1:c.G695A:p.G232D
14	21052230	G	C	RNASE11	NM_145250	RNASE11:NM_145250:exon3:c.C404G:p.P135R
14	21550028	C	T	ARHGEF40	NM_001278529,NM_001278530,NM_018071	ARHGEF40:NM_001278529:exon14:c.C859T:p.R287W,ARHGEF40:NM_001278530:exon14:c.C859T:p.R287W,ARHGEF40:NM_018071:exon14:c.C3001T:p.R1001W
14	23371408	T	C	RBM23	NM_001077351,NM_001077352,NM_018107	RBM23:NM_001077352:exon9:c.A1012G:p.K338E,RBM23:NM_018107:exon10:c.A1066G:p.K356E,RBM23:NM_001077351:exon11:c.A1114G:p.K372E

14	23396746	G	A	PRMT5	NM_001039619,NM_001282953,NM_001282954,NM_001282955,NM_006109	PRMT5:NM_001282953:exon3:c.C256T:p.H86Y,PRMT5:NM_001282954:exon3:c.C121T:p.H41Y,PRMT5:NM_001282955:exon3:c.C307T:p.H103Y,PRMT5:NM_001039619:exon4:c.C388T:p.H130Y,PRMT5:NM_006109:exon4:c.C439T:p.H147Y
14	24769991	A	T	NOP9	NM_001286367,NM_174913	NOP9:NM_001286367:exon2:c.A625T:p.T209S,NOP9:NM_174913:exon2:c.A625T:p.T209S
14	39901200	C	T	FBXO33	NM_203301	FBXO33:NM_203301:exon1:c.G167A:p.R56Q
14	53525286	A	T	DDHD1	NM_001160147,NM_001160148,NM_030637	DDHD1:NM_001160148:exon9:c.T1901A:p.I634N,DDHD1:NM_030637:exon9:c.T1901A:p.I634N,DDHD1:NM_001160147:exon10:c.T1922A:p.I641N
14	61180693	T	A	SIX4	NM_017420	SIX4:NM_017420:exon3:c.A1778T:p.N593I
14	65260321	C	T	SPTB	NM_000347,NM_001024858	SPTB:NM_000347:exon13:c.G2060A:p.R687H,SPTB:NM_001024858:exon13:c.G2060A:p.R687H
14	68192861	T	A	RDH12	NM_152443	RDH12:NM_152443:exon6:c.T437A:p.V146D
14	69345796	C	A	ACTN1	NM_001102,NM_001130004	.
14	70925161	A	G	ADAM21	NM_003813	ADAM21:NM_003813:exon2:c.A945G:p.I315M
14	73721320	C	T	PAPLN	NM_173462	PAPLN:NM_173462:exon12:c.C1321T:p.R441W
14	74404653	G	C	FAM161B	NM_152445	FAM161B:NM_152445:exon6:c.C1752G:p.N584K
14	75538484	T	A	ZC2HC1C	NM_024643	ZC2HC1C:NM_024643:exon2:c.T1208A:p.L403Q
14	76525675	G	A	IFT43	NM_001102564	IFT43:NM_001102564:exon5:c.G254A:p.R85H
14	88406259	A	G	GALC	NM_000153,NM_001201401,NM_001201402	GALC:NM_001201401:exon15:c.T1832C:p.L611S,GALC:NM_000153:exon16:c.T1901C:p.L634S,GALC:NM_001201402:exon16:c.T1823C:p.L608S
14	90778748	C	T	NRDE2	NM_017970	NRDE2:NM_017970:exon4:c.G547A:p.D183N
14	93151482	G	A	RIN3	NM_024832	RIN3:NM_024832:exon9:c.G2618A:p.R873H
14	94594946	G	A	IFI27L2	NM_032036	IFI27L2:NM_032036:exon3:c.C104T:p.A35V
14	94848927	A	G	SERPINA1	NM_000295,NM_001002235,NM_001002236,NM_001127700,NM_001127701,NM_001127702,NM_001127703,NM_001127704,NM_001127705,NM_001127706,NM_001127707	.

14	100331926	G	A	EML1	NM_001008707,NM_004434	EML1:NM_001008707:exon3:c.G326A:p.G109D,EML1:NM_004434:exon3:c.G326A:p.G109D
14	104208286	G	T	PPP1R13B	NM_015316	PPP1R13B:NM_015316:exon11:c.C1663A:p.P555T
14	104641674	G	T	KIF26A	NM_015656	KIF26A:NM_015656:exon12:c.G2549T:p.C850F
14	105180903	C	T	INF2	NM_001031714,NM_022489	INF2:NM_001031714:exon21:c.C3404T:p.T1135M,INF2:NM_022489:exon21:c.C3404T:p.T1135M
14	105350602	C	T	CEP170B	NM_001112726,NM_015005	CEP170B:NM_015005:exon8:c.C1276T:p.R426C,CEP170B:NM_001112726:exon9:c.C1486T:p.R496C
14	105359870	G	A	CEP170B	NM_001112726,NM_015005	CEP170B:NM_015005:exon14:c.G3944A:p.R1315H,CEP170B:NM_001112726:exon15:c.G4049A:p.R1350H
15	22368862	G	A	OR4M2	NM_001004719	OR4M2:NM_001004719:exon1:c.G287A:p.G96E
15	22369426	G	A	OR4M2	NM_001004719	OR4M2:NM_001004719:exon1:c.G851A:p.R284H
15	39874573	C	T	THBS1	NM_003246	THBS1:NM_003246:exon3:c.C247T:p.R83W
15	40582849	C	G	PLCB2	NM_001284297,NM_001284298,NM_004573	PLCB2:NM_001284298:exon28:c.G3093C:p.K1031N,PLCB2:NM_001284297:exon29:c.G3126C:p.K1042N,PLCB2:NM_004573:exon29:c.G3138C:p.K1046N
15	40685797	A	C	KNSTRN	NM_033286	KNSTRN:NM_033286:exon9:c.A950C:p.X317S
15	41797046	C	T	LTK	NM_001135685,NM_002344,NM_206961	LTK:NM_001135685:exon15:c.G1655A:p.R552Q,LTK:NM_206961:exon16:c.G1862A:p.R621Q,LTK:NM_002344:exon17:c.G2045A:p.R682Q
15	43545052	G	A	TGM5	NM_004245,NM_201631	TGM5:NM_004245:exon5:c.C521T:p.T174M,TGM5:NM_201631:exon6:c.C767T:p.T256M
15	43552621	T	A	TGM5	NM_004245,NM_201631	TGM5:NM_004245:exon2:c.A167T:p.N56I,TGM5:NM_201631:exon2:c.A167T:p.N56I
15	43585143	G	A	TGM7	NM_052955	TGM7:NM_052955:exon3:c.C203T:p.P68L
15	44107202	C	A	MFAP1	NM_005926	MFAP1:NM_005926:exon3:c.G370T:p.A124S
15	48434456	T	G	SLC24A5	NM_205850	SLC24A5:NM_205850:exon9:c.T1411G:p.C471G
15	48512873	G	A	SLC12A1	NM_000338,NM_001184832	SLC12A1:NM_000338:exon3:c.G463A:p.G155S,SLC12A1:NM_001184832:exon3:c.G463A:p.G155S
15	51535155	A	C	CYP19A1	NM_000103,NM_031226	.
15	63594621	T	A	APH1B	NM_001145646,NM_031301	APH1B:NM_001145646:exon4:c.T433A:p.W145R,APH1B:NM_031301:exon

						5:c.T556A:p.W186R
15	65425456	G	A	PDCD7	NM_005707	PDCD7:NM_005707:exon1:c.C664T:p.R222W
15	65490592	C	T	CILP	NM_003613	CILP:NM_003613:exon9:c.G2032A:p.V678M
15	66386799	C	T	MEGF11	NM_032445	MEGF11:NM_032445:exon5:c.G335A:p.C112Y
15	70959808	A	G	UACA	NM_001008224,NM_018003	UACA:NM_001008224:exon16:c.T3176C:p.L1059S,UACA:NM_018003:exon16:c.T3215C:p.L1072S
15	72283488	G	A	MYO9A	NM_006901	MYO9A:NM_006901:exon11:c.C1678T:p.R560C
15	75646094	G	A	NEIL1	NM_001256552,NM_024608	NEIL1:NM_001256552:exon6:c.G991A:p.G331R,NEIL1:NM_024608:exon6:c.G733A:p.G245R
15	80450501	G	T	FAH	NM_000137	FAH:NM_000137:exon2:c.G181T:p.V61F
15	85401106	G	A	ALPK3	NM_020778	ALPK3:NM_020778:exon6:c.G3743A:p.R1248H
15	89760506	C	T	RLBP1	NM_000326	RLBP1:NM_000326:exon5:c.G191A:p.R64Q
15	90630816	G	A	IDH2	NM_001289910,NM_001290114,NM_002168	.
15	91016096	G	A	IQGAP1	NM_003870	IQGAP1:NM_003870:exon19:c.G2203A:p.A735T
15	94942206	C	T	MCTP2	NM_001159643,NM_001159644,NM_018349	MCTP2:NM_001159644:exon7:c.C569T:p.P190L,MCTP2:NM_001159643:exon14:c.C1805T:p.P602L,MCTP2:NM_018349:exon14:c.C1805T:p.P602L
15	98513207	C	G	ARRDC4	NM_183376	ARRDC4:NM_183376:exon6:c.C977G:p.S326C
15	99671011	G	A	SYNM	NM_015286,NM_145728	UNKNOWN
16	780592	T	G	NARFL	NM_001304799,NM_022493	NARFL:NM_022493:exon11:c.A1256C:p.H419P,NARFL:NM_001304799:exon12:c.A950C:p.H317P
16	837116	C	G	RPUSD1	NM_058192	RPUSD1:NM_058192:exon4:c.G370C:p.E124Q
16	2354110	C	T	ABCA3	NM_001089	ABCA3:NM_001089:exon12:c.G1327A:p.V443M
16	7568208	G	T	RBFOX1	NM_001142333,NM_001142334,NM_018723,NM_145891,NM_145892,NM_145893	RBFOX1:NM_145891:exon2:c.G147T:p.Q49H,RBFOX1:NM_145892:exon2:c.G147T:p.Q49H,RBFOX1:NM_145893:exon2:c.G147T:p.Q49H,RBFOX1:NM_001142334:exon3:c.G87T:p.Q29H,RBFOX1:NM_001142333:exon5:c.G87T:p.Q29H,RBFOX1:NM_018723:exon5:c.G87T:p.Q29H
16	8895699	A	T	PMM2	NM_000303	PMM2:NM_000303:exon2:c.A110T:p.Q37L

16	9858774	A	G	GRIN2A	NM_000833,NM_001134407,NM_001134408	GRIN2A:NM_001134407:exon13:c.T2627C:p.I876T,GRIN2A:NM_001134408:exon13:c.T2627C:p.I876T,GRIN2A:NM_000833:exon14:c.T2627C:p.I876T
16	10273869	T	C	GRIN2A	NM_000833,NM_001134407,NM_001134408	GRIN2A:NM_001134407:exon2:c.A400G:p.I134V,GRIN2A:NM_001134408:exon2:c.A400G:p.I134V,GRIN2A:NM_000833:exon3:c.A400G:p.I134V
16	19884011	C	T	GPRC5B	NM_001304771,NM_016235	GPRC5B:NM_001304771:exon2:c.G550A:p.A184T,GPRC5B:NM_016235:exon2:c.G157A:p.A53T
16	24802206	A	T	TNRC6A	NM_014494	TNRC6A:NM_014494:exon6:c.A2243T:p.N748I
16	29706207	C	T	QPRT	NM_014298	QPRT:NM_014298:exon2:c.C236T:p.S79L
16	31448170	T	C	ZNF843	NM_001136509	ZNF843:NM_001136509:exon2:c.A1G:p.M1V
16	31471240	C	A	ARMC5	NM_001105247,NM_001288767,NM_001301820,NM_024742	ARMC5:NM_001105247:exon1:c.C395A:p.A132E,ARMC5:NM_024742:exon1:c.C395A:p.A132E,ARMC5:NM_001301820:exon2:c.C491A:p.A164E,ARMC5:NM_001288767:exon3:c.C680A:p.A227E
16	50187728	A	C	PAPD5	NM_001040284,NM_001040285	PAPD5:NM_001040284:exon2:c.A151C:p.T51P,PAPD5:NM_001040285:exon2:c.A151C:p.T51P
16	51171333	G	A	SALL1	NM_001127892,NM_002968	SALL1:NM_001127892:exon3:c.C3374T:p.A1125V,SALL1:NM_002968:exon3:c.C3665T:p.A1222V
16	55361526	G	A	IRX6	NM_024335	IRX6:NM_024335:exon4:c.G442A:p.G148S
16	56904040	G	A	SLC12A3	NM_000339,NM_001126107,NM_001126108	SLC12A3:NM_000339:exon5:c.G634A:p.G212S,SLC12A3:NM_001126107:exon5:c.G631A:p.G211S,SLC12A3:NM_001126108:exon5:c.G634A:p.G212S
16	58541762	C	T	NDRG4	NM_001130487,NM_001242833,NM_001242834,NM_001242835,NM_001242836,NM_020465,NM_022910	NDRG4:NM_001242834:exon9:c.C725T:p.T242M,NDRG4:NM_001242835:exon9:c.C671T:p.T224M,NDRG4:NM_001242836:exon9:c.C671T:p.T224M,NDRG4:NM_001242833:exon10:c.C761T:p.T254M,NDRG4:NM_001130487:exon11:c.C827T:p.T276M,NDRG4:NM_020465:exon11:c.C767T:p.T256M,NDRG4:NM_022910:exon11:c.C767T:p.T256M
16	66430060	C	T	CDH5	NM_001795	CDH5:NM_001795:exon8:c.C1316T:p.P439L
16	70164334	A	G	PDPR	NM_017990	PDPR:NM_017990:exon7:c.A616G:p.I206V
16	70432148	C	T	ST3GAL2	NM_006927	ST3GAL2:NM_006927:exon2:c.G286A:p.V96I
16	70884524	C	G	HYDIN	NM_001270974	HYDIN:NM_001270974:exon74:c.G12478C:p.E4160Q
16	70894087	T	C	HYDIN	NM_001270974	HYDIN:NM_001270974:exon71:c.A12013G:p.T4005A
16	70902559	C	T	HYDIN	NM_001270974	HYDIN:NM_001270974:exon66:c.G11224A:p.V3742I

16	70902568	C	T	HYDIN	NM_001270974	HYDIN:NM_001270974:exon66:c.G11215A:p.A3739T
16	70954606	C	T	HYDIN	NM_001270974	HYDIN:NM_001270974:exon46:c.G7673A:p.G2558E
16	70972620	G	C	HYDIN	NM_001270974	HYDIN:NM_001270974:exon44:c.C6892G:p.R2298G
16	70989335	G	A	HYDIN	NM_001270974	HYDIN:NM_001270974:exon40:c.C6259T:p.R2087C
16	71007809	C	T	HYDIN	NM_001270974	HYDIN:NM_001270974:exon34:c.G5152A:p.V1718M
16	71098649	T	C	HYDIN	NM_001198542,NM_001198543,NM_001270974,NM_017558	HYDIN:NM_001198542:exon16:c.A2251G:p.N751D,HYDIN:NM_001198543:exon16:c.A2221G:p.N741D,HYDIN:NM_001270974:exon16:c.A2170G:p.N724D,HYDIN:NM_017558:exon16:c.A2170G:p.N724D
16	71101200	T	C	HYDIN	NM_001198542,NM_001198543,NM_001270974,NM_017558	HYDIN:NM_001198542:exon15:c.A2149G:p.T717A,HYDIN:NM_001198543:exon15:c.A2119G:p.T707A,HYDIN:NM_001270974:exon15:c.A2068G:p.T690A,HYDIN:NM_017558:exon15:c.A2068G:p.T690A
16	71122408	C	T	HYDIN	NM_001198542,NM_001198543,NM_001270974,NM_017558	HYDIN:NM_001198542:exon12:c.G1547A:p.G516D,HYDIN:NM_001198543:exon12:c.G1517A:p.G506D,HYDIN:NM_001270974:exon12:c.G1466A:p.G489D,HYDIN:NM_017558:exon12:c.G1466A:p.G489D
16	71127814	C	G	HYDIN	NM_001198542,NM_001198543,NM_001270974,NM_017558	HYDIN:NM_001198542:exon11:c.G1433C:p.R478P,HYDIN:NM_001198543:exon11:c.G1403C:p.R468P,HYDIN:NM_001270974:exon11:c.G1352C:p.R451P,HYDIN:NM_017558:exon11:c.G1352C:p.R451P
16	72184631	A	C	PMFBP1	NM_001160213,NM_031293	PMFBP1:NM_031293:exon5:c.T512G:p.I171S,PMFBP1:NM_001160213:exon6:c.T77G:p.I26S
16	72984669	G	A	ZFHX3	NM_001164766,NM_006885	ZFHX3:NM_001164766:exon2:c.C173T:p.S58L,ZFHX3:NM_006885:exon3:c.C2915T:p.S972L
16	77822740	A	G	VAT1L	NM_020927	VAT1L:NM_020927:exon1:c.A161G:p.N54S
16	81030993	G	A	CMC2	NM_020188	CMC2:NM_020188:exon2:c.C7T:p.P3S
16	81324057	G	A	BCO1	NM_017429	BCO1:NM_017429:exon11:c.G1519A:p.V507I
16	84444206	C	G	ATP2C2	NM_001286527,NM_001291454,NM_014861	ATP2C2:NM_001291454:exon3:c.C59G:p.S20C,ATP2C2:NM_001286527:exon5:c.C450G:p.I150M,ATP2C2:NM_014861:exon5:c.C450G:p.I150M
16	89347285	T	C	ANKRD11	NM_001256182,NM_001256183,NM_013275	ANKRD11:NM_001256183:exon9:c.A5665G:p.K1889E,ANKRD11:NM_013275:exon9:c.A5665G:p.K1889E,ANKRD11:NM_001256182:exon10:c.A5665G:p.K1889E
16	89986025	T	C	MC1R	NM_002386	MC1R:NM_002386:exon1:c.T359C:p.I120T

17	3533618	C	G	SHPK	NM_013276	SHPK:NM_013276:exon2:c.G191C:p.R64T
17	4389545	G	A	SPNS3	NM_182538	SPNS3:NM_182538:exon10:c.G1202A:p.R401Q
17	4586159	C	T	PELP1	NM_001278241,NM_014389	PELP1:NM_014389:exon4:c.G659A:p.R220Q,PELP1:NM_001278241:exon5:c.G68A:p.R23Q
17	6704182	C	A	TEKT1	NM_053285	TEKT1:NM_053285:exon7:c.G933T:p.K311N
17	7847814	G	A	CNTROB	NM_001037144,NM_053051	CNTROB:NM_001037144:exon12:c.G1592A:p.R531Q,CNTROB:NM_053051:exon12:c.G1592A:p.R531Q
17	7948638	C	T	ALOX15B	NM_001039130,NM_001039131,NM_001141	ALOX15B:NM_001039130:exon7:c.C932T:p.A311V,ALOX15B:NM_001039131:exon7:c.C932T:p.A311V,ALOX15B:NM_001141:exon7:c.C932T:p.A311V
17	7979610	G	A	ALOX12B	NM_001139	ALOX12B:NM_001139:exon11:c.C1415T:p.S472L
17	8172434	G	A	PFAS	NM_012393	PFAS:NM_012393:exon28:c.G3869A:p.R1290H
17	8647006	C	T	CCDC42	NM_001158261,NM_144681	CCDC42:NM_001158261:exon3:c.G232A:p.E78K,CCDC42:NM_144681:exon3:c.G232A:p.E78K
17	10405194	C	T	MYH1	NM_005963	MYH1:NM_005963:exon25:c.G3146A:p.R1049Q
17	15971325	C	T	NCOR1	NM_001190440,NM_006311	NCOR1:NM_001190440:exon31:c.G4672A:p.V1558M,NCOR1:NM_006311:exon32:c.G4624A:p.V1542M
17	21318698	C	T	KCNJ12,KCNJ18	NM_001194958,NM_021012	KCNJ18:NM_001194958:exon3:c.C44T:p.S15L,KCNJ12:NM_021012:exon3:c.C44T:p.S15L
17	21319121	C	T	KCNJ12,KCNJ18	NM_001194958,NM_021012	KCNJ18:NM_001194958:exon3:c.C467T:p.P156L,KCNJ12:NM_021012:exon3:c.C467T:p.P156L
17	21319230	G	C	KCNJ12,KCNJ18	NM_001194958,NM_021012	KCNJ18:NM_001194958:exon3:c.G576C:p.Q192H,KCNJ12:NM_021012:exon3:c.G576C:p.Q192H
17	21319943	A	G	KCNJ12,KCNJ18	NM_001194958,NM_021012	KCNJ18:NM_001194958:exon3:c.A1289G:p.E430G,KCNJ12:NM_021012:exon3:c.A1289G:p.E430G
17	26699260	C	G	SARM1	NM_015077	SARM1:NM_015077:exon2:c.C206G:p.A69G
17	26861773	C	T	FOXN1	NM_003593	FOXN1:NM_003593:exon7:c.C1184T:p.P395L
17	26861884	C	A	FOXN1	NM_003593	FOXN1:NM_003593:exon7:c.C1295A:p.P432H
17	33319683	C	T	LIG3	NM_002311,NM_013975	LIG3:NM_002311:exon8:c.C1427T:p.S476L,LIG3:NM_013975:exon8:c.C1427T:p.S476L

17	33456472	C	T	FNDC8	NM_017559	FNDC8:NM_017559:exon3:c.C617T:p.P206L
17	33767871	C	T	SLFN13	NM_144682	SLFN13:NM_144682:exon6:c.G2437A:p.V813I
17	35600469	C	G	ACACA	NM_198834,NM_198836,NM_198837,NM_198838,NM_198839	ACACA:NM_198837:exon20:c.G2464C:p.D822H,ACACA:NM_198838:exon21:c.G2404C:p.D802H,ACACA:NM_198834:exon22:c.G2749C:p.D917H,ACACA:NM_198836:exon22:c.G2638C:p.D880H,ACACA:NM_198839:exon26:c.G2638C:p.D880H
17	37101367	T	A	FBXO47	NM_001008777	FBXO47:NM_001008777:exon7:c.A639T:p.L213F
17	37817129	G	A	STARD3	NM_001165937,NM_001165938,NM_006804	STARD3:NM_001165938:exon11:c.G967A:p.V323M,STARD3:NM_001165937:exon12:c.G1021A:p.V341M,STARD3:NM_006804:exon12:c.G1021A:p.V341M
17	39724592	A	G	KRT9	NM_000226	KRT9:NM_000226:exon6:c.T1216C:p.C406R
17	40256845	G	A	DHX58	NM_024119	DHX58:NM_024119:exon11:c.C1502T:p.T501M
17	40714900	T	A	COASY	NM_001042529,NM_001042532,NM_025233	COASY:NM_025233:exon1:c.T260A:p.L87Q,COASY:NM_001042529:exon2:c.T260A:p.L87Q,COASY:NM_001042532:exon3:c.T347A:p.L116Q
17	45266522	T	C	CDC27	NM_001114091,NM_001256,NM_001293089	CDC27:NM_001114091:exon1:c.A17G:p.E6G,CDC27:NM_001256:exon1:c.A17G:p.E6G,CDC27:NM_001293089:exon1:c.A17G:p.E6G
17	45266535	T	C	CDC27	NM_001114091,NM_001256,NM_001293089	CDC27:NM_001114091:exon1:c.A4G:p.T2A,CDC27:NM_001256:exon1:c.A4G:p.T2A,CDC27:NM_001293089:exon1:c.A4G:p.T2A
17	45925777	C	G	SP6	NM_001258248,NM_199262	SP6:NM_001258248:exon2:c.G19C:p.G7R,SP6:NM_199262:exon2:c.G19C:p.G7R
17	46052944	T	C	CDK5RAP3	NM_001278197,NM_001278217,NM_176096	CDK5RAP3:NM_001278217:exon6:c.T317C:p.I106T,CDK5RAP3:NM_001278197:exon7:c.T653C:p.I218T,CDK5RAP3:NM_176096:exon7:c.T578C:p.I193T
17	46627980	C	T	HOXB3	NM_002146	HOXB3:NM_002146:exon4:c.G1012A:p.G338R
17	48184448	G	A	PDK2	NM_001199898,NM_001199899,NM_002611	PDK2:NM_001199899:exon6:c.G424A:p.D142N,PDK2:NM_002611:exon6:c.G616A:p.D206N,PDK2:NM_001199898:exon7:c.G424A:p.D142N
17	56620317	T	C	C17orf47	NM_001038704	C17orf47:NM_001038704:exon1:c.A1231G:p.R411G
17	61780961	A	C	STRADA	NM_001003786,NM_001003787,NM_001003788	STRADA:NM_001003786:exon11:c.T1183G:p.X395G,STRADA:NM_001003788:exon12:c.T1120G:p.X374G,STRADA:NM_001003787:exon13:c.T1294G:p.X432G

17	71357828	C	T	SDK2	NM_001144952	SDK2:NM_001144952:exon39:c.G5462A:p.G1821E
17	73759113	G	A	GALK1	NM_000154	GALK1:NM_000154:exon4:c.C593T:p.A198V
17	74154550	T	C	RNF157	NM_052916	RNF157:NM_052916:exon13:c.A1337G:p.E446G
17	76455168	G	A	DNAH17	NM_173628	DNAH17:NM_173628:exon61:c.C9776T:p.A3259V
17	76795028	C	T	USP36	NM_025090	USP36:NM_025090:exon19:c.G3202A:p.V1068M
17	76887763	G	A	KIAA1731NL	NM_001243540,NM_001243541	KIAA1731NL:NM_001243540:exon3:c.C823T:p.R275W,KIAA1731NL:NM_001243541:exon3:c.C649T:p.R217W
17	77914756	G	A	TBC1D16	NM_001271844,NM_001271845,NM_019020	TBC1D16:NM_001271844:exon8:c.C1081T:p.P361S,TBC1D16:NM_001271845:exon8:c.C1120T:p.P374S,TBC1D16:NM_019020:exon12:c.C2206T:p.P736S
17	78395694	C	T	ENDOV	NM_173627	ENDOV:NM_173627:exon3:c.C295T:p.R99X
17	79826370	C	T	ARHGDIA	NM_001301240,NM_001301241,NM_001301242	ARHGDIA:NM_001301240:exon7:c.G593A:p.R198H,ARHGDIA:NM_001301241:exon7:c.G593A:p.R198H
17	80863817	C	T	TBCD	NM_005993	TBCD:NM_005993:exon20:c.C1810T:p.P604S
18	7013921	C	T	LAMA1	NM_005559	LAMA1:NM_005559:exon23:c.G3256A:p.D1086N
18	7080418	G	A	LAMA1	NM_005559	LAMA1:NM_005559:exon2:c.C100T:p.H34Y
18	14542931	C	T	POTEC	NM_001137671	POTEC:NM_001137671:exon1:c.G215A:p.C72Y
18	21761123	C	T	OSBPL1A	NM_001242508,NM_018030,NM_080597	OSBPL1A:NM_018030:exon5:c.G259A:p.V87M,OSBPL1A:NM_001242508:exon7:c.G652A:p.V218M,OSBPL1A:NM_080597:exon19:c.G1798A:p.V600M
18	39537579	T	G	PIK3C3	NM_002647	PIK3C3:NM_002647:exon2:c.T113G:p.V38G
18	44122704	C	T	LOXHD1	NM_001145472,NM_144612	LOXHD1:NM_001145472:exon6:c.G401A:p.G134D,LOXHD1:NM_144612:exon24:c.G3734A:p.G1245D
19	1046353	C	T	ABCA7	NM_019112	ABCA7:NM_019112:exon13:c.C1570T:p.R524W
19	1495526	G	C	REEP6	NM_138393	REEP6:NM_138393:exon3:c.G268C:p.V90L
19	8176044	C	T	FBN3	NM_032447	FBN3:NM_032447:exon32:c.G4108A:p.V1370M
19	9057936	G	A	MUC16	NM_024690	MUC16:NM_024690:exon3:c.C29510T:p.T9837I
19	9721564	C	T	ZNF561	NM_152289	ZNF561:NM_152289:exon6:c.G773A:p.C258Y
19	10469919	G	A	TYK2	NM_003331	TYK2:NM_003331:exon15:c.C2107T:p.R703W

19	10472988	C	T	TYK2	NM_003331	TYK2:NM_003331:exon11:c.G1621A:p.G541R
19	12186411	A	G	ZNF844	NM_001136501	ZNF844:NM_001136501:exon4:c.A476G:p.Q159R
19	12759074	G	C	MAN2B1	NM_000528,NM_001173498	MAN2B1:NM_000528:exon21:c.C2579G:p.A860G,MAN2B1:NM_001173498:exon21:c.C2576G:p.A859G
19	14512363	C	A	CD97	NM_001025160,NM_001784,NM_078481	ADGRE5:NM_001784:exon8:c.C784A:p.P262T,ADGRE5:NM_001025160:exon9:c.C916A:p.P306T,ADGRE5:NM_078481:exon10:c.C1063A:p.P355T
19	14561754	G	T	PKN1	NM_002741,NM_213560	PKN1:NM_002741:exon6:c.G803T:p.R268L,PKN1:NM_213560:exon6:c.G821T:p.R274L
19	15734882	C	G	CYP4F8	NM_007253	UNKNOWN
19	15806834	T	C	CYP4F12	NM_023944	CYP4F12:NM_023944:exon10:c.T1204C:p.C402R
19	15918009	G	A	OR10H1	NM_013940	OR10H1:NM_013940:exon1:c.C839T:p.T280M
19	16275664	G	A	CIB3	NM_001300922,NM_054113	CIB3:NM_001300922:exon3:c.C260T:p.T87M,CIB3:NM_054113:exon5:c.C407T:p.T136M
19	17306242	G	A	MYO9B	NM_001130065,NM_004145	MYO9B:NM_001130065:exon22:c.G4006A:p.D1336N,MYO9B:NM_004145:exon22:c.G4006A:p.D1336N
19	19643535	G	A	YJEFN3	NM_001190328,NM_198537	YJEFN3:NM_001190328:exon2:c.G154A:p.V52M,YJEFN3:NM_198537:exon3:c.G304A:p.V102M
19	33703847	T	C	SLC7A10	NM_019849	SLC7A10:NM_019849:exon3:c.A418G:p.M140V
19	36211118	G	A	KMT2B	NM_014727	KMT2B:NM_014727:exon3:c.G869A:p.R290H
19	36212009	C	G	KMT2B	NM_014727	KMT2B:NM_014727:exon3:c.C1760G:p.P587R
19	36236773	G	A	PSENEN	NM_001281532	.
19	36357242	G	T	KIRREL2	NM_199180	KIRREL2:NM_199180:exon15:c.G1975T:p.A659S
19	36674347	T	C	ZNF565	NM_001042474,NM_152477	ZNF565:NM_001042474:exon5:c.A521G:p.K174R,ZNF565:NM_152477:exon5:c.A521G:p.K174R
19	37726476	C	T	ZNF383	NM_152604	ZNF383:NM_152604:exon3:c.C35T:p.S12F
19	38377773	G	A	WDR87	NM_001291088,NM_031951	WDR87:NM_001291088:exon6:c.C6538T:p.R2180W,WDR87:NM_031951:exon6:c.C6421T:p.R2141W
19	38877754	A	G	GGN	NM_152657	GGN:NM_152657:exon3:c.T148C:p.W50R
19	39390785	C	T	NFKBIB	NM_002503	NFKBIB:NM_002503:exon1:c.C113T:p.A38V

19	39787237	G	T	IFNL1	NM_172140	.
19	45153329	G	A	PVR	NM_001135768,NM_001135769,NM_001135770,NM_006505	PVR:NM_001135768:exon3:c.G676A:p.E226K,PVR:NM_001135769:exon3:c.G676A:p.E226K,PVR:NM_001135770:exon3:c.G676A:p.E226K,PVR:NM_006505:exon3:c.G676A:p.E226K
19	45411122	G	A	APOE	NM_000041,NM_001302688,NM_001302689,NM_001302690,NM_001302691	APOE:NM_000041:exon3:c.G149A:p.R50H,APOE:NM_001302688:exon3:c.G227A:p.R76H,APOE:NM_001302689:exon3:c.G149A:p.R50H,APOE:NM_001302690:exon3:c.G149A:p.R50H,APOE:NM_001302691:exon3:c.G149A:p.R50H
19	45445586	G	A	APOC4	NM_001646	APOC4:NM_001646:exon1:c.G52A:p.V18M
19	45648738	C	A	PPP1R37	NM_019121	PPP1R37:NM_019121:exon11:c.C1424A:p.P475H
19	47909743	A	C	MEIS3	NM_001009813,NM_001301059,NM_020160	MEIS3:NM_001009813:exon12:c.T1068G:p.H356Q,MEIS3:NM_001301059:exon12:c.T1119G:p.H373Q,MEIS3:NM_020160:exon12:c.T1257G:p.H419Q
19	49965959	G	A	ALDH16A1	NM_001145396,NM_153329	ALDH16A1:NM_001145396:exon7:c.G892A:p.A298T,ALDH16A1:NM_153329:exon8:c.G1045A:p.A349T
19	50796922	G	A	MYH14	NM_001077186,NM_001145809,NM_024729	MYH14:NM_024729:exon37:c.G5324A:p.R1775H,MYH14:NM_001077186:exon38:c.G5348A:p.R1783H,MYH14:NM_001145809:exon39:c.G5447A:p.R1816H
19	52090498	A	C	ZNF175	NM_007147	ZNF175:NM_007147:exon5:c.A914C:p.N305T
19	54744387	C	T	LILRA6,LILRB3	NM_001081450,NM_006864,NM_024318	LILRB3:NM_001081450:exon6:c.G1021A:p.V341M,LILRB3:NM_006864:exon6:c.G1021A:p.V341M,LILRA6:NM_024318:exon6:c.G1012A:p.V338M
19	55718153	C	T	PTPRH	NM_001161440,NM_002842	PTPRH:NM_001161440:exon3:c.G256A:p.V86M,PTPRH:NM_002842:exon3:c.G256A:p.V86M
19	57956321	A	T	ZNF749	NM_001023561	ZNF749:NM_001023561:exon3:c.A1805T:p.H602L
19	58058561	C	G	ZNF550	NM_001277090,NM_001277091,NM_001277092	ZNF550:NM_001277090:exon4:c.G1051C:p.A351P,ZNF550:NM_001277091:exon4:c.G1051C:p.A351P,ZNF550:NM_001277092:exon4:c.G1051C:p.A351P
19	58806424	G	A	ZNF8	NM_021089	ZNF8:NM_021089:exon4:c.G1250A:p.R417Q
19	58967729	G	A	ZNF324B	NM_207395	ZNF324B:NM_207395:exon4:c.G1418A:p.R473H
20	1286411	G	A	SNPH	NM_014723	SNPH:NM_014723:exon6:c.G1198A:p.V400M
20	3102309	C	T	UBOX5	NM_001267584,NM_014948,NM_199415	UBOX5:NM_001267584:exon3:c.G976A:p.A326T,UBOX5:NM_014948:exon3:c.G976A:p.A326T,UBOX5:NM_199415:exon3:c.G976A:p.A326T

20	23401963	G	A	NAPB	NM_001283018,NM_001283020,NM_022080	NAPB:NM_001283018:exon1:c.C77T:p.S26F,NAPB:NM_001283020:exon1:c.C77T:p.S26F,NAPB:NM_022080:exon1:c.C77T:p.S26F
20	23401975	T	C	NAPB	NM_001283018,NM_001283020,NM_022080	NAPB:NM_001283018:exon1:c.A65G:p.K22R,NAPB:NM_001283020:exon1:c.A65G:p.K22R,NAPB:NM_022080:exon1:c.A65G:p.K22R
20	29993891	T	C	DEFB121	NM_001011878	.
20	30512851	C	G	TTLL9	NM_001008409	TTLL9:NM_001008409:exon9:c.C704G:p.S235W
20	31680288	G	A	BPIFB4	NM_182519	BPIFB4:NM_182519:exon9:c.G1168A:p.G390R
20	31814763	G	T	BPIFA3	NM_001042439,NM_178466	BPIFA3:NM_001042439:exon5:c.G541T:p.G181W,BPIFA3:NM_178466:exon6:c.G649T:p.G217W
20	33584309	A	G	MYH7B	NM_020884	MYH7B:NM_020884:exon29:c.A3230G:p.E1077G
20	33591013	G	C	TRPC4AP	NM_015638,NM_199368	TRPC4AP:NM_015638:exon19:c.C2330G:p.S777C,TRPC4AP:NM_199368:exon19:c.C2306G:p.S769C
20	36850938	C	T	KIAA1755	NM_001029864	KIAA1755:NM_001029864:exon10:c.G2330A:p.G777D
20	39991664	C	T	EMILIN3	NM_052846	EMILIN3:NM_052846:exon4:c.G545A:p.R182Q
20	42939673	T	C	FITM2	NM_001080472	FITM2:NM_001080472:exon1:c.A116G:p.K39R
20	44432001	G	A	DNTTIP1	NM_052951	DNTTIP1:NM_052951:exon8:c.G587A:p.R196H
20	44477103	A	C	ACOT8	NM_005469	ACOT8:NM_005469:exon3:c.T474G:p.I158M
20	47990709	A	G	KCNB1	NM_004975	KCNB1:NM_004975:exon2:c.T1388C:p.I463T
20	52779338	C	G	CYP24A1	NM_000782,NM_001128915	CYP24A1:NM_000782:exon7:c.G908C:p.C303S,CYP24A1:NM_001128915:exon7:c.G908C:p.C303S
20	57268940	C	T	NPEPL1	NM_001204872,NM_001204873,NM_024663	NPEPL1:NM_024663:exon2:c.C298T:p.R100W,NPEPL1:NM_001204872:exon3:c.C214T:p.R72W,NPEPL1:NM_001204873:exon3:c.C154T:p.R52W
20	61287999	G	A	SLCO4A1	NM_016354	SLCO4A1:NM_016354:exon2:c.G193A:p.E65K
20	62199985	C	T	HELZ2	NM_001037335	HELZ2:NM_001037335:exon6:c.G1456A:p.D486N
20	62326159	G	A	RTEL1	NM_001283009,NM_001283010,NM_016434,NM_032957	RTEL1:NM_001283010:exon31:c.G2506A:p.A836T,RTEL1:NM_001283009:exon32:c.G3175A:p.A1059T,RTEL1:NM_016434:exon32:c.G3175A:p.A1059T,RTEL1:NM_032957:exon32:c.G3247A:p.A1083T
21	15882747	G	C	SAMSN1	NM_001256370,NM_001286523,NM_022136	SAMSN1:NM_022136:exon5:c.C445G:p.R149G,SAMSN1:NM_001256370:exon6:c.C649G:p.R217G,SAMSN1:NM_001286523:exon6:c.C238G:p.R80G

21	15974340	T	C	LOC388813	NM_001256579	.
21	30419535	A	G	USP16	NM_001001992,NM_001032410,NM_006447	USP16:NM_001001992:exon14:c.A1901G:p.H634R,USP16:NM_006447:exon14:c.A1904G:p.H635R,USP16:NM_001032410:exon15:c.A1904G:p.H635R
21	32582504	C	T	TIAM1	NM_003253	TIAM1:NM_003253:exon12:c.G2245A:p.V749I
21	36164881	C	T	RUNX1	NM_001001890,NM_001754	RUNX1:NM_001001890:exon6:c.G913A:p.D305N,RUNX1:NM_001754:exon9:c.G994A:p.D332N
21	38459567	T	A	TTC3	NM_001001894,NM_003316	TTC3:NM_001001894:exon2:c.T10A:p.F4I,TTC3:NM_003316:exon2:c.T10A:p.F4I
21	45825824	C	G	TRPM2	NM_003307	TRPM2:NM_003307:exon18:c.C2694G:p.I898M
21	47678926	G	C	MCM3AP	NM_003906	MCM3AP:NM_003906:exon16:c.C3661G:p.L1221V
21	47722409	G	C	C21orf58	NM_001286462,NM_001286463,NM_001286476,NM_001286477,NM_058180	C21orf58:NM_001286463:exon6:c.C485G:p.P162R,C21orf58:NM_001286462:exon7:c.C485G:p.P162R,C21orf58:NM_001286476:exon7:c.C485G:p.P162R,C21orf58:NM_001286477:exon7:c.C485G:p.P162R,C21orf58:NM_058180:exon7:c.C803G:p.P268R
21	47738110	C	T	C21orf58	NM_058180	C21orf58:NM_058180:exon2:c.G125A:p.R42H
22	19222234	A	G	CLTCL1	NM_001835,NM_007098	.
22	21989008	C	G	CCDC116	NM_152612	CCDC116:NM_152612:exon4:c.C656G:p.S219C
22	24743090	C	T	SPECC1L	NM_001145468,NM_001254732,NM_015330	SPECC1L:NM_001145468:exon10:c.C2689T:p.P897S,SPECC1L:NM_001254732:exon10:c.C2689T:p.P897S,SPECC1L:NM_015330:exon11:c.C2689T:p.P897S
22	25264363	G	C	SGSM1	NM_001039948,NM_001098497,NM_001098498,NM_133454	SGSM1:NM_001039948:exon11:c.G1015C:p.V339L,SGSM1:NM_001098497:exon11:c.G1015C:p.V339L,SGSM1:NM_001098498:exon11:c.G1015C:p.V339L,SGSM1:NM_133454:exon11:c.G1015C:p.V339L
22	25425282	A	G	KIAA1671	NM_001145206	KIAA1671:NM_001145206:exon1:c.A1316G:p.K439R
22	30200621	A	G	ASCC2	NM_001242906,NM_032204	.
22	31007023	A	T	TCN2	NM_000355,NM_001184726	TCN2:NM_000355:exon2:c.A230T:p.K77M,TCN2:NM_001184726:exon2:c.A230T:p.K77M
22	31521167	C	T	INPP5J	NM_001284285,NM_001284286	INPP5J:NM_001284285:exon2:c.C442T:p.P148S,INPP5J:NM_001284286:exon3:c.C241T:p.P81S
22	37770023	C	T	ELFN2	NM_052906	ELFN2:NM_052906:exon3:c.G1552A:p.E518K

22	37962756	G	A	CDC42EP1	NM_152243	CDC42EP1:NM_152243:exon2:c.G400A:p.V134M
22	38902067	G	A	DDX17	NM_001098504,NM_006386	DDX17:NM_001098504:exon1:c.C176T:p.P59L,DDX17:NM_006386:exon1:c.C176T:p.P59L
22	40814581	C	T	MKL1	NM_001282660,NM_001282661,NM_001282662,NM_020831	MKL1:NM_001282660:exon9:c.G1861A:p.V621M,MKL1:NM_001282661:exon11:c.G1711A:p.V571M,MKL1:NM_001282662:exon12:c.G1861A:p.V621M,MKL1:NM_020831:exon12:c.G1861A:p.V621M
22	42154488	C	G	MEI1	NM_152513	MEI1:NM_152513:exon18:c.C2071G:p.Q691E
22	50656430	G	A	TUBGCP6	NM_020461	TUBGCP6:NM_020461:exon24:c.C5285T:p.P1762L
X	2936657	C	T	ARSH	NM_001011719	ARSH:NM_001011719:exon5:c.C847T:p.R283C
X	10417412	G	A	MID1	NM_000381,NM_001098624,NM_001193277,NM_033289,NM_033290	MID1:NM_000381:exon10:c.C2000T:p.P667L,MID1:NM_001098624:exon10:c.C2000T:p.P667L,MID1:NM_001193277:exon10:c.C2000T:p.P667L,MID1:NM_033289:exon10:c.C1886T:p.P629L,MID1:NM_033290:exon10:c.C2000T:p.P667L
X	27998997	C	T	DCAF8L1	NM_001017930	DCAF8L1:NM_001017930:exon1:c.G455A:p.R152Q
X	40560408	T	C	MED14	NM_004229	.
X	46949289	A	G	RGN	NM_001282848,NM_004683,NM_152869	RGN:NM_004683:exon4:c.A461G:p.N154S,RGN:NM_001282848:exon5:c.A302G:p.N101S,RGN:NM_152869:exon5:c.A461G:p.N154S
X	48369847	G	A	PORCN	NM_001282167,NM_022825,NM_203473,NM_203474,NM_203475	PORCN:NM_203474:exon2:c.G301A:p.V101I,PORCN:NM_001282167:exon3:c.G88A:p.V30I,PORCN:NM_022825:exon3:c.G301A:p.V101I,PORCN:NM_203473:exon3:c.G301A:p.V101I,PORCN:NM_203475:exon3:c.G301A:p.V101I
X	73641555	G	T	SLC16A2	NM_006517	SLC16A2:NM_006517:exon1:c.G83T:p.S28I
X	99849306	A	C	TNMD	NM_022144	TNMD:NM_022144:exon4:c.A370C:p.T124P
X	100503282	A	G	DRP2	NM_001171184,NM_001939	.
X	103294742	G	T	H2BFM	NM_001164416	H2BFM:NM_001164416:exon1:c.G199T:p.V67L
X	114425196	G	A	RBMXL3	NM_001145346	RBMXL3:NM_001145346:exon1:c.G1192A:p.D398N
X	118143214	T	C	LONRF3	NM_001031855,NM_001289109,NM_024778	.
X	140993695	G	A	MAGEC1	NM_005462	MAGEC1:NM_005462:exon4:c.G505A:p.A169T

X	150869021	G	A	PRRG3	NM_024082	PRRG3:NM_024082:exon4:c.G212A:p.R71Q

Supplementary Table S4: List of all Indel variants

CHROM	POS	REF	ALT	GeneName	Gene	AAChange
1	9780693	G	GC	PIK3CD	NM_005026	PIK3CD:NM_005026:exon12:c.1495_1496insC:p.E499fs
1	9781176	T	TC	PIK3CD	NM_005026	.
1	11140610	GA	G	EXOSC10	NM_001001998,NM_002685	.
1	11140610	GAAAA	G	EXOSC10	NM_001001998,NM_002685	.
1	11150700	GC	G	EXOSC10	NM_001001998,NM_002685	EXOSC10:NM_001001998:exon6:c.668delG:p.R223fs,EXOSC10:NM_002685:exon6:c.668delG:p.R223fs
1	11561092	GAGGAGGAGC	G	PTCHD2	NM_020780	PTCHD2:NM_020780:exon2:c.44_52del:p.15_18del
1	14106394	A	ACTC	PRDM2	NM_001007257,NM_012231,NM_015866	PRDM2:NM_001007257:exon3:c.1501_1502insCTC:p.T501delinsTP,PRDM2:NM_012231:exon8:c.2104_2105insCTC:p.T702delinsTP,PRDM2:NM_015866:exon8:c.2104_2105insCTC:p.T702delinsTP
1	19634721	A	ATT	AKR7A2	NM_003689	AKR7A2:NM_003689:exon3:c.521_522insAA:p.A174fs
1	24406692	A	AGC	MYOM3	NM_152372	.
1	26608877	GCCGGGA	G	UBXN11	NM_001077262,NM_145345,NM_183008	UBXN11:NM_001077262:exon11:c.1110_1115del:p.370_372del,UBXN11:NM_145345:exon15:c.1371_1376del:p.457_459del,UBXN11:NM_183008:exon16:c.1470_1475del:p.490_492del
1	46655241	T	TGC	POMGNT1	NM_001243766,NM_001290129,NM_001290130,NM_017739	.
1	50884773	CCGGCGG	C	DMRTA2	NM_032110	DMRTA2:NM_032110:exon3:c.1187_1192del:p.396_398del
1	57538096	G	GA	DAB1	NM_021080	.
1	62910509	AAAG	A	USP1	NM_001017415,NM_001017416,NM_003368	USP1:NM_001017415:exon6:c.659_661del:p.220_221del,USP1:NM_001017416:exon6:c.659_661del:p.220_221del,USP1:NM_003368:exon6:c.659_661del:p.220_221del

1	92944314	C	CAGAG	GFI1	NM_001127215,NM_001127216,NM_005263	.
1	103471456	CCAT	C	COL11A1	NM_001190709,NM_001854,NM_080629,NM_080630	.
1	152681689	G	GGGCTGCTGTAGCTCTGGC	LCE4A	NM_178356	LCE4A:NM_178356:exon1:c.138_139insGGCTGCTGTAGCTCTGGC:p.G46delinsGGCCSSG
1	153509779	TTG	T	S100A5	NM_002962	S100A5:NM_002962:exon4:c.270_271del:p.D90fs
1	153907278	CCTGCTGCTG	C	DENND4B	NM_014856	DENND4B:NM_014856:exon18:c.2722_2730del:p.908_910del
1	154842199	G	GGCTGCTGCTGCT	KCNN3	NM_001204087,NM_002249	KCNN3:NM_001204087:exon1:c.241_242insAGCAGCAGCAGC:p.P81delinsQQQQP,KCNN3:NM_002249:exon1:c.241_242insAGCAGCAGCAGC:p.P81delinsQQQQP
1	154842199	G	GGCTGCTGCTGCTGCT	KCNN3	NM_001204087,NM_002249	KCNN3:NM_001204087:exon1:c.241_242insAGCAGCAGCAGCAGC:p.P81delinsQQQQQP,KCNN3:NM_002249:exon1:c.241_242insAGCAGCAGCAGCAGC:p.P81delinsQQQQQP
1	155026379	T	TG	ADAM15	NM_001261464,NM_001261465,NM_001261466,NM_003815,NM_207191,NM_207194,NM_207195,NM_207196,NM_207197	.
1	160394903	T	TGC	VANGL2	NM_020335	.
1	161192845	CCACACACACACA	C	APOA2	NM_001643	.
1	161192845	CCACACA	C	APOA2	NM_001643	.
1	161192845	CCACACACACACACA	C	APOA2	NM_001643	.
1	161192845	CCACACACA	C	APOA2	NM_001643	.
1	168262522	A	AGT	TBX19	NM_005149	.
1	175129924	C	CCTT	KIAA0040	NM_001162893,NM_001162894,NM_001162895,NM_014656	KIAA0040:NM_001162895:exon3:c.225_226insAAG:p.D76delinsKD,KIAA0040:NM_001162894:exon4:c.225_226insAAG:p.D76delinsKD,KIAA0040

						:NM_014656:exon4:c.225_226insAAG:p.D76delinsKD,KIAA0040:NM_001162893:exon5:c.225_226insAAG:p.D76delinsKD
1	175299236	C	CAA	TNR	NM_003285	TNR:NM_003285:exon21:c.3766_3767insTT:p.R1256fs
1	186276267	GGAGCCTGCACCCACCACTCCCAAA	G	PRG4	NM_001127708,NM_001127709,NM_001127710,NM_001303232,NM_005807	PRG4:NM_001127710:exon4:c.1015_1038del:p.339_346del,PRG4:NM_001127709:exon5:c.1138_1161del:p.380_387del,PRG4:NM_001127708:exon6:c.1294_1317del:p.432_439del,PRG4:NM_001303232:exon6:c.1288_1311del:p.430_437del,PRG4:NM_005807:exon7:c.1417_1440del:p.473_480del
1	209605636	TAGCAGCAGCAGC	T	MIR205HG	NM_001104548	MIR205HG:NM_001104548:exon4:c.252_263del:p.84_88del
1	209800739	G	GC	LAMB3	NM_000228,NM_001017402,NM_001127641	LAMB3:NM_001017402:exon11:c.1473_1474insG:p.Q492fs,LAMB3:NM_000228:exon12:c.1473_1474insG:p.Q492fs,LAMB3:NM_001127641:exon12:c.1473_1474insG:p.Q492fs
1	213068548	G	GATTA	FLVCR1	NM_014053	.
1	225707213	TCGCTCCAGCCTTTCC	T	ENAH	NM_001008493,NM_018212	ENAH:NM_001008493:exon5:c.474_488del:p.158_163del,ENAH:NM_018212:exon5:c.474_488del:p.158_163del
2	10808776	C	CAA	NOL10	NM_001261392,NM_001261394,NM_024894	.
2	27728668	T	TA	GCKR	NM_001486	GCKR:NM_001486:exon10:c.835dupA:p.H278fs
2	48033791	GTAAC	G	MSH6	NM_000179,NM_001281492,NM_001281493,NM_001281494	.
2	61390244	GAGACAGAGTAAGGAAGTGTTGGCCACACCCTT	G	C2orf74	NM_001143959,NM_001143960	C2orf74:NM_001143959:exon3:c.289_320del:p.R97fs,C2orf74:NM_001143960:exon3:c.52_83del:p.R18fs
2	69553373	AAG	A	GFPT1	NM_001244710,NM_002056	.
2	102483033	G	GGC	MAP4K4	NM_001242559,NM_001242560,NM_004834,NM_145686,NM_145687	MAP4K4:NM_004834:exon17:c.1859_1860insGC:p.R620fs,MAP4K4:NM_001242559:exon18:c.2114

						_2115insGC:p.R705fs,MAP4K4:NM_001242560:exon18:c.2093_2094insGC:p.R698fs,MAP4K4:NM_145686:exon18:c.2024_2025insGC:p.R675fs,MAP4K4:NM_145687:exon18:c.2024_2025insGC:p.R675fs
2	103130575	T	TA	SLC9A4	NM_001011552	SLC9A4:NM_001011552:exon8:c.1587dupA:p.L529fs
2	128526579	T	TTAAA	WDR33	NM_001006622,NM_001006623,NM_018383	.
2	131486737	C	CG	GPR148	NM_207364	GPR148:NM_207364:exon1:c.13_14insG:p.L5fs
2	151328284	GA	G	RND3	NM_001254738,NM_005168	.
2	151343978	GAAAAA	G	RND3	NM_005168	.
2	152402513	C	CT	NEB	NM_001164507,NM_001164508,NM_001271208,NM_004543	.
2	160958349	GAA	G	ITGB6	NM_000888,NM_001282353,NM_001282354,NM_001282355,NM_001282388,NM_001282389,NM_001282390	.
2	162876813	G	GTT	DPP4	NM_001935	.
2	179542570	TTTC	T	TTN	NM_001256850,NM_001267550,NM_133378	TTN:NM_133378:exon143:c.30334_30336del:p.10112_10112del,TTN:NM_001256850:exon144:c.33115_33117del:p.11039_11039del,TTN:NM_001267550:exon146:c.34066_34068del:p.11356_11356del
2	206364764	A	ATT	PARD3B	NM_001302769,NM_057177,NM_152526,NM_205863	.
2	218712886	GGCT	G	TNS1	NM_022648	TNS1:NM_022648:exon17:c.1976_1978del:p.659_660del
2	231861032	TCAGCAGCCTAGCCCTGAATCCACACCA	T	SPATA3	NM_139073	SPATA3:NM_139073:exon1:c.85_111del:p.29_37del
2	233712226	C	CGCA	GIGYF2	NM_001103146,NM_001103147,NM_001103148,NM_015575	GIGYF2:NM_001103148:exon26:c.3611_3612insGCA:p.P1204delinsPQ,GIGYF2:NM_001103146:exon27:c.3629_3630insGCA:p.P1210delinsPQ,GIG

						YF2:NM_001103147:exon29:c.3692_3693insGCA:p.P1231delinsPQ,GIGYF2:NM_015575:exon29:c.3629_3630insGCA:p.P1210delinsPQ
2	241696837	CTCA	C	KIF1A	NM_001244008	KIF1A:NM_001244008:exon27:c.2754_2756del:p.918_919del
2	241696840	ATCC	A	KIF1A	NM_001244008	KIF1A:NM_001244008:exon27:c.2751_2753del:p.917_918del
3	1339680	GGTTTTT	G	CNTN6	NM_001289080,NM_001289081,NM_014461	.
3	12942850	GC	G	IQSEC1	NM_001134382	.
3	24270500	G	GA	THRB	NM_000461,NM_001128176,NM_001128177,NM_001252634	.
3	27763427	G	GCGC	EOMES	NM_001278182,NM_005442	EOMES:NM_001278182:exon1:c.358_359insGCG:p.A120delinsGA,EOMES:NM_005442:exon1:c.358_359insGCG:p.A120delinsGA
3	33907835	T	TCTCC	PDCD6IP	NM_001162429,NM_013374	.
3	42251577	C	CGGA	TRAK1	NM_001265608,NM_001265609,NM_014965	TRAK1:NM_001265609:exon13:c.1841_1842insGGA:p.T614delinsTE,TRAK1:NM_014965:exon13:c.1889_1890insGGA:p.T630delinsTE,TRAK1:NM_001265608:exon14:c.2063_2064insGGA:p.T688delinsTE
3	42251577	C	CGGAGGA	TRAK1	NM_001265608,NM_001265609,NM_014965	TRAK1:NM_001265609:exon13:c.1841_1842insGGAGGA:p.T614delinsTEE,TRAK1:NM_014965:exon13:c.1889_1890insGGAGGA:p.T630delinsTEE,TRAK1:NM_001265608:exon14:c.2063_2064insGGAGGA:p.T688delinsTEE
3	49395673	GGCCGCC	G	GPX1	NM_000581,NM_201397	UNKNOWN
3	50329644	T	TGC	IFRD2	NM_006764	.
3	53910109	T	TG	ACTR8	NM_022899	.
3	54959058	AT	A	LRTM1	NM_020678	LRTM1:NM_020678:exon2:c.191delA:p.H64fs
3	56650051	A	ACTT	CCDC66	NM_001012506,NM_001141947	CCDC66:NM_001012506:exon13:c.1711_1712insCTT:p.T571delinsTS,CCDC66:NM_001141947:exon13:c.1813_1814insCTT:p.T605delinsTS

3	56650054	T	TCTC	CCDC66	NM_001012506,NM_001141947	CCDC66:NM_001012506:exon13:c.1714_1715insCTC:p.S572delinsSP,CCDC66:NM_001141947:exon13:c.1816_1817insCTC:p.S606delinsSP
3	57291464	A	AT	APPL1	NM_012096	.
3	65425560	T	TCTG	MAGI1	NM_001033057,NM_004742,NM_015520	MAGI1:NM_001033057:exon9:c.1263_1264insCAG:p.T422delinsQT,MAGI1:NM_004742:exon9:c.1263_1264insCAG:p.T422delinsQT,MAGI1:NM_015520:exon9:c.1263_1264insCAG:p.T422delinsQT
3	75786555	C	CTG	ZNF717	NM_001128223,NM_001290208,NM_001290209	ZNF717:NM_001128223:exon5:c.2218_2219insCA:p.C740fs,ZNF717:NM_001290208:exon5:c.2218_2219insCA:p.C740fs,ZNF717:NM_001290209:exon5:c.2068_2069insCA:p.C690fs
3	75786817	CACATTCATT	C	ZNF717	NM_001128223,NM_001290208,NM_001290209	ZNF717:NM_001128223:exon5:c.1948_1956del:p.650_652del,ZNF717:NM_001290208:exon5:c.1948_1956del:p.650_652del,ZNF717:NM_001290209:exon5:c.1798_1806del:p.600_602del
3	75787645	GAA	G	ZNF717	NM_001128223,NM_001290208,NM_001290209	ZNF717:NM_001128223:exon5:c.1127_1128del:p.F376fs,ZNF717:NM_001290208:exon5:c.1127_1128del:p.F376fs,ZNF717:NM_001290209:exon5:c.977_978del:p.F326fs
3	107097080	CAAATG	C	CCDC54	NM_032600	CCDC54:NM_032600:exon1:c.647_651del:p.Q216fs
3	108179219	CA	C	MYH15	NM_014981	.
3	108754320	T	TAA	MORC1	NM_014429	.
3	113003419	C	CTT	BOC	NM_001301861,NM_033254	.
3	113376110	TTGC	T	KIAA2018	NM_001009899	KIAA2018:NM_001009899:exon7:c.4416_4418del:p.1472_1473del
3	113376110	TTGCTGC	T	KIAA2018	NM_001009899	KIAA2018:NM_001009899:exon7:c.4413_4418del:p.1471_1473del
3	113376110	TTGCTGCTGC	T	KIAA2018	NM_001009899	KIAA2018:NM_001009899:exon7:c.4410_4418del:p.1470_1473del
3	124066121	C	CTGTG	KALRN	NM_001024660,NM_003947	.
3	124066121	C	CTGTGTG	KALRN	NM_001024660,NM_003947	.

			TG			
3	124515240	CAGA	C	ITGB5	NM_002213	ITGB5:NM_002213:exon10:c.1685_1687del:p.562_563del
3	124998098	C	CAAA	ZNF148	NM_021964	.
3	130290036	G	GCTGATAAA	COL6A6	NM_001102608	COL6A6:NM_001102608:exon6:c.2776_2777insCTGATAAA:p.A926fs
3	158537427	TTTTTA	T	MFSD1	NM_001167903,NM_001289406,NM_001289407,NM_022736	.
3	158537431	TA	T	MFSD1	NM_001167903,NM_001289406,NM_001289407,NM_022736	.
3	167747641	C	CCTG	GOLIM4	NM_014498	GOLIM4:NM_014498:exon10:c.1359_1360insCAG:p.V454delinsQV
3	183493743	CGGA	C	YEATS2	NM_018023	YEATS2:NM_018023:exon18:c.2410_2412del:p.804_804del
3	183493743	C	CGGA	YEATS2	NM_018023	YEATS2:NM_018023:exon18:c.2409_2410insGGA:p.A803delinsAG
3	184429133	A	ATCC	MAGEF1	NM_022149	MAGEF1:NM_022149:exon1:c.476_477insGGA:p.D159delinsED
3	195792300	GGGGC	G	TFRC	NM_001128148,NM_003234	.
3	197880130	GGCAGCAGCA	G	FAM157A	NM_001145248	FAM157A:NM_001145248:exon2:c.210_218del:p.70_73del
3	197880130	GGCAGCA	G	FAM157A	NM_001145248	FAM157A:NM_001145248:exon2:c.210_215del:p.70_72del
4	1019054	CCA	C	FGFRL1	NM_001004356,NM_001004358,NM_021923	FGFRL1:NM_021923:exon6:c.1435_1436del:p.H479fs,FGFRL1:NM_001004356:exon7:c.1435_1436del:p.H479fs,FGFRL1:NM_001004358:exon7:c.1435_1436del:p.H479fs
4	3076603	CCAGCAGCAG	C	HTT	NM_002111	HTT:NM_002111:exon1:c.52_60del:p.18_20del
4	3076665	A	AGCCGCCCCC	HTT	NM_002111	HTT:NM_002111:exon1:c.113_114insGCCGCCCCC:p.Q38delinsQPPP
4	3076672	A	ACCGCC	HTT	NM_002111	HTT:NM_002111:exon1:c.120_121insCCGCCGC

			GCCG			CG:p.P40delinsPPPP
4	17634256	C	CAA	FAM184B	NM_015688	.
4	36162273	C	CA	ARAP2	NM_015230	.
4	104007666	TTGA	T	BDH2	NM_020139	BDH2:NM_020139:exon6:c.386_388del:p.129_130del
4	147560457	T	TGGC	POU4F2	NM_004575	POU4F2:NM_004575:exon1:c.165_166insGGC:p.G55delinsGG
4	155244401	TTTTG	T	DCHS2	NM_001142552	DCHS2:NM_001142552:exon8:c.4095_4098del:p.N1365fs
4	186435871	A	ATT	PDLIM3	NM_001114107	PDLIM3:NM_001114107:exon4:c.503_504insAA:p.P168fs
5	56177848	TCAA	T	MAP3K1	NM_005921	MAP3K1:NM_005921:exon14:c.2822_2824del:p.941_942del
5	68396764	T	TG	SLC30A5	NM_022902,NM_024055	.
5	79028932	TC	T	CMYA5	NM_153610	CMYA5:NM_153610:exon2:c.4345delC:p.P1449fs
5	79950724	G	GCCGCAGCGC	MSH3	NM_002439	MSH3:NM_002439:exon1:c.178_179insCCGCAGCGC:p.A60delinsAAAP
5	90002217	ACT	A	GPR98	NM_032119	.
5	90002219	TC	T	GPR98	NM_032119	.
5	90002220	C	CT	GPR98	NM_032119	.
5	111500816	C	CTAAAA	EPB41L4A	NM_022140	.
5	112824027	T	TGCTGCTGCTGCC	MCC	NM_001085377	MCC:NM_001085377:exon1:c.84_85insGGCAGCAGCAGC:p.S29delinsGSSSS
5	113698631	T	TGCC	KCNN2	NM_021614	KCNN2:NM_021614:exon1:c.159_160insGCC:p.A53delinsAA
5	115338955	C	CAA	AQPEP	NM_173800	LVRN:NM_173800:exon12:c.1915_1916insAA:p.Q639fs
5	139931628	A	AGTC	SRA1	NM_001035235	SRA1:NM_001035235:exon3:c.328_329insGAC:p.V110delinsGL
5	140223109	GGCAA	G	PCDHA8	NM_018911,NM_031856	PCDHA8:NM_018911:exon1:c.2204_2207del:p.G735fs,PCDHA8:NM_031856:exon1:c.2204_2207del:p.G735fs

5	140953540	C	CAA	DIAPH1	NM_001079812,NM_005219	DIAPH1:NM_001079812:exon15:c.1849_1850insTT:p.C617fs,DIAPH1:NM_005219:exon16:c.1876_1877insTT:p.C626fs
5	158588633	GAAAAA	G	RNF145	NM_001199380,NM_001199381,NM_001199382,NM_001199383,NM_144726	.
5	167881029	TGGA	T	WWC1	NM_001161661,NM_001161662,NM_015238	WWC1:NM_001161661:exon18:c.2583_2585del:p.861_862del,WWC1:NM_001161662:exon18:c.2583_2585del:p.861_862del,WWC1:NM_015238:exon18:c.2583_2585del:p.861_862del
5	175811094	C	CGT	NOP16	NM_001256539,NM_001256540,NM_001291305	NOP16:NM_001256539:exon5:c.586_587insAC:p.R196fs,NOP16:NM_001256540:exon5:c.583_584insAC:p.R195fs,NOP16:NM_001291305:exon5:c.544_545insAC:p.R182fs
5	175811094	C	CAT	NOP16	NM_001256539,NM_001256540,NM_001291305	NOP16:NM_001256539:exon5:c.586_587insAT:p.R196fs,NOP16:NM_001256540:exon5:c.583_584insAT:p.R195fs,NOP16:NM_001291305:exon5:c.544_545insAT:p.R182fs
5	176026119	TCTCAAAGACCCAGGATCCTCCTTC	T	GPRIN1	NM_052899	GPRIN1:NM_052899:exon2:c.693_716del:p.231_239del
5	179306693	C	CAA	TBC1D9B	NM_015043,NM_198868	TBC1D9B:NM_015043:exon8:c.1350_1351insTT:p.A451fs,TBC1D9B:NM_198868:exon8:c.1350_1351insTT:p.A451fs
6	1612017	A	ACGG	FOXC1	NM_001453	FOXC1:NM_001453:exon1:c.1337_1338insCGG:p.H446delinsHG
6	16327864	G	GTGC	ATXN1	NM_000332,NM_001128164	ATXN1:NM_001128164:exon7:c.677_678insGCA:p.H226delinsQH,ATXN1:NM_000332:exon8:c.677_678insGCA:p.H226delinsQH
6	16327864	GTGCTGCTGCTGC	G	ATXN1	NM_000332,NM_001128164	ATXN1:NM_001128164:exon7:c.666_677del:p.222_226del,ATXN1:NM_000332:exon8:c.666_677del:p.222_226del
6	29760352	CGCGGGCGCCGTGGATGG	C	LOC554223	NM_001207043	LOC554223:NM_001207043:exon2:c.438_458del:p.146_153del

		AGCA				
6	32053856	G	GC	TNXB	NM_019105	TNXB:NM_019105:exon7:c.2818_2819insG:p.P940fs
6	32191658	TAGCAGCAGCAGC	T	NOTCH4	NM_004557	NOTCH4:NM_004557:exon1:c.36_47del:p.12_16del
6	32369586	GAA	G	BTNL2	NM_001304561,NM_019602	.
6	33287880	GCCT	G	DAXX	NM_001141969,NM_001141970,NM_001254717,NM_001350	DAXX:NM_001254717:exon4:c.1145_1147del:p.382_383del,DAXX:NM_001141969:exon5:c.1370_1372del:p.457_458del,DAXX:NM_001141970:exon5:c.1406_1408del:p.469_470del,DAXX:NM_001350:exon5:c.1370_1372del:p.457_458del
6	33412257	C	CAA	SYNGAP1	NM_006772	SYNGAP1:NM_006772:exon16:c.3445_3446insAA:p.P1149fs
6	34735676	C	CCTCAT	SNRPC	NM_003093	.
6	34857302	G	GGGCGGC	ANKS1A	NM_015245	ANKS1A:NM_015245:exon1:c.123_124insGGCGGC:p.G41delinsGGG
6	35108989	TC	T	TCP11	NM_001093728,NM_001261817,NM_001261818,NM_001261819	TCP11:NM_001093728:exon1:c.17delG:p.G6fs,TCP11:NM_001261817:exon1:c.17delG:p.G6fs,TCP11:NM_001261818:exon1:c.17delG:p.G6fs,TCP11:NM_001261819:exon1:c.17delG:p.G6fs
6	42989413	CGCCGGG	C	RRP36	NM_033112	RRP36:NM_033112:exon1:c.22_27del:p.8_9del
6	46830665	C	CAA	GPR116	NM_001098518,NM_015234	ADGRF5:NM_001098518:exon15:c.2158_2159insTT:p.C720fs,ADGRF5:NM_015234:exon15:c.2158_2159insTT:p.C720fs
6	73331983	A	AGCG	KCNQ5	NM_001160130,NM_001160132,NM_001160133,NM_001160134,NM_019842	KCNQ5:NM_001160130:exon1:c.66_67insGCG:p.A22delinsAA,KCNQ5:NM_001160132:exon1:c.66_67insGCG:p.A22delinsAA,KCNQ5:NM_001160133:exon1:c.66_67insGCG:p.A22delinsAA,KCNQ5:NM_001160134:exon1:c.66_67insGCG:p.A22delinsAA,KCNQ5:NM_019842:exon1:c.66_67insGCG:p.A22delinsAA
6	96034869	G	GTATA	MANEA	NM_024641	.
6	96034869	G	GTA	MANEA	NM_024641	.

6	106987291	G	GC	AIM1	NM_001624	AIM1:NM_001624:exon7:c.3508_3509insC:p.E1170fs
6	108243119	GAAAA	G	SEC63	NM_007214	.
6	108243119	GAAAAAC	G	SEC63	NM_007214	.
6	108243122	AAAC	A	SEC63	NM_007214	.
6	109557959	C	CT	LOC100996634	NM_001277339	LOC100996634:NM_001277339:exon1:c.96_97insT:p.H32fs
6	116600470	A	ACCC	TSPYL1	NM_003309	TSPYL1:NM_003309:exon1:c.523_524insGGG:p.V175delinsGV
6	123837327	CCTTTTT	C	TRDN	NM_001251987,NM_001256020,NM_001256021,NM_006073	TRDN:NM_001251987:exon6:c.503_508del:p.168_170del,TRDN:NM_001256020:exon6:c.503_508del:p.168_170del,TRDN:NM_001256021:exon6:c.503_508del:p.168_170del,TRDN:NM_006073:exon6:c.503_508del:p.168_170del
6	132168996	G	GGTGT	ENPP1	NM_006208	.
6	132203474	TC	T	ENPP1	NM_006208	.
6	146480740	TTCTC	T	GRM1	NM_001278064,NM_001278065,NM_001278066,NM_001278067	.
6	150174133	C	CAA	LRP11	NM_032832	.
6	151766572	C	CAA	RMND1	NM_017909	RMND1:NM_017909:exon2:c.374_375insTT:p.R125fs
6	157099426	ACAG	A	ARID1B	NM_017519,NM_020732	ARID1B:NM_017519:exon1:c.364_366del:p.122_122del,ARID1B:NM_020732:exon1:c.364_366del:p.122_122del
6	161519350	CCTG	C	MAP3K4	NM_001291958,NM_001301072,NM_005922	MAP3K4:NM_001301072:exon17:c.3554_3556del:p.1185_1186del,MAP3K4:NM_005922:exon17:c.3566_3568del:p.1189_1190del,MAP3K4:NM_001291958:exon18:c.1925_1927del:p.642_643del
6	170871013	A	ACAG	TBP	NM_001172085,NM_003194	TBP:NM_001172085:exon2:c.129_130insCAG:p.Q43delinsQQ,TBP:NM_003194:exon3:c.189_190insCAG:p.Q63delinsQQ
6	170871037	GCAA	G	TBP	NM_001172085,NM_003194	TBP:NM_001172085:exon2:c.154_156del:p.52_52del,TBP:NM_003194:exon3:c.214_216del:p.72_72

						del
6	170871046	ACAGCAGCAG	A	TBP	NM_001172085,NM_003194	TBP:NM_001172085:exon2:c.163_171del:p.55_57del,TBP:NM_003194:exon3:c.223_231del:p.75_77del
7	1586653	A	AGCC	TMEM184A	NM_001097620	TMEM184A:NM_001097620:exon9:c.1176_1177insGGC:p.S393delinsGS
7	2515382	G	GC	GRIFIN	NM_001291784	UNKNOWN
7	2552881	A	AGATG	LFNG	NM_001166355	LFNG:NM_001166355:exon2:c.138_139insGATG:p.T46fs
7	15725797	ATGG	A	MEOX2	NM_005924	MEOX2:NM_005924:exon1:c.228_230del:p.76_77del
7	19184751	C	CTTA	FERD3L	NM_152898	FERD3L:NM_152898:exon1:c.234_235insTAA:p.E79delinsX
7	22190131	GAA	G	RAPGEF5	NM_012294	.
7	23854838	G	GA	STK31	NM_001260504,NM_031414,NM_032944	.
7	30486579	T	TGC	NOD1	NM_006092	.
7	43540809	C	CAA	HECW1	NM_001287059,NM_015052	.
7	47479119	C	CAA	TNS3	NM_022748	TNS3:NM_022748:exon6:c.115_116insTT:p.R39fs
7	51098567	GTCT	G	COBL	NM_001287436,NM_015198	COBL:NM_015198:exon9:c.1443_1445del:p.481_482del,COBL:NM_001287436:exon10:c.1614_1616del:p.538_539del
7	54614698	C	CG	VSTM2A	NM_001301009,NM_182546	.
7	64169016	T	TGAA	ZNF107	NM_001013746,NM_001282359,NM_001282360,NM_016220	ZNF107:NM_001282360:exon3:c.2445_2446insGAA:p.C815delinsCE,ZNF107:NM_001282359:exon4:c.2541_2542insGAA:p.C847delinsCE,ZNF107:NM_001013746:exon5:c.2334_2335insGAA:p.C778delinsCE,ZNF107:NM_016220:exon7:c.2334_2335insGAA:p.C778delinsCE
7	94292825	ACAACTC	A	PEG10	NM_001172437,NM_001172438,NM_001184961,NM_001184962	PEG10:NM_001172438:exon2:c.186_191del:p.62_64del,PEG10:NM_001184962:exon2:c.60_65del:p.20_22del

7	102038153	CTGTG	C	PRKRIP1	NM_024653	.
7	102038153	CTGTGTG	C	PRKRIP1	NM_024653	.
7	123672456	C	CGCT	TMEM229A	NM_001136002	TMEM229A:NM_001136002:exon1:c.601_602insAGC:p.R201delinsQR
7	137092745	CA	C	DGKI	NM_004717	.
7	151945071	G	GT	KMT2C	NM_170606	KMT2C:NM_170606:exon14:c.2447dupA:p.Y816_I817delinsX
8	10467629	T	TTTC	RP1L1	NM_178857	RP1L1:NM_178857:exon4:c.3978_3979insGAA:p.T1327delinsET
8	11666218	GTCCCAC	G	FDFT1	NM_001287750	FDFT1:NM_001287750:exon1:c.193_198del:p.65_66del
8	22458392	C	CAA	C8orf58	NM_001013842,NM_001198827,NM_173686	.
8	24350102	TGAG	T	ADAM7	NM_003817	ADAM7:NM_003817:exon15:c.1648_1650del:p.550_550del
8	37791988	GT	G	GOT1L1	NM_152413	GOT1L1:NM_152413:exon9:c.1088delA:p.Y363fs
8	59514103	C	CAAA	NSMAF	NM_001144772,NM_003580	.
8	77765298	ACCT	A	ZFHX4	NM_024721	ZFHX4:NM_024721:exon10:c.6142_6144del:p.2048_2048del
8	90937720	ATTTG	A	OSGIN2	NM_001126111,NM_004337	OSGIN2:NM_001126111:exon6:c.1611_1614del:p.H537fs,OSGIN2:NM_004337:exon6:c.1479_1482del:p.H493fs
8	101725026	C	CAA	PABPC1	NM_002568	.
8	144511953	ATGG	A	MAFA	NM_201589	MAFA:NM_201589:exon1:c.621_623del:p.207_208del
8	144652404	C	CCTCCTCACA	MROH6	NM_001100878	.
8	144671407	TTGCGGCCCCGCCGGTCTC	T	EEF1D	NM_001130053,NM_032378	EEF1D:NM_001130053:exon3:c.827_844del:p.276_282del,EEF1D:NM_032378:exon3:c.827_844del:p.276_282del
9	2039776	ACAG	A	SMARCA2	NM_001289396,NM_001289397,NM_003070,NM_139045	SMARCA2:NM_001289396:exon4:c.667_669del:p.223_223del,SMARCA2:NM_001289397:exon4:c.

						667_669del:p.223_223del,SMARCA2:NM_003070:exon4:c.667_669del:p.223_223del,SMARCA2:NM_139045:exon4:c.667_669del:p.223_223del
9	12775861	T	TGGCGGCGGC	LURAP1L	NM_203403	LURAP1L:NM_203403:exon1:c.147_148insGGCGGCGGC:p.G49delinsGGGG
9	12775861	T	TGGCGGC	LURAP1L	NM_203403	LURAP1L:NM_203403:exon1:c.147_148insGGCGGC:p.G49delinsGGG
9	18826273	TC	T	ADAMTSL1	NM_001040272	.
9	33056971	GA	G	SMU1	NM_018225	.
9	34637689	C	CTT	SIGMAR1	NM_001282205,NM_001282207,NM_001282208,NM_001282209,NM_005866,NM_147157	SIGMAR1:NM_001282205:exon1:c.5_6insAA:p.Q2fs,SIGMAR1:NM_001282207:exon1:c.5_6insAA:p.Q2fs,SIGMAR1:NM_001282208:exon1:c.5_6insAA:p.Q2fs,SIGMAR1:NM_001282209:exon1:c.5_6insAA:p.Q2fs,SIGMAR1:NM_005866:exon1:c.5_6insAA:p.Q2fs,SIGMAR1:NM_147157:exon1:c.5_6insAA:p.Q2fs
9	78936530	C	CTT	PCSK5	NM_001190482	PCSK5:NM_001190482:exon30:c.3996_3997insTT:p.D1332fs
9	95237024	CTCA	C	ASPN	NM_001193335,NM_017680	ASPN:NM_001193335:exon2:c.153_155del:p.51_52del,ASPN:NM_017680:exon2:c.153_155del:p.51_52del
9	95237024	CTCATCA	C	ASPN	NM_001193335,NM_017680	ASPN:NM_001193335:exon2:c.150_155del:p.50_52del,ASPN:NM_017680:exon2:c.150_155del:p.50_52del
9	96214721	CT	C	FAM120AOS	NM_198841	FAM120AOS:NM_198841:exon1:c.270delA:p.I90fs
9	98678725	C	CTT	ERCC6L2	NM_001010895,NM_020207	.
9	100616700	AGCCGCC	A	FOXE1	NM_004473	FOXE1:NM_004473:exon1:c.505_510del:p.169_170del
9	115932150	T	TTTC	FKBP15	NM_015258	FKBP15:NM_015258:exon26:c.2838_2839insGAA:p.K947delinsEK
9	116346117	C	CAGG	RGS3	NM_001276260,NM_001282922,NM_001282923,NM_130795,NM_144488	RGS3:NM_001276260:exon2:c.388_389insAGG:p.R130delinsQG,RGS3:NM_001282922:exon3:c.38

						8_389insAGG:p.R130delinsQG,RGS3:NM_130795:exon11:c.1582_1583insAGG:p.R528delinsQG,RGS3:NM_001282923:exon18:c.2095_2096insAGG:p.R699delinsQG,RGS3:NM_144488:exon21:c.2425_2426insAGG:p.R809delinsQG
9	131020792	ACCG	A	GOLGA2	NM_004486	GOLGA2:NM_004486:exon21:c.2147_2149del:p.716_717del
9	139137538	C	CAA	QSOX2	NM_181701	QSOX2:NM_181701:exon1:c.111_112insTT:p.V38fs
9	141107536	G	GGCA	FAM157B	NM_001145249	FAM157B:NM_001145249:exon2:c.218_219insGCA:p.R73delinsRQ
9	141107536	GGCAGCAGCAGCA	G	FAM157B	NM_001145249	FAM157B:NM_001145249:exon2:c.219_230del:p.73_77del
10	13658408	C	CAA	PRPF18	NM_003675	PRPF18:NM_003675:exon9:c.803_804insAA:p.A268fs
10	24721971	C	CAA	KIAA1217	NM_001098500,NM_001282767,NM_001282768,NM_019590	KIAA1217:NM_001282767:exon4:c.601_602insAA:p.P201fs,KIAA1217:NM_001282768:exon4:c.601_602insAA:p.P201fs,KIAA1217:NM_019590:exon4:c.601_602insAA:p.P201fs,KIAA1217:NM_001098500:exon5:c.361_362insAA:p.P121fs
10	72201224	T	TGC	NODAL	NM_018055	.
10	76788659	GGAAGAGGAA	G	KAT6B	NM_001256468,NM_001256469,NM_012330	KAT6B:NM_001256468:exon18:c.3529_3537del:p.1177_1179del,KAT6B:NM_001256469:exon18:c.3202_3210del:p.1068_1070del,KAT6B:NM_012330:exon18:c.4078_4086del:p.1360_1362del
10	82331344	C	CTT	SH2D4B	NM_001145719,NM_207372	.
10	92678738	AAAATAAAT	A	ANKRD1	NM_014391	.
10	92678738	AAAAT	A	ANKRD1	NM_014391	.
10	95109602	GTCT	G	MYOF	NM_013451,NM_133337	MYOF:NM_133337:exon35:c.4004_4006del:p.1335_1336del,MYOF:NM_013451:exon36:c.4043_4045del:p.1348_1349del
10	97146882	G	GA	SORBS1	NM_001034955	.
10	99126521	G	GCTCCTC	RRP12	NM_001145114,NM_001284337,NM_0	RRP12:NM_001284337:exon24:c.2892_2893insG

			CTCCTC		15179	AGGAGGAGGAG:p.P965delinsEEEEP,RRP12:NM_001145114:exon25:c.3009_3010insGAGGAGGAGGAG:p.P1004delinsEEEEP,RRP12:NM_015179:exon27:c.3192_3193insGAGGAGGAGGAG:p.P1065delinsEEEEP
10	115405703	G	GAA	NRAP	NM_001261463,NM_006175,NM_198060	.
10	115804725	G	GCCCTCA	ADRB1	NM_000684	ADRB1:NM_000684:exon1:c.834_835insCCCTCA:p.S278delinsSPS
10	116085784	C	CCCG	AFAP1L2	NM_001287824	AFAP1L2:NM_001287824:exon5:c.330_331insCGG:p.G111delinsRG
10	135203260	C	CA	PAOX	NM_152911	.
10	135236958	CGCCGCCCCGGCT	C	SPRN	NM_001012508	SPRN:NM_001012508:exon2:c.216_227del:p.72_76del
11	801631	T	TG	PIDD1	NM_145886,NM_145887	.
11	4592706	T	TAC	C11orf40	NM_144663	C11orf40:NM_144663:exon4:c.600_601insGT:p.M201fs
11	4592708	C	CAG	C11orf40	NM_144663	C11orf40:NM_144663:exon4:c.598_599insCT:p.C200fs
11	6411935	TGCTGGC	T	SMPD1	NM_000543,NM_001007593	SMPD1:NM_000543:exon1:c.108_113del:p.36_38del,SMPD1:NM_001007593:exon1:c.108_113del:p.36_38del
11	6637739	ATTTT	A	TPP1	NM_000391	.
11	6662745	C	CCAG	DCHS1	NM_003737	DCHS1:NM_003737:exon2:c.99_100insCTG:p.G34delinsLG
11	20404518	TA	T	HTATIP2	NM_001098520,NM_001098521,NM_001098522,NM_006410	.
11	20623218	AGG	A	SLC6A5	NM_004211	.
11	49076963	C	CAA	TRIM64C	NM_001206631	.
11	51412326	C	CAA	OR4A5	NM_001005272	OR4A5:NM_001005272:exon1:c.69_70insTT:p.A24fs
11	66083170	TGAG	T	CD248	NM_020404	CD248:NM_020404:exon1:c.1326_1328del:p.442_

						443del
11	66512290	G	GGGC	C11orf80	NM_024650	C11orf80:NM_024650:exon1:c.77_78insGGC:p.G26delinsGA
11	72319709	T	TG	PDE2A	NM_001143839,NM_001146209,NM_001243784,NM_002599	PDE2A:NM_001143839:exon2:c.209_210insC:p.R70fs,PDE2A:NM_001243784:exon3:c.167_168insC:p.R56fs,PDE2A:NM_002599:exon3:c.230_231insC:p.R77fs,PDE2A:NM_001146209:exon4:c.203_204insC:p.R68fs
11	72946250	TAGA	T	P2RY2	NM_002564,NM_176071,NM_176072	P2RY2:NM_002564:exon3:c.1047_1049del:p.349_350del,P2RY2:NM_176071:exon3:c.1047_1049del:p.349_350del,P2RY2:NM_176072:exon3:c.1047_1049del:p.349_350del
11	102738793	G	GT	MMP12	NM_002426	UNKNOWN
11	118939939	T	TC	VPS11	NM_001290185,NM_021729	UNKNOWN
12	2062323	T	TTGC	DCP1B	NM_152640	DCP1B:NM_152640:exon7:c.782_783insGCA:p.Q261delinsQQ
12	7045891	A	ACAGCAG	ATN1	NM_001007026,NM_001940	ATN1:NM_001007026:exon5:c.1461_1462insCAGCAG:p.Q487delinsQQQ,ATN1:NM_001940:exon5:c.1461_1462insCAGCAG:p.Q487delinsQQQ
12	7045891	A	ACAGCAGCAGCAG	ATN1	NM_001007026,NM_001940	ATN1:NM_001007026:exon5:c.1461_1462insCAGCAGCAGCAG:p.Q487delinsQQQQQ,ATN1:NM_001940:exon5:c.1461_1462insCAGCAGCAGCAG:p.Q487delinsQQQQQ
12	7521563	TGA	T	CD163L1	NM_001297650,NM_174941	.
12	8757521	T	TA	AICDA	NM_020661	.
12	14720554	GGCA	G	PLBD1	NM_024829	PLBD1:NM_024829:exon1:c.74_76del:p.25_26del
12	15834239	G	GTAA	EPS8	NM_004447	.
12	49091968	T	TA	CCNT1	NM_001240,NM_001277842	.
12	49993819	AC	A	FAM186B	NM_032130	FAM186B:NM_032130:exon4:c.1603delG:p.V535fs
12	50232334	C	CT	BCDIN3D	NM_181708	BCDIN3D:NM_181708:exon2:c.698dupA:p.Q233fs

12	52800022	TGC	T	KRT82	NM_033033	KRT82:NM_033033:exon1:c.38_39del:p.G13fs
12	53189413	ACCAAAGCCACCAGCCCCT	A	KRT3	NM_057088	KRT3:NM_057088:exon1:c.396_413del:p.132_138del
12	53449383	C	CAA	TENC1	NM_015319,NM_170754,NM_198316	TNS2:NM_015319:exon9:c.635_636insAA:p.A212fs,TNS2:NM_170754:exon9:c.605_606insAA:p.A202fs,TNS2:NM_198316:exon9:c.233_234insAA:p.A78fs
12	56863284	C	CCG	SPRYD4	NM_207344	SPRYD4:NM_207344:exon2:c.547_548insCG:p.P183fs
12	76424937	TTGC	T	PHLDA1	NM_007350	PHLDA1:NM_007350:exon1:c.582_584del:p.194_195del
12	91347581	C	CTCT	CCER1	NM_152638	CCER1:NM_152638:exon1:c.938_939insAGA:p.E313delinsEE
12	118198812	T	TGC	KSR2	NM_173598	.
12	118506324	ATCTTCC	A	VSIG10	NM_019086	VSIG10:NM_019086:exon8:c.1419_1424del:p.473_475del
12	121678327	C	CTTT	CAMKK2	NM_001270486	CAMKK2:NM_001270486:exon16:c.1614_1615insAAA:p.G539delinsKG
12	122064769	GGGCCCC	G	ORAI1	NM_032790	ORAI1:NM_032790:exon1:c.123_128del:p.41_43del
12	122359397	G	GGAGGAGGAGGAGAAA	WDR66	NM_001178003,NM_144668	WDR66:NM_001178003:exon2:c.186_187insGAGGAGGAGGAGAAA:p.G62delinsGEEEEK,WDR66:NM_144668:exon2:c.186_187insGAGGAGGAGGAGAAA:p.G62delinsGEEEEK
12	125478381	C	CCTG	BRI3BP	NM_080626	BRI3BP:NM_080626:exon1:c.45_46insCTG:p.L15delinsLL
13	32360777	A	AGC	RXFP2	NM_001166058,NM_130806	.
13	38932224	ATTTAG	A	UFM1	NM_001286703,NM_001286704,NM_001286705,NM_001286706,NM_016617	.
13	40298638	TA	T	COG6	NM_001145079,NM_020751	.
13	43462431	G	GATTTC	EPSTI1	NM_001002264	EPSTI1:NM_001002264:exon13:c.1187_1188insG

						AAAT:p.L396fs
13	53029770	CGCGGTGGCGGTGGCGGTG	C	CKAP2	NM_001098525,NM_001286687,NM_018204	.
13	72440658	TGCCGCC	T	DACH1	NM_004392,NM_080759,NM_080760	DACH1:NM_004392:exon1:c.244_249del:p.82_83del,DACH1:NM_080759:exon1:c.244_249del:p.82_83del,DACH1:NM_080760:exon1:c.244_249del:p.82_83del
13	78272267	T	TGG	SLAIN1	NM_001242868	SLAIN1:NM_001242868:exon1:c.219_220insGG:p.A73fs
13	98658610	ATTTTTTTTTTTTTTTTT	A	IPO5	NM_002271	.
13	98658610	ATTTTTTTTTTTTTTTTTT	A	IPO5	NM_002271	.
13	98658610	ATTTTTTTTTTTTTTTTTTT	A	IPO5	NM_002271	.
13	100635034	A	ACCC	ZIC2	NM_007129	ZIC2:NM_007129:exon1:c.716_717insCCC:p.H239delinsHP
13	113973924	A	ATT	LAMP1	NM_005561	LAMP1:NM_005561:exon5:c.703_704insTT:p.S235fs
14	20666175	C	CA	OR11G2	NM_001005503	OR11G2:NM_001005503:exon1:c.682dupA:p.C227fs
14	23371282	CAGT	C	RBM23	NM_001077351,NM_001077352,NM_018107	RBM23:NM_001077352:exon10:c.1048_1050del:p.350_350del,RBM23:NM_018107:exon11:c.1102_1104del:p.368_368del,RBM23:NM_001077351:exon12:c.1150_1152del:p.384_384del
14	24646406	G	GGAA	REC8	NM_001048205,NM_005132	REC8:NM_001048205:exon8:c.681_682insGAA:p.E227delinsEE,REC8:NM_005132:exon9:c.681_682insGAA:p.E227delinsEE
14	25326229	T	TGC	STXBP6	NM_001304476,NM_001304477,NM_014178	.
14	71275773	GCCT	G	MAP3K9	NM_001284230,NM_033141	MAP3K9:NM_001284230:exon1:c.113_115del:p.38_39del,MAP3K9:NM_033141:exon1:c.113_115de

						l:p.38_39del
14	77493644	CGCAGCGGCGGCGGCG	C	IRF2BPL	NM_024496	IRF2BPL:NM_024496:exon1:c.477_491del:p.159_164del
14	77493791	CTGT	C	IRF2BPL	NM_024496	IRF2BPL:NM_024496:exon1:c.342_344del:p.114_115del
14	93154537	TGGC	T	RIN3	NM_024832	RIN3:NM_024832:exon10:c.2899_2901del:p.967_967del
14	95903307	TAGA	T	SYNE3	NM_152592	SYNE3:NM_152592:exon14:c.2385_2387del:p.795_796del
14	104570772	CTG	C	ASPG	NM_001080464	ASPG:NM_001080464:exon8:c.886_887del:p.C296fs
15	23685004	G	GCAT	GOLGA6L2	NM_001304388	GOLGA6L2:NM_001304388:exon8:c.2617_2618insATG:p.A873delinsDA
15	38776806	AGGTGGTGGT	A	FAM98B	NM_173611	FAM98B:NM_173611:exon8:c.1249_1257del:p.417_419del
15	50888582	T	TA	TRPM7	NM_001301212,NM_017672	.
15	50901929	TA	T	TRPM7	NM_001301212,NM_017672	.
15	63950739	T	TGC	HERC1	NM_003922	.
15	81592161	G	GCCT	IL16	NM_001172128,NM_004513,NM_172217	IL16:NM_004513:exon2:c.391_392insCCT:p.A131delinsAS,IL16:NM_172217:exon13:c.2494_2495insCCT:p.A832delinsAS,IL16:NM_001172128:exon14:c.2494_2495insCCT:p.A832delinsAS
15	82533666	C	CAA	EFTUD1	NM_001040610,NM_024580	EFTUD1:NM_001040610:exon3:c.169_170insTT:p.R57fs,EFTUD1:NM_024580:exon5:c.322_323insTT:p.R108fs
15	89876827	T	TTGC	POLG	NM_001126131,NM_002693	POLG:NM_001126131:exon2:c.158_159insGCA:p.Q53delinsQQ,POLG:NM_002693:exon2:c.158_159insGCA:p.Q53delinsQQ
15	100252709	CCAG	C	MEF2A	NM_001130926,NM_001130927,NM_001130928,NM_001171894,NM_005587	MEF2A:NM_001130928:exon8:c.1024_1026del:p.342_342del,MEF2A:NM_001130926:exon10:c.1228_1230del:p.410_410del,MEF2A:NM_001130927:exon10:c.1048_1050del:p.350_350del,MEF2A:NM_005587:exon11:c.1234_1236del:p.412_412del,ME

						F2A:NM_001171894:exon12:c.1228_1230del:p.410_410del
15	100252735	AGCAGCAGCC	A	MEF2A	NM_001130926,NM_001130927,NM_001130928,NM_001171894,NM_005587	MEF2A:NM_001130928:exon8:c.1050_1058del:p.350_353del,MEF2A:NM_001130926:exon10:c.1254_1262del:p.418_421del,MEF2A:NM_001130927:exon10:c.1074_1082del:p.358_361del,MEF2A:NM_005587:exon11:c.1260_1268del:p.420_423del,MEF2A:NM_001171894:exon12:c.1254_1262del:p.418_421del
15	102359306	C	CTT	OR4F15	NM_001001674	OR4F15:NM_001001674:exon1:c.917_918insTT:p.A306fs
16	138709	C	CAA	NPRL3	NM_001039476,NM_001077350,NM_001243247,NM_001243248,NM_001243249	UNKNOWN
16	2821988	TC	T	TCEB2	NM_207013	.
16	3707798	A	ATGGCCTGAGTGACCAACTGG	DNASE1	NM_005223	DNASE1:NM_005223:exon8:c.782_783insTGGCCTGAGTGACCAACTGG:p.Y261fs
16	12009530	C	CCCG	GSPT1	NM_001130006,NM_002094	GSPT1:NM_001130006:exon1:c.47_48insCGG:p.G16delinsGG,GSPT1:NM_002094:exon1:c.47_48insCGG:p.G16delinsGG
16	23713469	T	TGC	ERN2	NM_033266	.
16	24564879	GTA	G	RBBP6	NM_006910,NM_018703	.
16	48130771	CA	C	ABCC12	NM_033226	ABCC12:NM_033226:exon22:c.3080delT:p.L1027fs
16	51175643	T	TGCC	SALL1	NM_001127892,NM_002968	SALL1:NM_001127892:exon2:c.198_199insGGC:p.S67delinsGS,SALL1:NM_002968:exon2:c.489_490insGGC:p.S164delinsGS
16	51175655	C	CGCT	SALL1	NM_001127892,NM_002968	SALL1:NM_001127892:exon2:c.186_187insAGC:p.G63delinsSG,SALL1:NM_002968:exon2:c.477_478insAGC:p.G160delinsSG
16	51175663	C	CTGA	SALL1	NM_001127892,NM_002968	SALL1:NM_001127892:exon2:c.178_179insTCA:

						p.S60delinsIS,SALL1:NM_002968:exon2:c.469_470insTCA:p.S157delinsIS
16	57732067	G	GGC	DRC7	NM_001289162,NM_001289163,NM_032269	.
16	67229833	C	CAACAGT	E2F4	NM_001950	E2F4:NM_001950:exon7:c.957_958insAACAGT:p.S319delinsSNS
16	67229844	G	GTAA	E2F4	NM_001950	E2F4:NM_001950:exon7:c.968_969insTAA:p.S323delinsSN
16	67702197	G	GGAGGCA	C16orf86	NM_001012984	C16orf86:NM_001012984:exon4:c.648_649insGAGGCA:p.V216delinsVEA
16	70896015	GA	G	HYDIN	NM_001270974	HYDIN:NM_001270974:exon69:c.11712delT:p.I3904fs
16	70954703	GGCGCTCCTTCTCCGT	G	HYDIN	NM_001270974	HYDIN:NM_001270974:exon46:c.7561_7575del:p.2521_2525del
16	71956588	GTATA	G	IST1	NM_001270975,NM_001270976,NM_001270977,NM_001270978,NM_001270979,NM_014761	.
16	71956592	A	AAGTGTG	IST1	NM_001270975,NM_001270976,NM_001270977,NM_001270978,NM_001270979,NM_014761	.
16	71981414	C	CTTTG	PKD1L3	NM_181536	UNKNOWN
16	71981418	G	GT	PKD1L3	NM_181536	UNKNOWN
16	72821593	A	AGCCGCCGCC	ZFHX3	NM_001164766,NM_006885	ZFHX3:NM_001164766:exon9:c.7839_7840insGGCGGCGGC:p.S2614delinsGGGS,ZFHX3:NM_006885:exon10:c.10581_10582insGGCGGCGGC:p.S3528delinsGGGS
16	72821618	ACCG	A	ZFHX3	NM_001164766,NM_006885	ZFHX3:NM_001164766:exon9:c.7812_7814del:p.2604_2605del,ZFHX3:NM_006885:exon10:c.10554_10556del:p.3518_3519del
16	72821618	A	ACCG	ZFHX3	NM_001164766,NM_006885	ZFHX3:NM_001164766:exon9:c.7814_7815insCGG:p.G2605delinsGG,ZFHX3:NM_006885:exon10:c.10556_10557insCGG:p.G3519delinsGG
16	87637893	C	CCTG	JPH3	NM_001271604	JPH3:NM_001271604:exon2:c.430_431insCTG:p.

						P144delinsPA
16	88599699	CCTCTGG	C	ZFPM1	NM_153813	ZFPM1:NM_153813:exon10:c.1334_1339del:p.445_447del
16	88952507	C	CA	CBFA2T3	NM_005187,NM_175931	CBFA2T3:NM_175931:exon5:c.496_497insT:p.R166fs,CBFA2T3:NM_005187:exon6:c.754_755insT:p.R252fs
17	4439731	G	GA	SPNS2	NM_001124758	.
17	6928035	C	CAGA	BCL6B	NM_181844	BCL6B:NM_181844:exon4:c.717_718insAGA:p.S239delinsSR
17	7470285	CA	C	SENP3-EIF4A1,SENP3	NR_037926;NM_015670	.
17	7750177	T	TACCACCACC	KDM6B	NM_001080424	KDM6B:NM_001080424:exon9:c.752_753insACCACCACC:p.L251delinsLPPP
17	7750177	T	TACCACC	KDM6B	NM_001080424	KDM6B:NM_001080424:exon9:c.752_753insACCACC:p.L251delinsLPP
17	7918802	C	CTT	GUCY2D	NM_000180	GUCY2D:NM_000180:exon15:c.2926_2927insTT:p.R976fs
17	12903598	G	GGAGA	ELAC2	NM_001165962,NM_018127,NM_173717	.
17	17039561	CCAG	C	MPRIP	NM_015134,NM_201274	MPRIP:NM_015134:exon6:c.534_536del:p.178_179del,MPRIP:NM_201274:exon6:c.534_536del:p.178_179del
17	17039561	CCAGCAG	C	MPRIP	NM_015134,NM_201274	MPRIP:NM_015134:exon6:c.534_539del:p.178_180del,MPRIP:NM_201274:exon6:c.534_539del:p.178_180del
17	17697093	CCAG	C	RAI1	NM_030665	RAI1:NM_030665:exon3:c.832_834del:p.278_278del
17	26092619	T	TG	NOS2	NM_000625	NOS2:NM_000625:exon20:c.2369_2370insC:p.R790fs
17	35601496	A	AT	ACACA	NM_198834,NM_198836,NM_198837,NM_198838,NM_198839	.
17	41121205	TGG	T	PTGES3L	NM_001142653,NM_001142654,NM_001261430	.

17	41121208	GGGC	G	PTGES3L	NM_001142653,NM_001142654,NM_001261430	.
17	41121209	GGC	G	PTGES3L	NM_001142653,NM_001142654,NM_001261430	.
17	43319434	TCCGCCG	T	FMNL1	NM_005892	FMNL1:NM_005892:exon15:c.1807_1812del:p.603_604del
17	56321451	GACAC	G	LPO	NM_006151	.
17	56321451	G	GAC	LPO	NM_006151	.
17	58153482	C	CA	HEATR6	NM_022070	.
17	65955758	T	TCCTCCAGCC	BPTF	NM_004459,NM_182641	BPTF:NM_182641:exon24:c.8028_8029insCCTCCAGCC:p.A2676delinsAPPA,BPTF:NM_004459:exon26:c.7977_7978insCCTCCAGCC:p.A2659delinsAPPA
17	72889676	G	GGGCTCCGTAGGTTCCATC	FADS6	NM_178128	FADS6:NM_178128:exon1:c.17_18insGATGGAACCTACGGAGCC:p.P6delinsPMEPTEP
17	74084982	A	ATT	EXOC7	NM_001013839,NM_001145297,NM_001145298,NM_001145299,NM_001282313,NM_015219	EXOC7:NM_001282313:exon8:c.946_947insAA:p.L316fs,EXOC7:NM_015219:exon8:c.976_977insAA:p.L326fs,EXOC7:NM_001013839:exon9:c.1069_1070insAA:p.L357fs,EXOC7:NM_001145298:exon9:c.1045_1046insAA:p.L349fs,EXOC7:NM_001145297:exon10:c.1222_1223insAA:p.L408fs,EXOC7:NM_001145299:exon10:c.1138_1139insAA:p.L380fs
17	79614931	TTTAA	T	TSPAN10	NM_001290212,NM_031945	.
17	80048295	C	CA	FASN	NM_004104	FASN:NM_004104:exon11:c.1825dupT:p.C609fs
18	12819215	TTA	T	PTPN2	NM_001207013	PTPN2:NM_001207013:exon6:c.560_561del:p.L187fs
18	22642709	TAAATAACAAAAAAAA	T	ZNF521	NM_015461	.
18	28739495	C	CAA	DSC1	NM_004948,NM_024421	.
18	43319588	G	GC	SLC14A1	NM_001128588,NM_001146036,NM_001146037,NM_015865	SLC14A1:NM_001146037:exon7:c.1076dupC:p.A359fs,SLC14A1:NM_015865:exon8:c.908dupC:p.

						A303fs,SLC14A1:NM_001128588:exon9:c.1076dupC:p.A359fs,SLC14A1:NM_001146036:exon9:c.908dupC:p.A303fs
18	48723145	CGCCGCCGCG	C	MEX3C	NM_016626	MEX3C:NM_016626:exon1:c.537_545del:p.179_182del
18	72223591	G	GTGC	CNDP1	NM_032649	CNDP1:NM_032649:exon2:c.43_44insTGC:p.V15delinsVL
19	1827020	T	TGGA	REXO1	NM_020695	REXO1:NM_020695:exon2:c.1767_1768insTCC:p.T590delinsST
19	3525878	C	CAA	FZR1	NM_001136197,NM_001136198,NM_016263	FZR1:NM_001136197:exon2:c.82_83insAA:p.R28fs,FZR1:NM_001136198:exon2:c.82_83insAA:p.R28fs,FZR1:NM_016263:exon3:c.82_83insAA:p.R28fs
19	3623676	C	CG	CACTIN	NM_001080543,NM_021231	.
19	4954679	G	GC	UHRF1	NM_001048201,NM_001290050,NM_001290051,NM_001290052,NM_013282	UNKNOWN
19	10097438	G	GAAAA	COL5A3	NM_015719	.
19	14200106	TTCTTCA	T	SAMD1	NM_138352	SAMD1:NM_138352:exon2:c.699_704del:p.233_235del
19	15132675	C	CTT	CCDC105	NM_173482	CCDC105:NM_173482:exon6:c.1195_1196insTT:p.R399fs
19	17397456	GGTGT	G	ANKLE1	NM_001278444	ANKLE1:NM_001278444:exon8:c.1889_1892del:p.G630fs
19	17397491	GTGTGTGTGTT	G	ANKLE1	NM_001278444	ANKLE1:NM_001278444:exon8:c.1924_1933del:p.C642fs
19	17397495	GTGTGTT	G	ANKLE1	NM_001278444	ANKLE1:NM_001278444:exon8:c.1928_1933del:p.643_645del
19	23938360	A	AACAC	ZNF681	NM_138286	.
19	35084497	ATCT	A	SCGB2B2	NM_001025591	SCGB2B2:NM_001025591:exon3:c.251_253del:p.84_85del
19	35758275	G	GGGA	LSR	NM_001260489,NM_001260490,NM_015925,NM_205834,NM_205835	LSR:NM_001260490:exon6:c.1228_1229insGGA:p.G410delinsGR,LSR:NM_205835:exon7:c.1348_

						1349insGGA:p.G450delinsGR,LSR:NM_001260489:exon8:c.1492_1493insGGA:p.G498delinsGR,LSR:NM_015925:exon8:c.1495_1496insGGA:p.G499delinsGR,LSR:NM_205834:exon9:c.1552_1553insGGA:p.G518delinsGR
19	39224336	T	TC	CAPN12	NM_144691	.
19	50155361	GCCGCTC	G	SCAF1	NM_021228	SCAF1:NM_021228:exon7:c.1716_1721del:p.572_574del
19	52887145	TA	T	ZNF880	NM_001145434	ZNF880:NM_001145434:exon4:c.313delA:p.K105fs
19	52887246	T	TCAA	ZNF880	NM_001145434	ZNF880:NM_001145434:exon4:c.413_414insCAA:p.I138delinsIN
19	53117636	GAAGA	G	ZNF83	NM_001105549,NM_001105550,NM_001105551,NM_001105552,NM_001277945,NM_001277946,NM_001277947,NM_001277948,NM_001277949,NM_001277951,NM_001277952,NM_018300	ZNF83:NM_001277951:exon3:c.178_181del:p.S60fs,ZNF83:NM_018300:exon3:c.178_181del:p.S60fs,ZNF83:NM_001105552:exon4:c.178_181del:p.S60fs,ZNF83:NM_001277946:exon4:c.178_181del:p.S60fs,ZNF83:NM_001105550:exon5:c.178_181del:p.S60fs,ZNF83:NM_001105551:exon5:c.178_181del:p.S60fs,ZNF83:NM_001277948:exon5:c.178_181del:p.S60fs,ZNF83:NM_001277949:exon5:c.178_181del:p.S60fs,ZNF83:NM_001277952:exon5:c.178_181del:p.S60fs,ZNF83:NM_001105549:exon6:c.178_181del:p.S60fs,ZNF83:NM_001277945:exon6:c.178_181del:p.S60fs,ZNF83:NM_001277947:exon6:c.178_181del:p.S60fs
19	55324674	C	CA	KIR2DL4	NM_001080772,NM_002255	KIR2DL4:NM_001080772:exon6:c.802dupA:p.S267fs
19	55526103	G	GCAGA	GP6	NM_001083899	GP6:NM_001083899:exon8:c.1209_1210insTCTG:p.P404fs
19	55790886	A	AGCCGCCGCC	HSPBP1	NM_001130106,NM_001297600,NM_012267	HSPBP1:NM_001297600:exon1:c.228_229insGGCGGCGGC:p.S77delinsGGGS,HSPBP1:NM_012267:exon2:c.90_91insGGCGGCGGC:p.S31delinsGGGS,HSPBP1:NM_001130106:exon3:c.90_91insG

						GCGGCGGC:p.S31delinsGGGS
19	56126502	GGCGGCGGC C	G	ZNF865	NM_001195605	ZNF865:NM_001195605:exon1:c.1519_1527del:p. 507_509del
20	238435	CTGGTCT	C	DEFB132	NM_207469	DEFB132:NM_207469:exon1:c.17_22del:p.6_8del
20	271225	T	TA	C20orf96	NM_080571,NM_153269	.
20	271225	T	TTTA	C20orf96	NM_080571,NM_153269	.
20	271225	T	TTA	C20orf96	NM_080571,NM_153269	.
20	278685	AGCC	A	ZCCHC3	NM_033089	ZCCHC3:NM_033089:exon1:c.459_461del:p.153_ 154del
20	2638898	CAAG	C	NOP56	NM_006392	NOP56:NM_006392:exon12:c.1744_1746del:p.582 _582del
20	13561632	T	TAA	TASP1	NM_017714	.
20	31292296	GAC	G	COMMD7	NM_001099339,NM_053041	.
20	31292298	CAA	C	COMMD7	NM_001099339,NM_053041	.
20	32664864	C	CCAG	RALY	NM_007367,NM_016732	RALY:NM_007367:exon7:c.641_642insCAG:p.A2 14delinsAS,RALY:NM_016732:exon8:c.689_690in sCAG:p.A230delinsAS
20	33507379	AGTGT	A	ACSS2	NM_001076552,NM_001242393,NM_ 018677	.
20	33507379	AGTGTGT	A	ACSS2	NM_001076552,NM_001242393,NM_ 018677	.
20	34242573	GGGCACAGG A	G	RBM12	NM_001198838,NM_001198840,NM_0 06047,NM_152838	RBM12:NM_001198840:exon2:c.663_671del:p.22 1_224del,RBM12:NM_001198838:exon3:c.663_67 1del:p.221_224del,RBM12:NM_006047:exon3:c.6 63_671del:p.221_224del,RBM12:NM_152838:exo n3:c.663_671del:p.221_224del
20	42199704	G	GGTGGG TGGGT	SGK2	NM_001199264,NM_016276,NM_170 693	.
20	42199704	G	GGTGGG TGGGTGT	SGK2	NM_001199264,NM_016276,NM_170 693	.
20	42199706	T	TGGGTG G	SGK2	NM_001199264,NM_016276,NM_170 693	.

20	46279836	ACAG	A	NCOA3	NM_001174087,NM_001174088,NM_006534,NM_181659	NCOA3:NM_001174087:exon20:c.3760_3762del:p.1254_1254del,NCOA3:NM_001174088:exon20:c.3736_3738del:p.1246_1246del,NCOA3:NM_006534:exon20:c.3751_3753del:p.1251_1251del,NCOA3:NM_181659:exon20:c.3763_3765del:p.1255_1255del
21	34721704	TTCC	T	IFNAR1	NM_000629	IFNAR1:NM_000629:exon8:c.999_1001del:p.333_334del
21	38569874	T	TGC	TTC3	NM_001001894,NM_003316	TTC3:NM_001001894:exon43:c.5583_5584insGC:p.T1861fs,TTC3:NM_003316:exon43:c.5583_5584insGC:p.T1861fs
21	40883671	G	GAGA	SH3BGR	NM_001001713,NM_007341	SH3BGR:NM_001001713:exon6:c.356_357insAGA:p.G119delinsGE,SH3BGR:NM_007341:exon6:c.689_690insAGA:p.G230delinsGE
21	43708431	G	GC	ABCG1	NM_004915,NM_016818,NM_207174,NM_207627,NM_207628,NM_207629	.
21	46924425	CGGCCCCCCA	C	COL18A1	NM_030582,NM_130444,NM_130445	COL18A1:NM_030582:exon33:c.3364_3364del:p.G1122fs,COL18A1:NM_130444:exon33:c.4069_4069del:p.G1357fs,COL18A1:NM_130445:exon34:c.2824_2824del:p.G942fs
22	22868572	CA	C	ZNF280A	NM_080740	ZNF280A:NM_080740:exon2:c.1382delT:p.L461X
22	23481133	GAA	G	RSPH14	NM_014433	.
22	26879946	GGAGGCGGCGCCCCGGGGGAGA	G	SRRD	NM_001013694	SRRD:NM_001013694:exon1:c.91_111del:p.31_37del
22	29885567	A	AAAGTCCCCTGAGAAGGCC	NEFH	NM_021076	NEFH:NM_021076:exon4:c.1938_1939insAAGTCCCCTGAGAAGGCC:p.A646delinsAKSPEKA
22	29885598	AAGGAAG	A	NEFH	NM_021076	NEFH:NM_021076:exon4:c.1970_1975del:p.657_659del
22	37964408	CCAGCGCCTGCTGCAAAC	C	CDC42EP1	NM_152243	CDC42EP1:NM_152243:exon3:c.758_778del:p.253_260del

		CCCT				
22	38483174	A	ACATGG AGGT	BAIAP2L2	NM_025045	BAIAP2L2:NM_025045:exon11:c.1215_1216insACCTCCATG:p.S406delinsTSMS
22	39777824	A	AACC	SYNGR1	NM_004711	SYNGR1:NM_004711:exon4:c.607_608insACC:p.T203delinsNP
X	8763309	GCTGCTGCTG CTGCGGCTT	G	FAM9A	NM_001171186,NM_174951	FAM9A:NM_001171186:exon7:c.623_640del:p.208_214del,FAM9A:NM_174951:exon7:c.623_640del:p.208_214del
X	8763327	T	TCTG	FAM9A	NM_001171186,NM_174951	FAM9A:NM_001171186:exon7:c.622_623insCAG:p.E208delinsAE,FAM9A:NM_174951:exon7:c.622_623insCAG:p.E208delinsAE
X	18582646	GAT	G	CDKL5	NM_001037343,NM_003159	.
X	24382373	TTGCTGCTGC	T	SUPT20HL1	NM_001136234	SUPT20HL1:NM_001136234:exon1:c.1497_1505del:p.499_502del
X	37981355	AAAG	A	SYTL5	NM_001163334,NM_001163335,NM_138780	SYTL5:NM_001163334:exon15:c.1795_1797del:p.599_599del,SYTL5:NM_138780:exon15:c.1729_1731del:p.577_577del,SYTL5:NM_001163335:exon16:c.1729_1731del:p.577_577del
X	50121146	A	AG	DGKK	NM_001013742	UNKNOWN
X	56591830	CCCCATAGGC CCCATTGGG	C	UBQLN2	NM_013444	UBQLN2:NM_013444:exon1:c.1525_1542del:p.509_514del
X	66765158	T	TGCAGC A	AR	NM_000044	AR:NM_000044:exon1:c.170_171insGCAGCA:p.L57delinsLQQ
X	104464281	TC	T	TEX13A	NM_001291277,NM_031274	.
X	150349554	AACCCACCA CTGG	A	GPR50	NM_004224	GPR50:NM_004224:exon2:c.1500_1511del:p.500_504del
X	150349557	CCACCACTG GCCA	C	GPR50	NM_004224	GPR50:NM_004224:exon2:c.1503_1514del:p.501_505del
X	152719969	GGGGA	G	HAUS7	NM_017518	.

Supplementary Table S5: Candidate variants in transcribed region after further filtered by ModelR and Phenolyzer analysis

Reasom for exclusion	CHROM	POS	REF	ALT	GeneName	Func	Gene	ExonicFunc	AAChange
benign prediction	X	48369847	G	A	PORCN	exonic	NM_001282167,NM_022825,NM_203473,NM_203474,NM_203475	missense SNV	PORCN:NM_203474:exon2:c.G301A:p.V101I,PORCN:NM_001282167:exon3:c.G88A:p.V30I,PORCN:NM_022825:exon3:c.G301A:p.V101I,PORCN:NM_203473:exon3:c.G301A:p.V101I,PORCN:NM_203475:exon3:c.G301A:p.V101I
Sanger Sequencing	2	233712226	C	CGCA	GIGYF2	exonic	NM_001103146,NM_001103147,NM_001103148,NM_015575	nonframeshift insertion	GIGYF2:NM_001103148:exon26:c.3611_3612insGCA:p.P1204delinsPQ,GIGYF2:NM_001103146:exon27:c.3629_3630insGCA:p.P1210delinsPQ,GIGYF2:NM_001103147:exon29:c.3692_3693insGCA:p.P1231delinsPQ,GIGYF2:NM_015575:exon29:c.3629_3630insGCA:p.P1210delinsPQ
Sanger Sequencing	9	131020792	ACCG	A	GOLGA2	exonic	NM_004486	nonframeshift deletion	GOLGA2:NM_004486:exon21:c.2147_2149del:p.716_717del
Sanger Sequencing	11	6411935	TGCTGGC	T	SMPD1	exonic	NM_000543,NM_001007593	nonframeshift deletion	SMPD1:NM_000543:exon1:c.108_113del:p.36_38del,SMPD1:NM_001007593:exon1:c.108_113del:p.36_38del
Sanger Sequencing	11	4592706	T	TAC	C11orf40	exonic	NM_144663	frameshift insertion	C11orf40:NM_144663:exon4:c.600_601insGT:p.M201fs
NA	2	11944690	A	C	LPIN1	exonic	NM_001261427,NM_001261428,NM_145693	missense SNV	LPIN1:NM_145693:exon15:c.A2047C:p.I683L,LPIN1:NM_001261427:exon16:c.A2065C:p.I689L,LPIN1:NM_001261428:exon17:c.A2302C:p.I768L
NA	2	11955273	G	A	LPIN1	exonic	NM_001261427,NM_001261428,NM_145693	missense SNV	LPIN1:NM_145693:exon17:c.G2201A:p.R734Q,LPIN1:NM_001261427:exon18:c.G2219A:p.R740Q,LPIN1:NM_001261428:exon19:c.G2456A:p.R819Q
